# Selective sugar transport supports *Proteus mirabilis* fitness in the urinary tract

**DOI:** 10.1371/journal.ppat.1014324

**Published:** 2026-06-23

**Authors:** Allyson E. Shea, Shiuhyang Kuo, Surbhi Gupta, Sara N. Smith, Trishna Appaji, Taylor Mitchell, Harry L. T. Mobley, Melanie M. Pearson

**Affiliations:** 1 University of South Alabama, College of Medicine, Department of Microbiology and Immunology, Mobile, Alabama, United States of America; 2 University of Michigan Medical School, Department of Microbiology and Immunology, Ann Arbor, Michigan, United States of America; University of Utah, UNITED STATES OF AMERICA

## Abstract

*Proteus mirabilis* is a leading cause of complicated urinary tract infections (UTIs). Prior work showed *P. mirabilis* metabolizes sugars during experimental UTI, yet the role of sugar import systems in pathogenesis remains poorly defined. To investigate this, we generated a panel of 47 targeted mutants in predicted sugar transporter genes and assessed their growth *in vitro* and fitness *in vivo*. Growth screening in nutrient-rich and minimal media revealed carbon source-dependent defects in several phosphotransferase system (PTS) mutants, including *ptsH* and *ptsI*. Pooled insertion sequencing (In-seq) identified *xapB*, *ptsH*, and *ptsI* as *in vivo* fitness factors, with validation in a traditional murine co-challenge model. Functional studies showed that *xapB*, annotated as a xanthosine permease, did not support xanthosine or guanosine uptake in *P. mirabilis*, suggesting misannotation. Dissection of the PTS network revealed that a triple mutant lacking *scrA*, *ulaC*, and *ptsG* recapitulated the *ptsH* phenotype *in vivo*. To evaluate whether increased sugar availability exacerbates these defects, we modeled glucosuria using the SGLT2 inhibitor dapagliflozin in CBA/J mice. Dapagliflozin treatment significantly increased urinary glucose and enhanced *P. mirabilis* colonization. There was an inverse correlation between colonization and urinary glucose, but only in untreated mice. These findings reveal limitations in genome-based transporter annotation, establish a functional link between sugar import and *P. mirabilis* fitness during UTI, and demonstrate that host metabolic conditions such as glucosuria can influence the severity of infection.

## Author summary

All living organisms require nutrients to grow, survive, and cause disease. Bacteria like *Proteus mirabilis*, which causes urinary tract infections, rely on specialized systems to import and metabolize sugars available in the host environment. In this study, we systematically disrupted 47 genes predicted to encode sugar transporters in *P. mirabilis* and tested their contribution to infection in a mouse model. We identified three key genes (*xapB*, *ptsH*, and *ptsI*) that were critical for colonization. Further analysis showed that many sugar transporters in *P. mirabilis* were misannotated, and predicted substrates like sucrose and cellobiose were not utilized by the bacterium. We also demonstrated that high sugar conditions, mimicking diabetic urine using the drug dapagliflozin, worsened infection and increased disease severity. These results highlight the importance of carbohydrate acquisition for *P. mirabilis* during infection and emphasize the need to experimentally validate gene function rather than rely on predictions based on other bacteria like *E. coli*.

## Introduction

Urinary tract infections (UTIs) are among the most common bacterial infections, disproportionately affecting women and generating a substantial financial burden on the healthcare system [1,2]. In catheterized patients, UTIs are often polymicrobial and more likely to involve urease-producing species such as *Proteus mirabilis* [3,4]. This organism poses a particular clinical challenge in long-term catheterization, where its ability to hydrolyze urea raises urine pH, leading to the precipitation of magnesium ammonium phosphate (struvite) and calcium phosphate into crystalline deposits [5,6]. These deposits form kidney and bladder stones and obstruct catheters, contributing to recurrent infection, inflammation, and tissue damage [7,8]. Although well-studied in terms of urease activity, swarming motility, and fimbrial adhesion, the metabolic strategies used by *P. mirabilis* during infection remain incompletely understood.

Amino acids are the major carbon source available to microbes in the urinary tract and can be detected in gram quantities in the total daily urine output from healthy adults [9,10]. It is then logical that uropathogens would shift their resources to acquiring amino acids as a food source. This is indeed the case for uropathogenic *Escherichia coli* (UPEC), the major agent of uncomplicated UTI, which not only upregulates amino acid importers [11], but also possess greater redundancy in those systems compared to non-urinary isolates [12]. This is in contrast to commensal *E. coli* in the gastrointestinal tract, which relies more heavily on mono- and disaccharides for growth [13]. These types of carbohydrates in the gut are in relatively low abundance in the urine [14–16].

Perhaps counterintuitively given this nutrient landscape, *P. mirabilis* activates a variety of metabolic and nutrient acquisition pathways during experimental UTI, including amino acid uptake, peptide transport, key enzymes of central metabolism, and, in particular, sugar uptake and catabolism [17]. Specifically, the loss of glycolytic enzymes and the oxidative pentose phosphate pathway significantly reduce *P. mirabilis* colonization and fitness *in vivo* [18,19]. Genes in both the non-oxidative pentose phosphate pathway and phosphoglycerate kinase (*pgk*) have been deemed possible essential genes [19]. This is in contrast to UPEC, where glycolytic genes are not induced during UTI and the loss of glycolysis had no effect; instead, gluconeogenesis is exclusively required [18,20,21]. In fact, pathways involved in sugar import and catabolism were downregulated in UPEC collected directly from women with active UTI, while amino acid and peptide importers were upregulated [11]. Collectively, this suggests that *P. mirabilis* has access to carbohydrate sources either in urine or from host cells within the urinary tract that confer an advantage during polymicrobial infection.

Diabetes is one prevalent health condition that results in an increase of available sugars in urine. Diabetic patients are at increased risk for contracting UTI [22] and a larger proportion of infections are caused by *P. mirabilis* [23]. Sodium-glucose cotransporter 2 (SGLT2) is a common biological target to prevent the re-uptake of urinary glucose in diseases like diabetes, but also chronic kidney disease and heart failure [24]. Use of SGLT2 inhibitors, such as dapagliflozin or canagliflozin, has been shown to increase bacterial burden in some murine models of UTI [25,26]. In particular, UPEC loads were significantly higher in the bladders and kidneys of dapagliflozin-treated mice, with elevated rates of dissemination to the spleen and liver observed at 48 hours post-inoculation [25]. These preclinical findings suggest that elevated urinary glucose may worsen infection outcomes. We hypothesized that this effect would be more pronounced for *P. mirabilis*, which exhibits a greater dependence on glucose-driven metabolism during UTI.

To understand how *P. mirabilis* imports sugars during infection, we focused our study on sugar uptake systems. The best characterized family of these is the phosphoenolpyruvate (PEP) phosphotransferase system (PTS). The PTS comprises upstream regulatory proteins, including Enzyme I (EI) and the phosphocarrier protein HPr, which coordinate phosphorylation cascades that activate a wide array of sugar-specific importers [27]. The recipients of this phosphorylation are the EII units, which coordinate to facilitate the uptake of one substrate or a small group of similarly related substrates [28,29]. In addition to importing sugars and sugar derivatives, this system has regulatory crosstalk related to nitrogen metabolism [30,31], chemotaxis [32,33], and virulence in some pathogens [28]. In *P. mirabilis*, the identity of certain EII components and the corresponding predicted substrates relies heavily on annotations from other Gram-negative bacterial species. Given this, coupled with the known requirement for glycolysis during *P. mirabilis* colonization and virulence *in vivo*, we sought to better define the specific carbon sources fueling uropathogenicity.

Toward this goal, we generated a set of 47 mutants targeting predicted sugar importers and tested them in a murine model of UTI. Three mutants, including a putative xanthosine permease (*xapB*) and PTS core components *ptsH* and *ptsI*, exhibited significant *in vivo* fitness defects. A combination of three PTS substrate-specific mutations was sufficient to phenocopy the *ptsH* mutant *in vivo*, confirming that these systems collectively contribute to PTS-dependent fitness. Finally, using an SGLT2 inhibitor to induce hyperglucosuria in mice, we observed elevated bacterial burden and increased morbidity during experimental UTI. We also identified an inverse correlation between bacterial burden and urinary glucose in untreated mice that was abolished during hyperglucosuria from SGLT2 inhibition. Collectively, our data show that specific sugar uptake systems are required for *P. mirabilis* fitness during UTI, and glucosuria alters experimental UTI outcomes.

## Results

### Generation and growth assessment of sugar transporter mutants in *Proteus mirabilis*

Glycolysis and related carbohydrate pathways have been shown to play a central role in *P. mirabilis* pathogenesis during urinary tract infection (UTI) [17,18]. To further define the contribution of sugar import and metabolism to bacterial fitness, we constructed a panel of targeted mutants in genes predicted to encode sugar transporters. Candidate genes were selected using multiple complementary approaches. First, phosphotransferase system (PTS) components were prioritized due to their known roles in carbohydrate uptake. In parallel, bioinformatic tools including KEGG [34], Transporter Classification Database (TCDB 2.0) [35], NCBI BLAST against sugar transporters encoded by UPEC CFT073 [36], NCBI Conserved Domains Database [37], Phyre2 [38], and the Bacterial and Viral Bioinformatics Resource Center (BV-BRC) [39] were used to identify additional sugar transport genes from ones annotated in the *P. mirabilis* HI4320 genome [40]. To cast a wide net, we used a broad definition of sugar such that a prediction for sugar transport in any one database merited inclusion. This yielded a final list of 50 targets (Table 1). Mutants were generated using the targetron system for site-specific group II intron insertion [41,42]. Of the 50 genes targeted, we successfully created 47 mutants, each confirmed by PCR.

**Table 1:** Candidate *P. mirabilis* sugar transporter genes.

ORF	NCBI	Name	Annotation^*a*^	Mutant	Type	Pool
PMI0090	PMI_RS00430	*rbsB*	D-ribose ABC transporter, substrate-binding periplasmic protein	Y	ABC	1
PMI0091	PMI_RS00435	*rbsC*	ribose ABC transporter, permease protein	Y	ABC	1
PMI0092	PMI_RS00440	*rbsA*	ribose ABC transporter, ATP-binding protein	Y	ABC	1
PMI0093	PMI_RS00445	*rbsD*	high affinity ribose transport protein	Y	ABC	1
PMI0164	PMI_RS00795		MFS-family transporter	Y	MFS	1
PMI0291	PMI_RS01405	*treB*	PTS system, trehalose-specific IIBC component	Y	PTS	2
PMI0455	PMI_RS02280	*nagE*	pts system, N-acetylglucosamine-specific EIICBA component	Y	PTS	2
PMI0775	PMI_RS03810	*uup*	ABC transporter ATP-binding protein	Y	ABC	1
PMI0850	PMI_RS04170		MFS-family transporter	Y	MFS	1
PMI1175	PMI_RS05670	*fetB*	putative membrane protein	Y	ABC	1
PMI1176	PMI_RS05675		ABC transporter, ATP-binding protein	Y	ABC	1
PMI1224	PMI_RS05905		putative ABC-type multidrug transport system	Y	ABC	1
PMI1251	PMI_RS06040		MFS-family transporter	N	MFS	n/a
PMI1254	PMI_RS06055		MFS-family transporter	Y	MFS	1
PMI1515	PMI_RS07345	*sotB*	sugar efflux transporter	Y	MFS	1
PMI1570	PMI_RS07650	*xapB*	xanthosine permease (MFS-family transporter)	Y	MFS	1
PMI1775	PMI_RS08705	*ulaA*	putative ascorbate PTS system IIC component	Y	PTS	2
PMI1776	PMI_RS08710	*ulaB*	PTS system IIB component	Y	PTS	2^*b*^
PMI1777	PMI_RS08715	*ulaC*	PTS system IIA component	Y	PTS	2
PMI1828	PMI_RS09020	*ptsH*	PTS system phosphocarrier protein	Y	PTS	2
PMI1829	PMI_RS09025	*ptsI*	phosphoenolpyruvate-protein phosphotransferase	Y	PTS	2
PMI1830	PMI_RS09030	*crr*	PTS family enzyme IIA component/glucose-specific phosphotransferase enzyme IIA component	Y	PTS	2
PMI1935	PMI_RS09535		L-lactate permease	Y	Others	2
PMI1943	PMI_RS09575		MFS-family transporter	N	MFS	n/a
PMI1946	PMI_RS09590	*sglT*	sodium/glucose contransporter	Y	Others	2
PMI2101	PMI_RS10350	*bcsC*	cellulose synthase protein	Y	Others	2
PMI2135	PMI_RS10520	*agaF*	putative N-acetylgalactosamine-specific PTS system, IIA component	Y	PTS	2
PMI2136	PMI_RS10525	*agaD*	putative N-acetylgalactosamine-specific PTS system, IID component	Y	PTS	2
PMI2137	PMI_RS10530	*agaW*	putative N-acetylgalactosamine-specific PTS system, IIC component	Y	PTS	2
PMI2138	PMI_RS10535	*agaV*	putative N-acetylgalactosamine-specific PTS system, IIB component	Y	PTS	2
PMI2157	PMI_RS10630	*fruK*	1-phosphofructokinase	Y	PTS	2
PMI2198	PMI_RS10830		MFS-family transporter	Y	MFS	1
PMI2226	PMI_RS10970	*bglF*	PTS system, IIabc component/PTS system beta-glucoside-specific IIA component, Glc family	Y	PTS	2
PMI2292	PMI_RS11320	*ptsG*	glucose-specific PTS system, EIIBC component	Y	PTS	2
PMI2597	PMI_RS12825	*nrpX*	MFS-family transporter	Y	MFS	1
PMI2649	PMI_RS13050		MFS-family transporter	Y	MFS	1
PMI2954	PMI_RS14605	*chbA*	N,N’-diacetylchitobiose-specific PTS system, IIa component	Y	PTS	2
PMI2955	PMI_RS14610	*chbC*	N,N’- diacetylchitobiose-specific PTS system, EIIc component	Y	PTS	2
PMI2956	PMI_RS14615	*chbB*	N,N’- diacetylchitobiose-specific PTS system, EIIb component	Y	PTS	2
PMI2967	PMI_RS14670		ABC-type sugar transport system, periplasmic component	Y	ABC	1
PMI2969	PMI_RS14680		MFS-family transporter	Y	MFS	1
PMI2982	PMI_RS14745	*malX*	PTS system, EIIBC component, putative maltose	Y	PTS	2
PMI3099	PMI_RS15340		putative cellulose biosynthesis protein C	Y	Others	2
PMI3515	PMI_RS17475	*scrA*	putative sucrose PTS system IIBC component	Y	PTS	2
PMI3614	PMI_RS17985	*ugpC*	sn-glycerol-3-phosphate ABC transporter, ATP-binding protein	Y	ABC	1
PMI3615	PMI_RS17990	*ugpE*	sn-glycerol-3-phosphate ABC transporter, permease protein	Y	ABC	1
PMI3616	PMI_RS17995	*ugpA*	sn-glycerol-3-phosphate ABC transporter, permease protein	Y	ABC	1
PMI3617	PMI_RS18000	*ugpB*	glycerol-3-phosphate ABC transporter, substrate-binding protein	Y	ABC	1
PMI3646	PMI_RS18140	*ptsN*	nitrogen regulatory protein [includes: PTS system EIIA component]	N	PTS	n/a
PMI3705	PMI_RS18435		putative ABC transporter ATP-binding protein	Y	ABC	1

^*a*^Annotations are from original Sanger Centre predictions or from KEGG.

^*b*^PMI1776 was inadvertently left out of pool 2, and PMI1176 was included in both pools.

**Fig 1:**
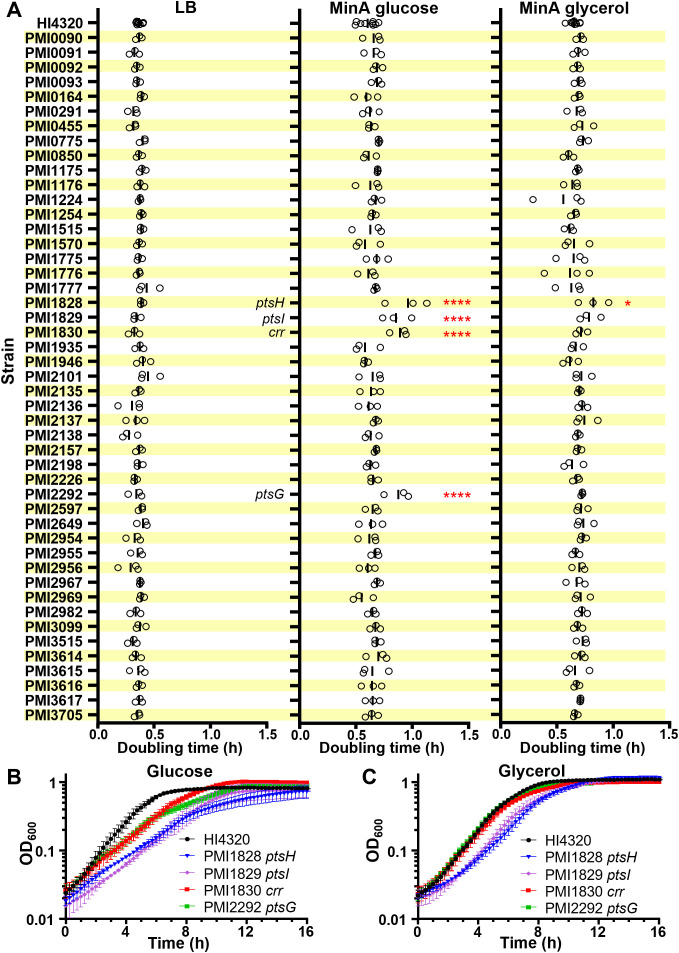
Growth parameters of 47 sugar transporter mutants. **(A)** Doubling time measurements. Predicted gene names and annotations may be cross-referenced in Table 1. Left, LB; middle, Minimal A with 0.2% glucose as the carbon source; right, Minimal A with 0.2% glycerol as the carbon source. Vertical lines show means. *P < 0.05, ****P < 0.0001; one-way ANOVA *vs.* wild type HI4320 with Dunnett’s multiple comparisons test. **B-C,** Growth curves for the four PTS mutants with growth defects in Minimal A. **(B)** Minimal A with 0.2% glucose as the carbon source. **(C)** Minimal A with 0.2% glycerol as the carbon source. Error bars = SEM (n ≥ 3).

We examined the growth of all 47 sugar transporter mutants in LB and in Minimal A medium supplemented with either 0.2% glucose or 0.2% glycerol as the sole carbon source (Fig 1). Doubling time during logarithmic phase was calculated and compared to wild-type HI4320 (Fig 1A). As expected, all mutants exhibited robust growth in LB, indicating that disruption of sugar-specific transport systems did not impair viability under nutrient-rich conditions. In contrast, four mutants displayed a statistically significant increase in doubling time when cultured in Minimal A with glucose as the carbon source (Fig 1B), and all of them were in PTS-related genes. The *ptsG* (PMI2292) mutant, encoding the EIIBC component of the glucose-specific PTS system [43], showed reduced growth (doubling time 52.63 min *vs.* 36.22 min for wt, *P* < 0.0001), consistent with its role in glucose import. Similarly, mutants lacking *crr* (PMI1830, 53.41 min, *P* < 0.0001) and *ptsI* (PMI1829, 51.23 min, *P* = 0.0005), which encode the carbohydrate repression resistance protein and Enzyme I of the PTS [44], respectively, were attenuated under these conditions. Notably, the *ptsH* (PMI1830) mutant, which lacks the general phosphocarrier protein HPr, exhibited impaired growth in both glucose- (57.75 min, *P* < 0.0001) and glycerol- (49.28 min *vs.* 39.15 min for wt, *P* = 0.0441) containing Minimal A medium (Fig 1B-1C). While these growth defects in glucose were expected because glucose is a well-characterized PTS substrate, the defect in glycerol for the *ptsH* mutant was not anticipated. We also observed a modest decrease in growth parameters for the *ptsI* mutant in glycerol (Fig 1C), but this was not statistically significant (47.08 min, *P* = 0.2959). Growth of all four PTS mutants was restored in both glucose and glycerol following genetic complementation (S1 Fig). The *ptsH* growth defect in Minimal A was partially rescued by *ptsH in trans*, but fully restored with *ptsHI*, consistent with their expression as an operon. These findings align with the functional hierarchy of the PTS, as PtsI and PtsH comprise the phosphorelay for multiple sugar import systems and serve as central nodes for carbohydrate uptake and regulation in *P. mirabilis*, while Crr is paired with several substrate-specific importers including PtsG).

### *In vivo* screening identifies *xapB*, *ptsH*, and *ptsI* as fitness factors

**Fig 2:**
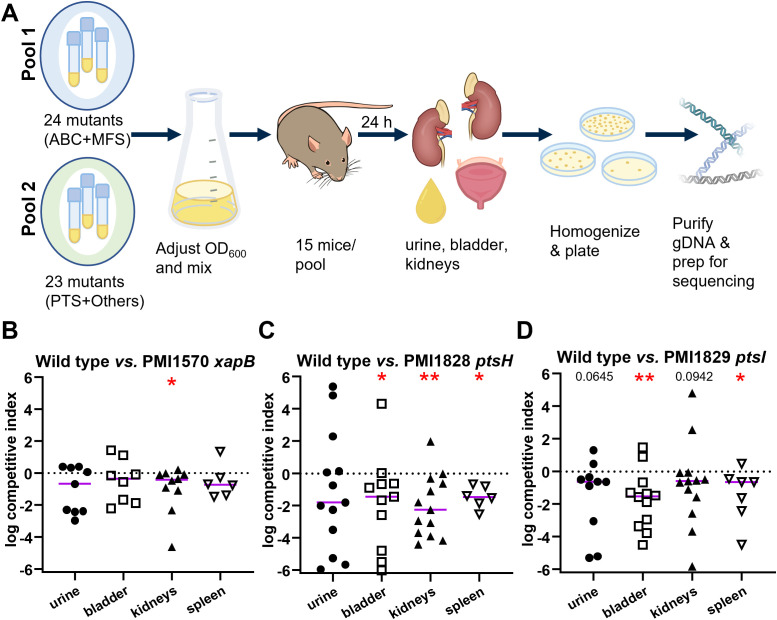
Identification of sugar importers that contribute to fitness during UTI. **(A)** Experimental workflow for In-seq screening *P. mirabilis* sugar transporter mutants in mice. **B-D**, 1:1 wild type:mutant co-challenge competitive indices (7 dpi) for mutants identified in the In-seq screen. (**B**) wild type *vs. xapB* (n = 10); (**C**) wild type *vs. ptsH* (n = 15); (**D**) wild type *vs. ptsI* (n = 15). Horizontal lines show medians. Dashed line indicates equal fitness of wild type and mutant (log CI = 0). *P < 0.05; **P < 0.01; exact value given for 0.10 > *P* > 0.05, one sample Wilcoxon test *vs.* a theoretical median of 0.

Previous work established that the bottleneck for *P. mirabilis* HI4320 in the murine model of ascending UTI limits reliable input pools to approximately 25 mutants [45]. Based on this constraint, we divided our panel of 47 sugar metabolism mutants into two pools for *in vivo* testing (Table 1). Pool 1 consisted of 24 mutants from the ATP-binding cassette (ABC) and major facilitator superfamily (MFS) transporter families. Pool 2 included 23 mutants from the phosphotransferase system (PTS) and other single-gene predicted sugar transporters (Fig 2A). Each pool was independently inoculated into the bladders of female CBA/J mice, and infection was allowed to proceed for 24 hours. Urine, bladder, and kidney samples were then harvested and individually barcoded for downstream sequencing (S1 Table). The abundance of each mutant in the inoculum and recovered organs was determined using an insertion sequencing (In-seq) strategy (S1 and S2 Datasets; GEO accession #GSE244606). An aliquot of each pool was plated to quantify the total input CFU and was also sequenced to identify variation that could have occurred from plating and outgrowth; no notable variation was observed in these control samples (S1 and S2 Datasets, input “I” data).

From Pool 1, the *xapB* (PMI1570) mutant emerged as the only strain with a statistically significant fitness defect (S2 Fig). This mutant was underrepresented exclusively in the kidneys, suggesting an organ-specific role (S2C Fig). *xapB* encodes a putative xanthosine permease of the MFS family, based on homology to the *E. coli* XapB transporter as predicted by KEGG [46,47]. From Pool 2, both *ptsH* and *ptsI* mutants were depleted in the bladder, and *ptsH* also showed a defect in the kidneys (S3 Fig). The 24-hour time point for In-seq was selected based on documented bottleneck effects in the murine UTI model [48,49]. In this pooled approach, mutants were assessed in the presence of 22–23 other mutants without a wild-type control. To validate the *in vivo* In-seq findings, each mutant (*xapB*, *ptsH*, and *ptsI*) was individually assessed in the established 7-day murine co-challenge model [50]. For each strain, mice were inoculated transurethrally with a 1:1 mixture of mutant and wild-type HI4320, and competitive indices (CI) were calculated for urine, bladder, kidneys, and, to gauge dissemination into the bloodstream, spleens. The *xapB* mutant displayed a significant colonization defect in the kidneys (median log_10_ CI -0.4177, *P* = 0.0137) (Figs 2B, S4A), consistent with the original In-seq data. The *ptsH* mutant exhibited significant defects in the bladder, kidney, and spleen (median log_10_ CI -1.459, -2.261, and -1.469, and *P* = 0.0322, 0.0034, and 0.0312, respectively), while *ptsI* was significantly outcompeted in the bladder and spleen (median log_10_ CI -1.538 and -0.6682, and *P* = 0.0093 and 0.0469, respectively) (Figs 2C-2D, S4B–S4C). Collectively, these experiments screened 47 sugar transport mutants for *in vivo* fitness and identified organ-specific defects that were reproducible in the traditional ascending UTI murine model.

### Functional characterization of putative xanthosine transporter XapB

**Fig 3:**
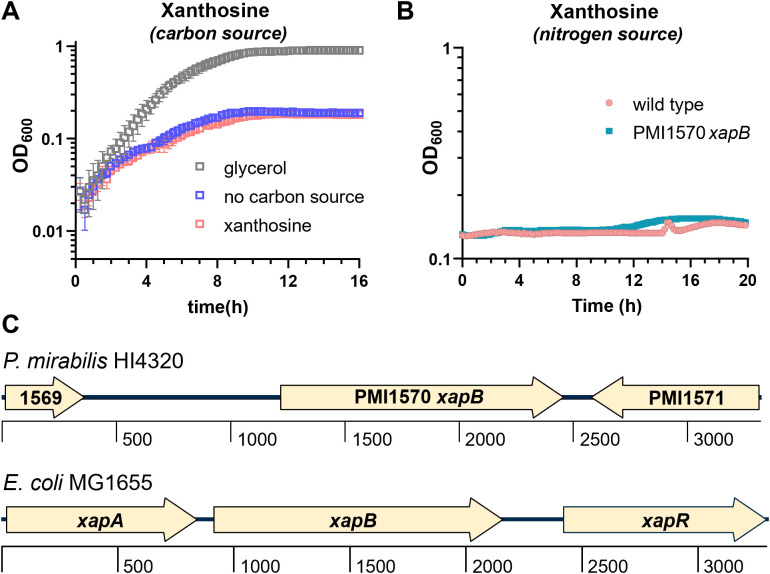
PMI1570 is likely misannotated as a xanthosine transporter. **(A)** Growth curves for wild-type HI4320 in Minimal A with no carbon source, 0.2% glycerol, or 0.1% xanthosine as a sole carbon source (n = 3; error bars show SD). **(B)** Growth on xanthosine as sole nitrogen source (Biolog plate PM3). **(C)** The genetic organization of the putative *xap* locus in *P. mirabilis* (top) is missing elements from the *xap* locus in *E. coli*, where it has been characterized.

To investigate the function of *xapB* and the other In-seq hits (*ptsH* and *ptsI*), we performed targeted phenotypic analyses guided by predicted functions of each transporter. We first focused on PMI1570, the putative xanthosine permease (*xapB*). When Minimal A medium was used with 0.1% xanthosine as the sole carbon source, HI4320 failed to grow (Fig 3A). We therefore screened growth across Biolog Phenotype MicroArray carbon and nitrogen source plates to identify additional potential substrates in an unbiased fashion (S3 Dataset). No growth was observed when xanthosine was provided as a sole nitrogen source after 20 hours of incubation, indicating that strain HI4320 was unable to utilize xanthosine in this context (Fig 3B). These findings suggest that XapB may not function as a xanthosine importer in *P. mirabilis* under the tested conditions. However, ruling xanthosine out as a carbon or nitrogen source does not exclude possibility for import.

Further analysis of the genomic context revealed notable differences in operon structure compared to the *E. coli* MG1655 *xap* locus (Fig 3C). In *P. mirabilis*, both *xapA*, encoding a xanthosine phosphorylase (PNP-II), and the regulatory gene *xapR* are absent. This single-gene organization raises the likelihood that XapB transports an alternate substrate. Beyond KEGG and BV-BRC’s annotations as a xanthosine transporter, other databases predict *xapB* to encode an undefined nucleoside transporter (BLAST and BioCyc). While *P. mirabilis* HI4320 XapB is 49% identical and 70% similar to *E. coli* MG1655 XapB, it is also 44% identical and 65% similar to the nucleoside:H^+^ symporter NupG. Biolog data suggested differential growth for wild-type HI4320 and the *xapB* mutant on guanosine (S5A–S5B Fig); however, the increase in optical density appeared to be due to a chemical precipitate. To confirm the turbidity was due to precipitate and not outgrowth, we plated the contents of the guanosine wells after the experimental endpoint and no CFU were recovered. There were no other notable differences in growth across 251 unique substrates between wild type and *xapB*. Nevertheless, we hypothesized that guanosine might enter through XapB.

**Fig 4:**
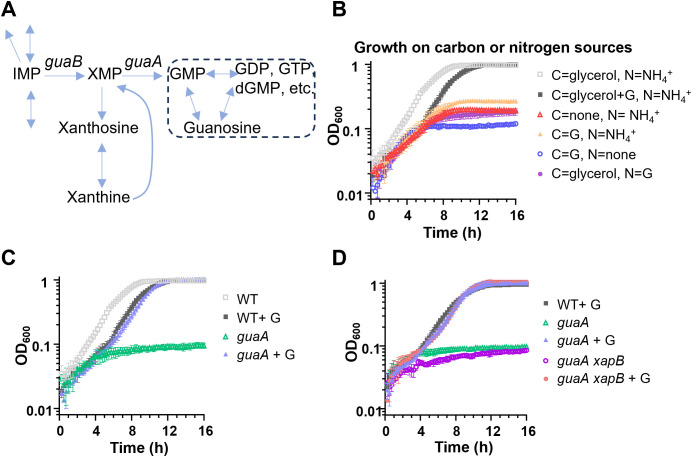
PMI1570 is not likely to be a guanosine transporter. **(A)** Prediction of biochemical pathways encoded by *P. mirabilis* HI4320 including xanthosine and guanosine. *P mirabilis* is not predicted to be able to convert guanosine or related molecules into central metabolism (denoted by dashed outline). **(B)** Growth curves in Minimal A indicated that wild-type HI4320 can’t use guanosine (G, 0.25 mg/mL) as a sole carbon (C) or nitrogen (N) source. **C-D,** Growth curves in Minimal A containing 0.2% glycerol. **(C)** HI4320 can import guanosine to chemically complement the *guaA* mutant (wild type data are the same as in B). **(D)** A *guaA xapB* double mutant can still be chemically complemented by guanosine, indicating that *xapB* probably doesn’t transport guanosine. In these experiments, guanosine was added at a final concentration of 0.05 mg/mL. For sole nitrogen source growth curves, n = 2. For all other conditions, n = 3. Error bars show SD.

Our group previously reported that a *guaA* mutant, which is deficient in GMP biosynthesis, exhibited impaired growth on murine organ agar, a severe defect *in vivo*, altered swarming motility, and a nutritional defect that could be rescued by exogenous RNA [45]. As predicted, HI4320 was unable to use guanosine as either a sole nitrogen or carbon source in Minimal A medium (Fig 4A, 4B). Despite this, exogenous guanosine successfully complemented the growth defect of the *guaA* mutant in Minimal A with glycerol, confirming the presence of a functional guanosine uptake system (Fig 4C). A modest slowing of growth in the presence of guanosine was not due to the DMSO solvent, nor could this slowing be mitigated by using a lower concentration of guanosine (S5C–S5D Fig). To determine whether XapB was responsible for this activity, we constructed a *guaA*/*xapB* double mutant. As with the single *guaA* mutant, growth defects were rescued by guanosine supplementation (Fig 4D). These data indicate that guanosine can enter the cell independent of XapB. The actual substrate of XapB, which must be available in the urinary tract and contributes to *P. mirabilis* fitness, remains to be determined.

### Dissecting the role of PTS components in *P. mirabilis* urinary tract fitness

**Fig 5:**
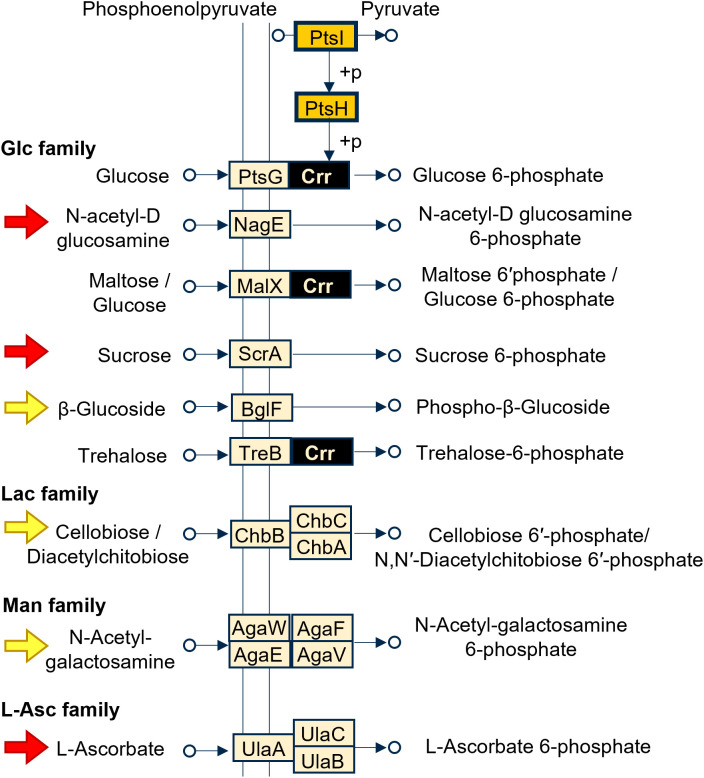
KEGG predictions for PTS-family transporters in *P. mirabilis* HI4320. Nine substrate-specific importers, shown here spanning the cell membrane depicted as parallel lines, are predicted to rely on the PtsHI phosphorelay. Crr (PTS enzyme IIA component) is predicted to interact with three substrate-specific importers and is highlighted in black. Arrows indicate two sets of non-*crr*-dependent triple mutants (red and yellow) based on *in vivo* expression data shown in Fig 7A that were tested in mice.

The phosphotransferase system (PTS) is essential for carbohydrate uptake and consists of a relay of proteins that transfer phosphate from phosphoenolpyruvate (PEP) to sugar-specific permeases. In *P. mirabilis*, the PTS system appears to be organized similar to other Enterobacterales: *ptsI* encodes Enzyme I, which initiates the phospho-transfer cascade, and *ptsH* encodes HPr, a central phosphocarrier that relays phosphate to multiple specific import systems [51]. *P. mirabilis* HI4320 is predicted to encode nine of these substrate-specific PTS importers (Fig 5). Our *in vivo* co-challenge studies showed that both *ptsI* and *ptsH* single mutants were significantly outcompeted by wild type (Fig 2). These broad defects suggested that loss of general PTS function impairs fitness, but the specific transporter systems, and thus substrates, responsible remained unclear. Of the nine importers, only glucose transporter *ptsG* had been experimentally confirmed for *P. mirabilis* HI4320 (Fig 1B). To identify other PTS substrates that could contribute to the *ptsH* and *ptsI* fitness defects, we assessed growth of wild-type HI4320 and the *ptsH* mutant on 190 carbon sources and 95 nitrogen sources (S3 Dataset).

**Fig 6:**
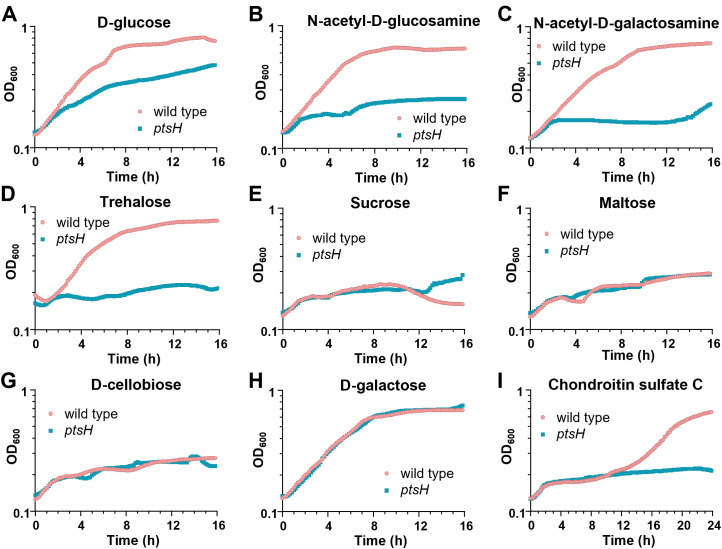
Notable carbon source growth curve results for wild type *vs.* the *ptsH* mutant. **A-H**, Predicted PTS substrates. **A-D,** Predicted PTS substrates that showed expected growth defects for the *ptsH* mutant. **E-G,** Predicted PTS substrates that did not support growth by wild-type HI4320, suggesting that their transporters might be misannotated. **(H)** *P. mirabilis* utilizes galactose but it is not a PTS substrate. **(I)** Chondroitin sulfate C was not predicted as a PTS substrate.

In addition to confirming glucose as a PTS substrate, we found the *ptsH* mutant had, as expected, greatly diminished growth on N-acetyl-D-glucosamine, N-acetyl-D-galactosamine, and trehalose (Fig 6A–6D, S1 Table). Predicted substrates of sucrose, maltose, and D-cellobiose were unable to support the growth of wild-type HI4320 (Fig 6E–6G), suggesting possible misannotation. D-galactose did facilitate robust growth, but no differences were observed between wild type and mutant (Fig 6H), which is consistent with the prediction that galactose is not a PTS substrate for *P. mirabilis*. Finally, the *ptsH* mutant displayed a growth defect, compared to wild type, in the presence of chondroitin sulfate C (Fig 6I). This is interesting because chondroitin sulfate C is not a predicted PTS substrate; however, chondroitinase activity has been historically described across the *Proteus* genus, including *P. mirabilis* and *P. vulgaris*, resulting in cleavage of the substrate into glucuronic acid and PTS substrate N-acetylgalactosamine [52–55]. More recent work has further shown that clinical *P. mirabilis* isolates can degrade chondroitin sulfate under multiple growth conditions [56,57]. Collectively, we confirmed four predicted PTS substrates and did not identify any additional unpredicted PTS substrates that supported growth of *P. mirabilis* HI4320 (S2 Table).

Remarkably, we detected growth defects of the *ptsH* mutant on various nitrogen sources (S6 Fig). Both ammonia and urea are preferred nitrogen sources that are particularly relevant to the urinary tract environment, and we observed a large growth defect of the *ptsH* mutant, compared to wild type, for both (S6A–S6B Fig). Similarly, L-amino acids such as leucine, arginine, histidine, phenylalanine, and cysteine also resulted in growth defects for the *ptsH* mutant (S6C–S6G Fig). Interestingly, nitrogen sources of serine and methionine did not yield differences between strains (S6H–S6I Fig). The nitrogen-dependent phenotypes we observed correlate with the growth defects of the *ptsH* and *ptsI* mutants in Minimal A with glycerol as the carbon source and provide an explanation why *ptsH* and *ptsI* had more severe defects with glucose as the carbon source compared with *ptsG* or *crr* mutants (Fig 1B–1C). Specifically, glycerol was not expected to be imported via PTS, and reduced *ptsH* and *ptsI* growth in glycerol is likely due to a defect in taking up the nitrogen source (ammonium). Importantly, growth in glycerol was restored when *ptsH* and *ptsI* expression was restored (S1 Fig). We propose the greater reduction of growth for *ptsH* and *ptsI* in glucose compared with *ptsG* or *crr* reflects a cumulative effect of impaired glucose import and defective nitrogen assimilation.

*P. mirabilis* swarming motility is induced by amino acid cues [58,59]. In addition, swarming defects often correlate with fitness defects during experimental UTI [60]. Furthermore, a previous study in *Bacillus cereus* reported swarming defects for a *ptsH* mutant [61]. For these reasons, we tested swarming motility and found that the *ptsH* mutant exhibited a modest but statistically significant reduction in swarming motility compared to wild-type HI4320 (S7A Fig). Because MR/P fimbriation and urease are also crucial for *P. mirabilis* virulence [62], we measured *mrp* promoter invertible element orientation and qualitatively assessed urease activity and observed no notable differences (S7B–S7C Fig).

**Fig 7:**
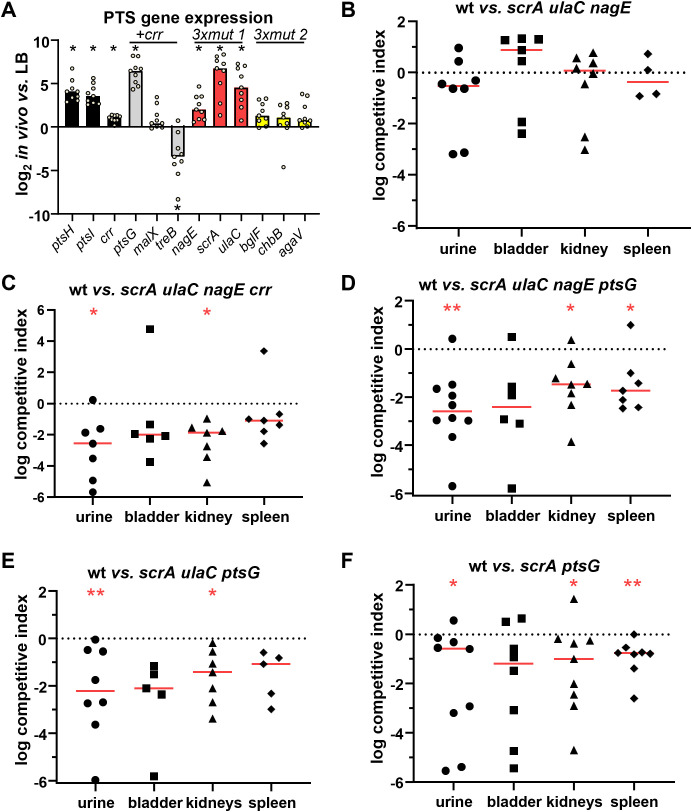
PTS multi-mutant co-challenges. (**A**) Published *in vivo* differential expression was used to prioritize mutant construction [17]. Individual fold-change data points from 9 microarrays are shown, and genes that were significantly differentially regulated in the prior publication are indicated with an asterisk. Red and yellow bars correlate with the arrows in Fig 5. (**B**) a combinatorial triple mutant constructed based on higher induction during experimental UTI (red bars) did not have a competitive disadvantage. (**C**) quadruple mutant with *crr* recapitulated *ptsH* phenotype. (**D**) quadruple mutant with *ptsG* also was similar to *ptsH* phenotype. (**E**) removing *nagE* preserved the *ptsH* phenotype. (**F**) an *scrA ptsG* double mutant had a much weaker defect, emphasizing the combinatorial contribution of different PTS transporters to fitness during experimental UTI. Horizontal lines show medians. Dashed line indicates equal fitness of wild type and mutant (log CI = 0). *P < 0.05; **P < 0.01; one sample Wilcoxon test *vs.* a theoretical median of 0.

To narrow down the specific substrate sugar import pathways that contribute to UTI fitness, we first excluded systems associated with Crr (PMI1830), an Enzyme IIA component not identified as a fitness factor in our In-seq screen (S3, S5 Fig). We then grouped the remaining PTS systems into categories of significantly high (Fig 5, red arrows) or low expression/no (Fig 5, yellow arrows) induction during UTI based on previous *in vivo* transcriptomic data [17] (Fig 7A). A triple mutant targeting the uninduced transporters *bglF*, *chbB*, and *agaV*, with predicted substrates β-glucoside, cellobiose, and N-acetyl-galactosamine, did not exhibit any fitness defect in co-challenge experiments (S8 Fig), suggesting these importers are dispensable in the urinary tract.

We next tested a triple mutant targeting the highly induced genes *nagE*, *scrA*, and *ulaC*, which encode predicted transporters for N-acetylglucosamine, sucrose, and L-ascorbate, respectively. This strain also showed no significant *in vivo* defect (Fig 7B). However, the addition of a fourth mutation in *crr* resulted in a composite fitness defect, with significantly reduced recovery from urine and kidneys (median log_10_ CI -2.55, and -1.87 and *P* = 0.0312 and 0.0156, respectively) (Fig 7C). Of the importers predicted to work with Crr, glucose-specific EIIBC component *ptsG* transcript was the only one induced during experimental UTI in mice (Fig 7A). Replacing *crr* with a *ptsG* mutation produced a similar result, with significant attenuation in urine, kidneys, and spleen (median log_10_ CI -2.60, -1.47, and -1.74, and *P* = 0.0039, 0.0156, and 0.0312, respectively) (Fig 7D). Both quadruple mutants phenocopied the fitness loss seen for the *ptsH* mutant (Fig 2C), indicating that the substrates imported by these systems collectively contribute to *in vivo* fitness. CFU recovery for all PTS mutant co-challenges are shown in S9 Fig.

To determine the minimal functional set of PTS importers needed for urinary tract fitness, we progressively removed individual genes from the *nagE*/*scrA*/*ulaC*/*ptsG* quadruple mutant. After restoring wild type *nagE*, the least induced gene *in vivo* (Fig 7A), the defective phenotype of triple mutant *scrA*/*ulaC*/*ptsG* was not altered (significant median log_10_ CI of -2.22 and -1.42 and *P* = 0.0078 and 0.0156 in the urine and kidneys, respectively) (Fig 7E). However, further removal of the *ulaC* mutation reduced the magnitude of the competitive defect to ≤1 log, although the mutant remained significantly attenuated in urine, kidneys, and spleen (median log_10_ CI -0.59, -1.00, and -0.77, and *P* = 0.0273, 0.0391, and 0.0078, respectively) (Fig 7F). Import mediated by *ulaC*, *scrA*, and *ptsG*, collectively, phenocopies the PTS-dependent *in vivo* defect first observed with the *ptsH* and *ptsI* mutants (Fig 2C–2D). Taken together, this suggests the substrates transported by these three systems all contribute to *P. mirabilis* fitness in the urinary tract and further demonstrates the coordination of the general PTS phosphorelay mediated by PtsH and PtsI with substrate-specific enzyme II PTS importers.

Although we identified *ulaC*, *scrA*, and *ptsG* as important contributors to *in vivo* fitness, only PtsG had an experimentally confirmed substrate in *P. mirabilis* (glucose, Fig 1B). UlaC and ScrA are predicted to import L-ascorbate and sucrose, respectively. However, wild-type HI4320 failed to utilize 10 mM L-ascorbate as a carbon source under aerobic conditions (S10A Fig) and instead showed steadily declining optical density compared to control media lacking a carbon source. *E. coli* has been reported to ferment L-ascorbate under anaerobic conditions [63]. However, an anaerobic atmosphere did not allow *P. mirabilis* growth on ascorbate (S10B Fig), further suggesting that this substrate is not imported by HI4320. While the PMI1775–1777 operon appears to encode a PTS IIA, IIB, IIC importer, closer examination of this locus in other databases did not yield viable alternative substrates. Specifically, TransportDB listed fructose as the substrate, but HI4320 did not grow on D-fructose in the Biolog PM1 carbon source panel (S2 Table).

Likewise, we found that sucrose was not a functional carbon source for strain HI4320 (Fig 6E), raising questions about the annotated role of ScrA. The gene encoding *scrA*, PMI3515, appears to be part of a four-gene operon comprising PMI3514–17 (S10C Fig). The protein encoded by PMI3514 has 47% identity and 67% similarity to *E. coli* MG1655 MurQ, which is an *N*-acetylmuramic acid (MurNAc) 6-phosphate etherase. In *E. coli*, the next gene encodes MurP, a PTS enzyme IICB component that imports MurNAc and shares 53% similarity to HI4320 ScrA. PMI3516 encodes a protein that looks like transcriptional regulator MurR, although in *E. coli*, *murR* is divergently transcribed from the *mur* operon. However, the last genes in each operon (PMI3517 or *yfeW*) are dissimilar. We therefore hypothesized that ScrA imports MurNAc. Despite the similarities, HI4320 was unable to grow using 0.2% MurNAc as a sole carbon source (S10D Fig). Although *E. coli* encodes machinery to import via PTS and shuttle MurNAc into glycolysis and other central metabolic pathways, it is possible that *P. mirabilis* takes up MurNAc via PTS but is unable to utilize this substrate as a sole carbon source. Thus, although both *ulaC* and *scrA* contributed to *P. mirabilis* fitness and are induced during experimental UTI, the substrates for both remain to be determined. These results highlight the need for experimental validation of sugar transporter function in *P. mirabilis*, as many annotations based on *E. coli* homology may not accurately reflect substrate specificity in this organism.

### Glucosuria enhances *P. mirabilis* colonization and increases the severity of infection

The *in vivo* defects observed with *ptsH* and *ptsI* mutants reinforce prior studies demonstrating that carbohydrate metabolism is critical for *P. mirabilis* fitness during urinary tract infection [17,18]. Although sugars are not typically considered a major carbon source in urine, they are clearly accessible to *P. mirabilis* during experimental UTI. We hypothesized that increasing urinary sugar levels would intensify the fitness disadvantage observed for the *ptsH* mutant. We accomplished this using the sodium-glucose cotransporter 2 (SGLT2) inhibitor dapagliflozin, which reduces renal glucose reabsorption leading to increased glucose concentrations in urine [64].

**Fig 8:**
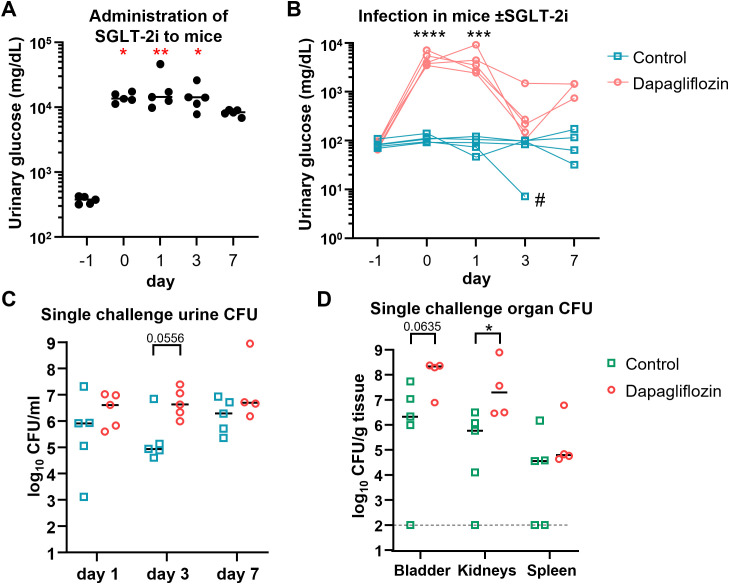
Experimental *P. mirabilis* UTI during SGLT-2i treatment. **(A)** Measurement of urinary glucose. Administration of SGLT-2 inhibitor dapagliflozin to female CBA/J mice via drinking water on “day -1” resulted in increased glucose excretion via urine. *P < 0.05, **P < 0.01 *vs.* day -1, two-way ANOVA with Dunnett’s multiple comparisons test. **B-D,** Experimental inoculation of *P. mirabilis* HI4320 on day zero in mice with or without dapagliflozin treatment (n = 5/group). Of the five mice in the dapagliflozin group, one mouse died at day 6 and one was moribund at day 7 (unable to collect enough urine for glucose measurement). **(B)** Urinary glucose. Lines connect data from individual mice. Glucose levels fell somewhat in infected mice, and the effect was more apparent in the dapagliflozin-treated mice. # indicates one control mouse with undetectable glucose at day 7. ***P < 0.001, ****P < 0.0001 for dapagliflozin-treated mice *vs.* day -1, mixed-effects analysis with Dunnett’s multiple comparisons test. **(C)** Bacterial burden in urine. Median CFU recovery was higher in dapagliflozin-treated mice, although with 5 mice the difference was not statistically significant. **(D)** Bacterial burden in organs at experimental endpoint (7 dpi). Dashed line indicates limit of detection. **C** and **D**, *P < 0.05; for 0.05 < *P* < 0.1, exact value shown; Mann-Whitney test. Horizontal lines denote medians.

As expected, administration of dapagliflozin via drinking water increased glucosuria by more than 20-fold within 24 h in female CBA/J mice, raising the mean glucose from 375 to 14,288 mg/dL, *P* = 0.0225 (Fig 8A). Elevated glucosuria remained for the duration of the 7-day experiment, falling slightly to 8,311 mg/dL by day 7. Following infection with wild-type HI4320, urinary glucose levels remained relatively stable in mice that received normal drinking water over 7 days (Fig 8B). Interestingly, over time, two of the mice inoculated with *P. mirabilis* showed glucose levels that declined by more than half over the course of the experiment; one animal strikingly had an over 90% reduction from baseline by day 3 (77.5 *vs.* 7.21 mg/dL) that further fell below the limit of detection by day 7. Dapagliflozin-treated mice showed increased urinary glucose similar to the pilot study, marked by a stark reduction in urinary glucose on day 3 post-inoculation, suggestive of bacterial glucose consumption (Fig 8B). Additionally, 20% (n = 2) of dapagliflozin-treated animals reached humane endpoints before or at the scheduled termination of the study. This was consistent with higher median bacterial burdens in the urine, particularly on day 3 post-inoculation (Fig 8C). At 7 days post-inoculation, kidney colonization was significantly increased in the dapagliflozin group (Fig 8D, median 1.98 x 10^7^ *vs*. 5.91 x 10^5^ CFU/g, *P* = 0.0397); bladder colonization was also increased, although not statistically significant (median 2.11 x 10^8^ *vs.* 2.15 x 10^6^ CFU/g, *P* = 0.0635, n = 4 in the treated group). The lack of significance is likely due to reduced sample size from increased morbidity and mortality at day 7 post-infection. These results demonstrate that SGLT2 inhibition effectively induces glucosuria in CBA/J mice and promotes enhanced colonization and disease severity during *P. mirabilis* UTI.

**Fig 9:**
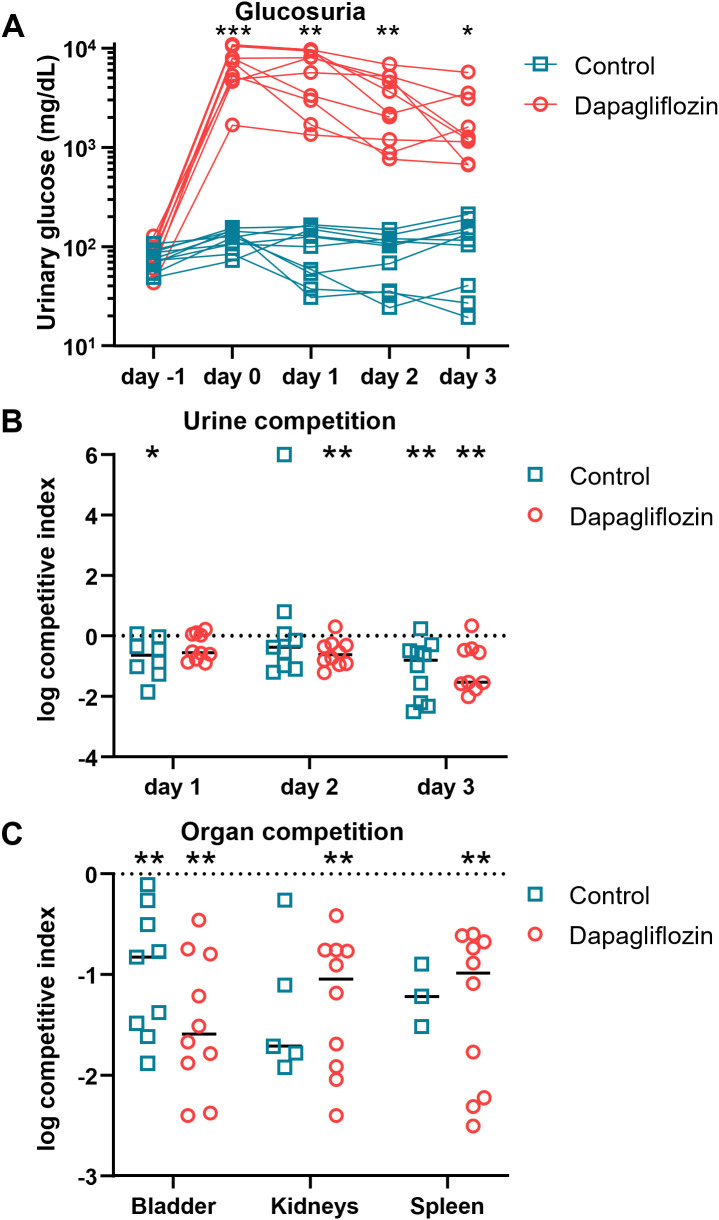
Wild type *vs*. *ptsH* co-challenge during SGLT-2i treatment. Twenty mice, half receiving dapagliflozin and half receiving normal water, were administered a 1:1 mixture of wild type and *ptsH* mutant bacteria. (**A**) urinary glucose. Lines connect urinary glucose levels over time in individual mice. *P < 0.05, **P < 0.01, ***P < 0.001 control *vs.* dapagliflozin, mixed-effects analysis with Šidák’s multiple comparisons test. B-C, competitive indices. Horizontal lines show medians. *P < 0.05, **P < 0.01 *vs.* theoretical median of log CI = 0, one sample Wilcoxon test. Comparisons between control and dapagliflozin groups were not significant (Multiple Mann-Whitney tests with Holm-Šídák correction). (**B**) urinary competitive index measured over time. (**C**) organs at experimental endpoint (3 dpi).

**Fig 10:**
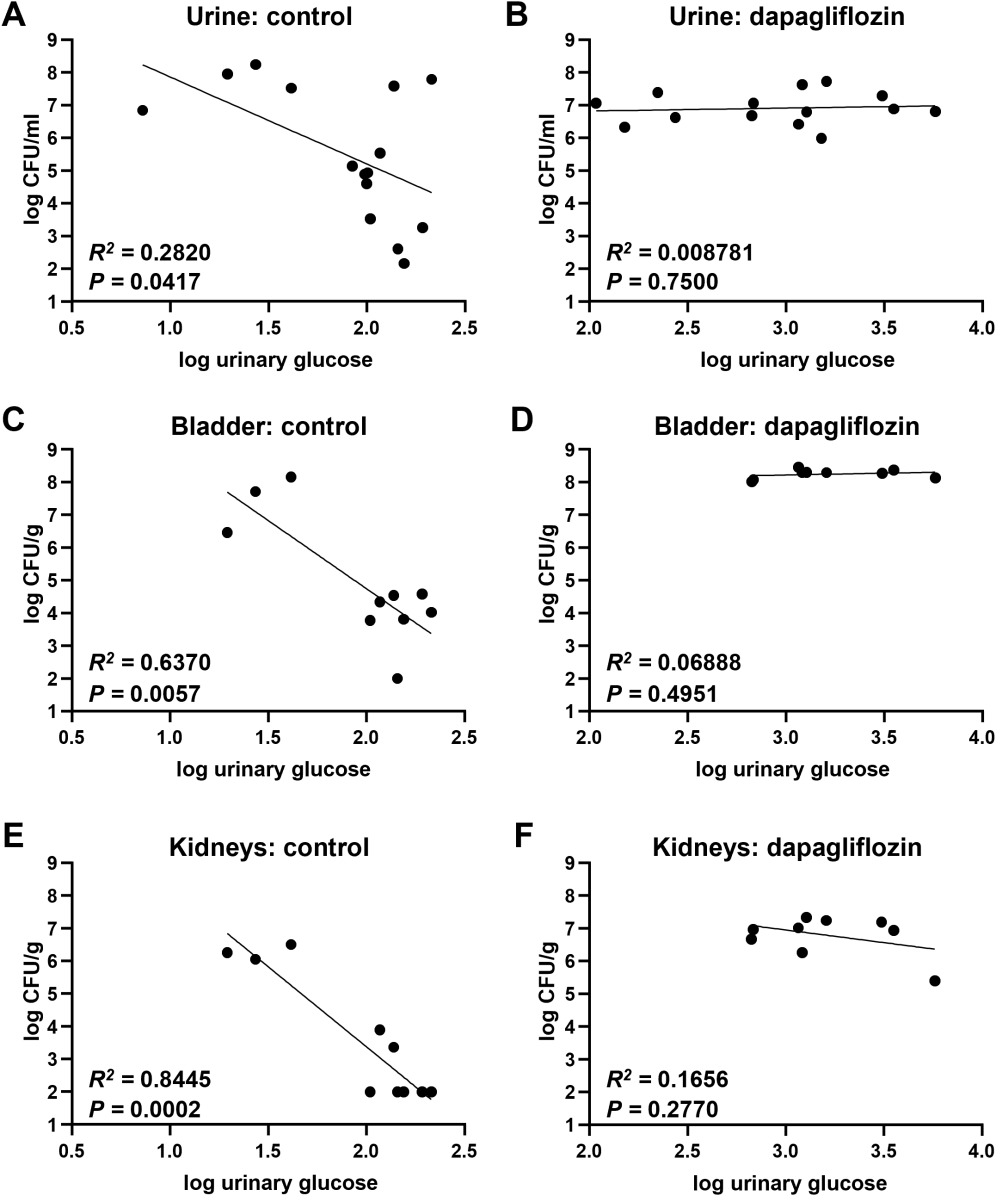
Inverse correlation between glucosuria and CFU recovery (A, C, E) but not during SGLT-2i-induced hyperglucosuria (B, D, F) at 3 dpi. Data were compiled from wild type single challenge and wild type *vs. ptsH* co-challenge (total CFU; limit of detection = 100 for organs). Lines indicate simple linear regression.

Due to the increased morbidity observed during infection of dapagliflozin-treated mice, we next tested the fitness of the *ptsH* mutant in a 3-day co-challenge experiment, timed to coincide with peak glucose utilization and elevated urinary colonization (Fig 8B–8C). As expected, urinary glucose levels remained elevated in dapagliflozin-treated mice and declined by day 3, again suggesting bacterial consumption (Fig 9A). Consistent with previous findings, dapagliflozin-treated mice exhibited higher overall urinary colonization (S11A Fig), and the fitness defect of the *ptsH* mutant was significant on day 2 in treated mice but not in controls (median log_10_ CI -0.64 *vs*. -0.38 control, *P* = 0.0059 and 0.4258, respectively) (Fig 9B). By day 3, the *ptsH* mutant showed a significant fitness defect in the urine of both groups (median log_10_ CI -1.54 *vs*. -0.80 control, *P* = 0.0078 and 0.0039, respectively), and this was also observed in the bladder (-1.59 *vs.* -0.83 control, *P* = 0.0020 and 0.0039, respectively) (Fig 9C). Interestingly, although the fitness defect of the *ptsH* mutant in dapagliflozin-treated mice was not significantly different from control mice in the kidneys (median log_10_ CI -1.04 vs. -1.71 control) and spleens (median log_10_ CI -0.99 vs. -1.22 control), in both organs, the CI reached statistical significance only for dapagliflozin-treated mice. This is because bacteria were recovered from all mice in the dapagliflozin group, whereas many mice in the control group did not have measurable ascending (n = 5; 50%) or disseminated (n = 7; 70%) infection (S11A–S11B Fig). Adding up the total CFU (wild type plus *ptsH*) recovered from each mouse showed that overall bacterial burden was significantly higher in mice treated with dapagliflozin (S11C Fig). When glucose levels were correlated with bacterial burdens on day 3, a strong inverse relationship was observed in control mice across urine, bladder, and kidney samples (R² = 0.28, 0.64, and 0.84, respectively) (Fig 10A, 10C, 10E). No such trend was seen in the dapagliflozin-treated group (Fig 10B, 10D, 10F), suggesting that hyperglucosuria alters the physiological responses of *P. mirabilis* during experimental UTI. These findings support the use of dapagliflozin to model glucosuria in CBA/J mice and demonstrate that elevated urinary glucose exacerbates *P. mirabilis* colonization and infection severity.

### Discussion

The metabolic strategies employed by bacterial pathogens to colonize the urinary tract differ by species, reflecting adaptations to specific host niches. UPEC relies primarily on amino acid metabolism *in vivo*, with carbohydrate utilization playing a minor role [11,18,21]. In contrast, prior transcriptomic studies have shown that *P. mirabilis* upregulates glycolytic enzymes and sugar transporters during UTI, in addition to amino acids, suggesting a greater reliance on sugar-derived carbon sources [17]. Here, we directly assessed the importance of sugar import systems in *P. mirabilis* by generating 47 targeted transporter mutants and evaluating both their *in vitro* growth and *in vivo* fitness. Several mutants displayed *in vivo* fitness defects despite lacking observable phenotypes in standard growth conditions, indicating that specific import systems contribute to pathogenesis in a context-dependent manner.

*In vivo* pooled mutant screens are inherently limited by physiological bottlenecks in the ascending UTI model, including for *P. mirabilis* [45]. Although our In-seq experiments were designed to remain within established bottleneck constraints, we nonetheless observed clear founder effects in a subset of mice, where 1–3 mutants disproportionately dominated sequencing reads from individual organs. Similar stochastic population dynamics have been reported in UPEC infection models, even under carefully controlled conditions [48,65,66]. Despite these limitations, all fitness determinants identified by In-seq were confirmed in a traditional 7-day co-challenge model, exceeding validation rates reported in comparable *E. coli* studies [48]. These findings underscore both the challenges of pooled *in vivo* approaches and the robustness of our experimental design and follow-up strategy.

The Major Facilitator Superfamily (MFS) comprises a large and diverse group of membrane transport proteins characterized by 12 transmembrane helices and driven by electrochemical gradients. MFS transporters are present in both prokaryotes and eukaryotes; for example, glucose uptake in humans is mediated by members of this family [67]. In this study, *xapB*, an MFS transporter, was identified as important for *P. mirabilis* fitness during infection. Although XapB is annotated as a xanthosine permease based on homology to *E. coli* [47], its substrate specificity in *P. mirabilis* remains unverified. Prior studies support a role for nucleoside metabolism in *P. mirabilis* polymicrobial pathogenesis; loss of xanthine-guanine phosphoribosyltransferase (*gpt*) and purine-nucleoside phosphorylase (*deoD*) impaired *in vivo* fitness during co-infection with *Providencia stuartii* [19]. However, we found that strain HI4320 could not use xanthosine as a sole carbon or nitrogen source under the conditions we tested, and guanosine uptake occurred independently of XapB, validated in a *guaA/xapB* double mutant. *P. mirabilis* HI4320 encodes nine additional genes that are annotated as nucleoside importers, suggesting there are multiple routes of entry for these molecules.

Importantly, the *xapB* mutant displayed a significant defect during experimental UTI, suggesting that the imported substrate(s) is likely non-redundant. In this study, we did not initially set out to study nucleoside import; *xapB* was included in our 47 mutant panel because it was annotated as a generic sugar importer by TransportDB at the start of the project. Interestingly, over the course of this work, TransportDB updated the annotation for *xapB* at least twice, first to generic nucleoside transport, and then to melibiose importer *melB.* Melibiose is a plant disaccharide that did not support growth of HI4320 in our carbon source testing, leading us to conclude that this revised annotation is also likely incorrect. These findings highlight a recurring challenge in microbiological research: reliance on gene annotations inferred from *E. coli* often fail to recapitulate actual function.

Similar annotation discrepancies were observed for multiple phosphotransferase system (PTS) family transporters in our study. PtsH and PtsI are conserved upstream components of the PTS, a multi-component phosphorelay essential for sugar uptake and carbon catabolite repression [27]. Both *ptsH* and *ptsI* mutants exhibited reproducible defects in a murine model of ascending UTI, consistent with a central role for glycolysis in *P. mirabilis* pathogenesis and contrasting with UPEC and other uropathogens [17,18,68]. Even so, because PtsH and PtsI interact with many sugar-specific components, these findings did not identify the specific substrates responsible for fitness.

Here, we found that the three most-induced substrate-specific PTS genes during experimental *P. mirabilis* UTI in mice, *ptsG*, *scrA*, and *ulaC*, combined to recapitulate the *ptsH* mutant phenotype. However, *in vitro* substrate validation for two of these transporters was inconsistent with predicted annotations. HI4320 was unable to use either sucrose, predicted to be imported by ScrA, or L-ascorbate, predicted to be imported by UlaC, as a carbon source. Nor did ScrA support growth on MurNAc in follow-up experiments despite homology with the *E. coli mur* locus. Likewise, HI4320 failed to grow on predicted PTS substrates maltose, cellobiose, and β-glucosides. Incidentally, although *E. coli* was initially shown to utilize cellobiose, later work indicated the more physiologically likely substrate is chitobiose [69]. Because the *chbB* mutant was not found to be important for *P. mirabilis* fitness during experimental UTI (S8 Fig), we did not investigate chitobiose further. Overall, only 4/9 of the KEGG-predicted PTS substrates for *P. mirabilis* verified experimentally. Notably, our findings are consistent with Biolog data for *P. mirabilis* isolates obtained from broiler chickens [70]. It is possible that imported sugars or sugar derivatives are used by *P. mirabilis* but are not connected to central metabolism in a way that would allow use as a sole carbon or nitrogen source. However, most PTS substrates would typically be accessible to metabolic pathways such as glycolysis and other sugar interconversion pathways, and carbohydrate substrate prediction is, at the current time, frequently unreliable. We therefore conclude that the most likely explanation for the mismatch between PTS substrate prediction and experimental growth results is misannotation. Teasing apart the combinatorial contributions of UlaC, ScrA, and PtsG to UTI fitness will be greatly aided by identifying the substrates for the first two transporters.

Beyond sugar transport, the PtsHI phosphorelay appears to influence nitrogen metabolism. The *ptsH* mutant showed defects when grown on ammonia, urea, and several amino acids, suggesting cross-regulation between carbon and nitrogen pathways. Likewise, both *ptsH* and *ptsI* mutants had an initially unexpected decrease in growth on glycerol and displayed a more pronounced defect on glucose compared with the more substrate-specific *crr* and *ptsG* mutants. In *E. coli*, the PTS^Ntr^ system coordinates nitrogen assimilation by sensing glutamine and α-ketoglutarate [31,71], but has not been characterized in *P. mirabilis*. Our findings raise the likelihood of similar regulatory integration.

While the urinary tract is generally sugar-poor, elevated glucose levels occur in specific conditions such as diabetes or treatment with SGLT2 inhibitors. Diabetic individuals have increased UTI susceptibility, and glucosuria is common in both humans and mouse models [68,72]. SGLT2 inhibitors, such as dapagliflozin, reduce renal glucose reabsorption, leading to elevated urinary glucose. Although debated in clinical studies [73,74], preclinical studies consistently show worsened UTI outcomes under glucosuric conditions, including increased bacterial burden and dissemination [25,75]. We observed similar results for *P. mirabilis* infection, where dapagliflozin treatment led to elevated colonization and exacerbated disease. Despite these findings, the *ptsH* mutant did not demonstrate an enhanced competitive defect in urine under glucosuric conditions, contrary to our prediction. One explanation for this result is that additional sugar import systems contribute to fitness during UTI, thereby partially masking the effect of *ptsH* loss under glucosuric conditions. Consistent with this idea, growth of the *ptsH* mutant in minimal medium with glucose was slowed but not abolished, indicating that glucose can still enter the cell through alternative pathways. Several transporters, including *crr*, exhibited borderline effects on *in vivo* fitness in the In-seq screen, suggesting that their contributions may become apparent only in specific metabolic contexts. It is also possible that loss of *ptsH* induces compensatory metabolic or regulatory responses that mitigate fitness defects when glucose availability is increased.

The connection between glucosuria and UTI severity likely involves more than nutrient availability. Hyperglycemia impairs innate immunity, including reduced neutrophil responses and cytokine signaling in the kidney [25,76]. We observed an inverse correlation between urinary glucose and bacterial burden in untreated mice, but this trend was lost in dapagliflozin-treated animals, likely reflecting both increased glucose consumption and altered host-pathogen interactions. Mouse background also matters; others have reported that C57BL/6J mice treated with dapagliflozin had similar bladder and urine colonization by UPEC [26]. C3H/HeOuJ mice had to be administered 10 mg/kg of dapagliflozin to see increased UPEC burden in bladder and kidneys, and results were dependent on bacterial strain [26]. Mice genetically engineered to develop type 2 diabetes (*db*/*db*) also have increased UTI risk that is correlated with altered innate immune markers [77]. However, dapagliflozin-treated CBA/J mice were shown to have increased UPEC and *K. pneumoniae* colonization at chronic and acute timepoints [25]. Interestingly, while UPEC infections in this model could be sustained for 7 days, *P. mirabilis* infection resulted in increased morbidity at this time point, consistent with this species’ increased contribution to UTI in complicated backgrounds. Last, streptozotocin-treated ICR mice, a model of type 1 diabetes, carry higher *P. mirabilis* burden in bladders and kidneys, and glucose has been found to increase *P. mirabilis* adherence to cultured kidney cells [78]. Further investigation into *P. mirabilis* pathogenesis in diabetic models is warranted.

In conclusion, our findings provide mechanistic insight into the metabolic requirements of *P. mirabilis* during UTI. Sugar import systems, particularly PTS transporters, are essential for *in vivo* fitness, and substrate annotations based on *E. coli* must be experimentally validated. Our data reveal regulatory crosstalk between carbon and nitrogen metabolism and demonstrate that host metabolic conditions, such as glucosuria, exacerbate infection severity. These results have implications for managing UTI in individuals with metabolic disease and highlight the need for continued investigation into the roles of uncharacterized transporters and their regulation in *P. mirabilis* pathogenesis. They also underscore a broader gap in our understanding of the *P. mirabilis* genome and the limitations of relying on comparative functional annotation. Future work will focus on identifying the specific substrates imported by fitness-contributing transporters, confirming the presence of these metabolites in the urinary tract, and determining how *P. mirabilis* accesses these nutrient sources during infection.

## Methods

### Ethics statement

Animal experiments were approved by the University of Michigan Medical School Institutional Animal Care and Use Committee, protocol number PRO00010856. During catheterization procedures, mice were anesthetized by intraperitoneal injection of ketamine/xylazine. Mice were euthanized by inhalant isoflurane anesthetic overdose prior to organ removal.

### Bacterial strains and culture conditions

*P. mirabilis* strain HI4320 was isolated from the urine of an elderly female nursing home patient with a long-term (≥30 days) indwelling catheter [3,40,79]. *E. coli* TOP10 (Thermo Fisher) was used for plasmid construction and maintenance. Bacteria were routinely cultured at 37°C in lysogeny broth (LB; per liter: 10 g tryptone, 5 g yeast extract, 0.5 g NaCl) with aeration or on LB solidified with 1.5% agar. As needed, antibiotic selection was used as follows (µg/mL): kanamycin 25, ampicillin 50, chloramphenicol 20. For experiments requiring minimal, chemically defined media, Minimal A [MinA; per liter: 10.5 g of K_2_HPO_4_, 4.5 g of KH_2_PO_4_, 0.47 g of sodium citrate, 1.0 g of (NH_4_)_2_SO_4_; autoclave to sterilize and add 1 mL of 1 M MgSO_4_, 10 mL of 20% glycerol (or other carbon source as specified), and 1 mL of 1% nicotinic acid] was used [80]. The carbon source in Minimal A was 0.2% glycerol unless otherwise specified. All strains used in this study are listed in S3 Table.

### Mutant construction

All mutants were constructed using a *P. mirabilis*-tailored version of targetron insertional mutagenesis [42,81]. Briefly, stable chromosomal mutations were constructed using a synthesized 353 bp group II intron fragment (eBlocks, Integrated DNA Technologies) that specifically targeted each gene designed using the ClosTron prediction algorithm [82]. Reprogrammed intron fragments were cloned into pACD4K-CloxP using NEBuilder HiFi DNA Assembly master mix (New England Biolabs) with primers designed to amplify vector or intron templates and confirmed by DNA sequencing (Eurofins). Targetron-containing plasmids and a source for T7 polymerase, pAR1219 [83], were introduced into *P. mirabilis* HI4320 using electroporation and induced to jump into the specified genes. Insertional mutations in kanamycin-resistant mutants were confirmed using PCR. To construct multiple mutations in the same background, the kanamycin resistance gene in the initial insertion was removed using *cre*/lox recombination to create a markerless mutant [42,84,85]. Targetron sequences are listed in S4 Table.

### Murine model of ascending UTI

Bacterial fitness during UTI was assessed using a well-established mouse model [50,86,87]. Briefly, overnight cultures of *P. mirabilis* were diluted in LB to OD_600_ = 0.092-0.094 (~2 × 10^8^ CFU/mL). For co-challenge experiments, wild type and mutant bacteria were mixed 1:1. Ten female CBA/J mice, aged 5–6 weeks (Jackson Laboratory), were transurethrally inoculated with 50 µL of this 1:1 mixture (10^7^ CFU/mouse) over 30 s using a Harvard pump. At 7 days post-inoculation, urine was collected; mice were euthanized; and bladders, kidneys, and spleens were harvested. Organs were homogenized and plated to quantify CFU; mutants were distinguished from wild-type colonies using kanamycin. Competitive indices were calculated for each site by comparing the ratio of output wild type and mutant to the ratio of input bacteria [50,88]. For sites with no recovered CFU, the limit of detection for urine was set to 20 and for organs 100. Statistical significance of competitive indices was calculated using the Wilcoxon signed rank test.

### Pooled transporter mutant murine challenge

To measure the relative contributions of sugar transporters to UTI, 47 targetron mutants were individually cultured and the density adjusted as described above. 23 (ABC + MFS, group 1) or 24 (PTS + Others, group 2) strains were mixed together in equal volume, and 15 mice/group were transurethrally inoculated with 50 µL (~10^7^) CFU of either mixture. A 1 mL aliquot of each input was directly pelleted for DNA purification and a second aliquot was plated to quantify CFU (group 1, 2.51 × 10^8^ CFU/mL; group 2, 2.68 × 10^8^ CFU/mL). After 24 h, urine was collected and diluted to 250 µL, mice were euthanized, and bladders and kidneys were collected. Organs were homogenized (Omni International) in 2 mL phosphate-buffered saline (PBS); a portion of organs and urine was dilution-plated to determine output CFU, and the remainder was spread-plated on LB agar containing kanamycin. The next day, colonies from each plate were swabbed into 10 mL of PBS, pelleted, and frozen. Colonies from the plated input samples were also collected as a control for growth on agar (input spiral, insp). Chromosomal DNA was purified from inputs and urine, bladder, and kidney outputs using the Wizard Genomic DNA Purification Kit (Promega) and quantified using a Qubit fluorometer (Invitrogen).

### Insertional site sequencing (In-seq)

Targetron junctions were enriched and prepared for high-throughput sequencing with modification of established protocols [89,90]. Briefly, we used the NEBNext Ultra II FS DNA Library Prep with Sample Purification Beads kit to fragment DNA into 200–450 bp lengths and ligate sequencing adaptors with sequences modified to be in line with lower %GC content in *P. mirabilis* (TA_Adaptor_Top and TA_Adaptor_Bottom). Targetron-gene junctions were enriched by PCR using primers Targetron_enrich_For and Transposon_enrich_Rev (S5 Table). Samples (n = 78) were barcoded by indexing PCR to label each specific library using NEBNext Multiplex Oligos for Illumina (Dual Index Primers Set 1) (S1 Table). PCR products were measured by Qubit and submitted to the University of Michigan Advanced Genomics Core for Illumina sequencing (150 nt, paired-end).

### In-seq analysis

Mutant fitness in mice was assessed by quantifying the proportion of each of the 47 targetron-gene junctions in the input and output sequences. Identification and quantification of targetron junctions was conducted by the University of Michigan Medical School’s Bioinformatics Core. Filtering, trimming, and deduplication of reads was accomplished using an established Tn-seq pipeline [19,91], and subsequent steps were adapted for the relatively small number of targetron insertions. BLASTN with the 3′ end of the targetron as query was used to identify targetron-containing sequences consisting of at least 100 nt at 100% identity on the plus strand. The 19 nt immediately following the targetron sequence were used to uniquely identify the locus of gene insertion for each targetron (*i.e.*, 47 input mutants). A counts matrix was generated from the 1,739,134 unique 19 nt reads. Features which were members of each individual group (1 or 2) were input into EdgeR, a limma-based R package which is able to deal with group sizes of only one sample in a differential enrichment calculation [92]. Deduplicated library sizes were used for depth normalization.

### Genetic complementation

To complement growth defects for PTS mutants, genes were complemented *in trans* using the pGEN vector backbone [93] and expressed under their native promoters as predicted using the BPROM module in SoftBerry [94]. Specifically, *ptsH*, *ptsI*, *ptsHI* together, and *crr* were all expressed from the *ptsH* predicted promoter, while *ptsG* was expressed from its monocistronic promoter. Genes and promoters were PCR-amplified from *P. mirabilis* HI4320 genomic DNA and cloned using the Gibson method (NEBuilder, New England Biolabs). Clones were constructed in *E. coli* TOP10 cells and first screened by PCR then confirmed by DNA sequencing. Plasmids were then introduced to relevant *P. mirabilis* strains using electroporation and selection on ampicillin. Primers used for cloning are listed in S5 Table and complementation plasmids are shown in S6 Table.

### Diabetic UTI murine model

To assess *P. mirabilis* UTI during glucosuria, we adapted the method from Nishitani *et al.* [95]. The SGLT-2 inhibitor dapagliflozin (MedChem Express) was dissolved in ethanol (125 mg/ml), then diluted in Ann Arbor city water to a final concentration of 0.02 mg/ml. Female CBA/J mice were administered dapagliflozin via drinking water *ad libitum* beginning 24 h before bacterial inoculation. Bacteria were prepared as described above. Urine was collected at specified intervals and glucose was quantified using an Infinity glucose hexokinase assay (Thermo Fisher) according to the manufacturer’s instructions. Aliquots of urine from the same time points were diluted and plated to quantify CFU, and bacterial burden in organs was assessed as described above.

### Growth curves

Overnight cultures were started from a single colony in lysogeny broth incubated at 37°C with aeration. For downstream assays, cultures were diluted 1:100 (v/v) into sterile media. Bacterial growth over time (24 h) was measured in triplicate by recording the optical density at 600 nm (OD_600_) at 15 min intervals using a Bioscreen C set to 37°C with continuous shaking. Growth curves with genetically complemented mutants were performed without ampicillin, as pGEN is stable without selection [93]. Chemical complementation of growth defects was performed in Minimal A medium with 0.2% glycerol as the carbon source unless otherwise specified. Anaerobic growth curves were conducted in an anaerobic chamber (Coy Lab Products, Grass Lake, MI) at 37°C under an atmosphere of 5% H_2_, 5% CO_2_, and 90% N_2_. OD_600_ of 96-well plates was measured every 10 mins for 48 h in a microplate stacking device (BioStack 2WR) with coupled absorbance reader (Powerwave HT, BioTek Instruments). Doubling times for the 47 transporter mutants were calculated from three biological replicates using the AMiGA software package in [R] [96] from 0.5 to 12 hours. Doubling time was computed as ln [2] multiplied by the inverse of the maximum specific growth rate. To identify potential sugar transporter substrates, Biolog Phenotype MicroArray plates PM1–2 (carbon sources) and PM3 (nitrogen sources) were inoculated with wild-type or mutant *P. mirabilis* and assayed for growth every 10 min for 24 h using a LogPhase 600 Microbiology Reader (BioTek) with shaking. Biolog cultures were conducted using the manufacturer’s recommended medium with the respiration substrate (tetrazolium-based dye) omitted.

### Swarming motility

Swarming motility experiments were conducted as previously described [97]. Briefly, 5 µl of a logarithmic-phase culture was added to the center of an LB (10 g/L NaCl) agar plate, allowed to dry, and incubated at 30°C for 16 h, after which the swarming radius was measured.

### Invertible element assay

Orientation of the *mrp* promoter invertible element (IE) was measured as previously described [98]. Briefly, strains were cultured in LB to log phase with aeration (a naturally IE-OFF condition) or statically for 48h (a naturally majority-ON condition). Bacteria were adjusted to OD_600_ = 0.4, incubated at 95°C for 10 min, then subjected to PCR using primers Pm IE P1 and Pm IE P2 (S5 Table). Amplicons were digested with AflII (NEB) and electrophoretically separated on a 2% agarose gel. The proportion of ON and OFF IE orientation was assessed using densitometry (Bio-Rad Image Lab v6.1.0).

### Urease test

Christensen’s urea agar, with tryptone substituted for peptone, was used to qualitatively assess urease activity [99]; per liter: 1g tryptone, 1g dextrose, 5g NaCl, 2g KH_2_PO_4_, 20g urea, 0.012g phenol red, 15g agar. The first six ingredients were filter-sterilized then added to autoclaved agar before dispensing into slants. A 10 µl loop of overnight LB culture was added to each slant, which was then incubated at 37°C and observed from 0.5-24 h for a color change from yellow to pink, indicating the release of ammonia and subsequent rise in pH.

### Statistical analysis

All graphs were plotted, and in most cases, statistics calculated using GraphPad Prism 10. *P* values for In-seq data were generated using edgeR’s exactTest function [100]. Statistical tests and significance values used for each experiment are indicated in figure legends. Error bars show SD unless otherwise indicated.

## Supporting information

S1 Table Barcodes used for Illumina sequencing. (XLSX)

S2 Table PTS Biolog data and KEGG predictions. (XLSX)

S3 Table Bacterial strains used in this study. (XLSX)

S4 Table Targetron intron sequences used to generate 47 transporter mutants. (XLSX)

S5 Table Primers used in this study. (XLSX)

S6 Table Plasmids used in this study. (XLSX)

S1 Fig Complementation of glucose-dependent growth defects. Mutants with glucose-dependent growth defects were genetically complemented using an empty vector (pGEN), the mutated gene under control of its native promoter (either *ptsH* or *ptsG* promoters; pGEN-gene name), or, if relevant, the mutated gene and downstream genes within an operon *(ptsHI*). Bacteria were cultured in Minimal A with 0.2% glucose as the carbon source. Each experiment was conducted in technical triplicate with three biological replicates. Error bars show SD.
(TIF)

S2 Fig Group 1 (ABC and MFS) mutant recovery from mice, 24 h post-infection. (A) urine; (B) bladder; (C) kidneys. Each data point represents sequencing reads obtained from one mouse. *P* values were generated using edgeR’s exactTest function. *P < 0.05. Exact *P* values shown when 0.1 > *P* > 0.05.
(TIF)

S3 Fig Group 2 (PTS and Others) mutant recovery from mice, 24 h post-infection. (A) urine; (B) bladder; (C) kidneys. PMI1776 is missing because PMI1176 was inadvertently inoculated instead. Each data point represents sequencing reads obtained from one mouse. *P* values were generated using edgeR’s exactTest function. *P < 0.05. Exact *P* values shown when 0.1 > *P* > 0.05.
(TIF)

S4 Fig Wild type and mutant CFU recovered from 7 d murine 1:1 co-challenges shown in Fig. 2. (A) Wild type *vs.* PMI1570 *xapB* (n = 10). (B) Wild type *vs.* PMI1828 *ptsH* (n = 15). C, Wild type *vs.* PMI1829 *ptsI*. Dashed lines indicate limit of detection (urine, 20 CFU; organs, 100 CFU). *P < 0.05; **P < 0.01; exact values shown for 0.1 > *P* > 0.05, Mann-Whitney U test. Horizontal lines denote medians.
(TIF)

S5 Fig Testing guanosine as a nutrient source. A-B, Biolog plate PM3 suggested differential growth of wild type and PMI1570 *xapB* on guanosine as sole nitrogen source. Two independent replicates are separately shown to highlight the extreme variability of erratic increase in OD_600_ for wild type but not *xapB.* C-D, Growth in Minimal A containing 0.2% glycerol. (C) Titration of guanosine (G) to restore growth of the *guaA* mutant (n = 1–3). (D) 0.05 mg/mL guanosine caused slower growth and the effect was not due to the DMSO solvent (n = 3). Error bars show SD.
(TIF)

S6 Fig Comparison of wild type *vs. ptsH* growth on selected nitrogen sources (n = 2; error bars = SD). A-B, growth defect for *ptsH* mutant using preferred nitrogen sources. C-G, growth defects for *ptsH* mutant on selected amino acid nitrogen sources. H-I, not all amino acids produced reduced growth for the *ptsH* mutant.
(TIF)

S7 Fig Virulence-associated phenotypes of *ptsH*, *ptsI*, and *xapB.* (A) Swarming motility. PMI1828 *ptsH* mutant had a modest but significant decrease in swarming motility. (B) Invertible element (IE) assays show the orientation of the *mrp* promoter IE (ON or OFF). Culturing statically for 48h increases ON while the IE mostly remains OFF in logarithmic aerated culture. For both A and B, n = 3; error bars show SD. *P < 0.05, ns not significant, one-way ANOVA *vs.* wild type with Dunnett’s multiple comparisons test. (C) Urease activity is indicated by basic pH (pink) on urea agar, shown here at 3 h and 24 h post-inoculation. Uropathogenic *E. coli* CFT073, which is urease-negative, was included as a control. Representative experiment is shown (n = 2).
(TIF)

S8 Fig Triple mutant co-challenge of *in vivo* uninduced PTS transporters. A triple mutant with the least-induced *in vivo* PTS transporter genes had no defect in co-challenge competition with wild type. Horizontal lines show medians. Dashed line indicates equal fitness of wild type and mutant (log CI = 0).
(TIF)

S9 Fig PTS multi-mutant co-challenge CFU data. A-F, Wild type and mutant CFU recovered from 7 d murine 1:1 co-challenges shown in Fig. 7. Solid circles are wild type (wt) and open circles are mutant (n = 10 per co-challenge). Dashed lines indicate limit of detection (urine, 20 CFU; organs, 100 CFU). *P < 0.05; **P < 0.01; exact values shown for 0.1 > *P* > 0.05, Mann-Whitney U test. Horizontal lines denote medians.
(TIF)

S10 Fig Substrates remain unconfirmed for both UlaC and ScrA. A-B, ascorbate growth curves with wild-type *P. mirabilis* HI4320. (A) aerobic atmosphere (n = 1). (B) anaerobic atmosphere (n = 2). In B, glucose was used as the carbon source for the positive control. C-D, further investigation of the PMI3515 (*scrA*) locus and substrate. (C) Organization of PMI3515 transporter locus compared with *E. coli mur* locus. Colors indicate genes encoding proteins with similar functions, and numbers above genes indicate % identity/similarity with the same-colored predicted protein. (D) Growth curves in Minimal A. Using MurNAc as the sole carbon source did not allow growth by wild-type HI4320 (n = 2). Error bars = SD.
(TIF)

S11 Fig Bacterial recovery from mice co-challenged with 1:1 wild type *vs. ptsH* mutant. Mice were either treated with dapagliflozin or received normal water (control). Horizontal lines indicate medians. (A) urine CFU at days 1, 2, or 3 post-inoculation. (B) bacterial recovery from tissues 3 d post-inoculation. A-B, *P_ad_j = below threshold, multiple Wilcoxon tests with Holm-Šídák correction. (C) Total CFU (wt + *ptsH*) shows higher overall colonization during hyperglucosuria. *P_ad_j = below threshold, multiple Mann-Whitney tests with Holm-Šídák correction. A-C, Dashed lines indicate limit of detection (urine = 20; organs = 100).
(TIF)

S1 Dataset Group 1 In-seq EdgeR outputs. Sequencing reads containing the end of the targetron insertion were aligned with the *P. mirabilis* HI4320 genome and analyzed for relative mutant recovery using EdgeR. The spreadsheet tabs are 1) overall statistics for each mutant in Group 1; 2) urine *vs.* input; 3) kidney *vs.* input; 4) bladder *vs.* input; 5) input spiral (control for outgrowth on agar) *vs.* input; and 6) list of abbreviations and nomenclature.
(XLSX)

S2 Dataset Group 2 In-seq EdgeR outputs. Sequencing reads containing the end of the targetron insertion were aligned with the *P. mirabilis* HI4320 genome and analyzed for relative mutant recovery using EdgeR. The spreadsheet tabs are 1) overall statistics for each mutant in Group 2; 2) urine *vs.* input; 3) kidney *vs.* input; 4) bladder *vs.* input; 5) input spiral (control for outgrowth on agar) *vs.* input; and 6) list of abbreviations and nomenclature.
(XLSX)

S3 Dataset Biolog growth curve data. OD_600_ measurements from 0-24h are shown for wild-type *P. mirabilis* HI4320 as well as *ptsH* and *xapB* mutants cultured in Phenotype MicroArray plates PM1, PM2, and PM3 (Biolog).
(XLSX)

## References

[1] Foxman B. Urinary tract infection syndromes: occurrence, recurrence, bacteriology, risk factors, and disease burden. Infect Dis Clin North Am. 2014;28(1):1–13. doi: 10.1016/j.idc.2013.09.003 24484571

[2] Foxman B. Epidemiology of urinary tract infections: incidence, morbidity, and economic costs. Dis Mon. 2003;49(2):53–70. doi: 10.1067/mda.2003.7 12601337

[3] Warren JW, Tenney JH, Hoopes JM, Muncie HL, Anthony WC. A prospective microbiologic study of bacteriuria in patients with chronic indwelling urethral catheters. J Infect Dis. 1982;146(6):719–23. doi: 10.1093/infdis/146.6.719 6815281

[4] Armbruster CE, Prenovost K, Mobley HLT, Mody L. How Often Do Clinically Diagnosed Catheter-Associated Urinary Tract Infections in Nursing Homes Meet Standardized Criteria? J Am Geriatr Soc. 2017;65(2):395–401. doi: 10.1111/jgs.14533 27858954 PMC5310979

[5] Griffith DP, Musher DM, Itin C. Urease. The primary cause of infection-induced urinary stones. Invest Urol. 1976;13(5):346–50. 815197

[6] Johnson DE, Russell RG, Lockatell CV, Zulty JC, Warren JW, Mobley HL. Contribution of Proteus mirabilis urease to persistence, urolithiasis, and acute pyelonephritis in a mouse model of ascending urinary tract infection. Infect Immun. 1993;61(7):2748–54. doi: 10.1128/iai.61.7.2748-2754.1993 8514376 PMC280917

[7] Mobley HL, Warren JW. Urease-positive bacteriuria and obstruction of long-term urinary catheters. J Clin Microbiol. 1987;25(11):2216–7. doi: 10.1128/jcm.25.11.2216-2217.1987 3320089 PMC269446

[8] Stickler DJ. Clinical complications of urinary catheters caused by crystalline biofilms: something needs to be done. J Intern Med. 2014;276(2):120–9. doi: 10.1111/joim.12220 24635559

[9] Brückner H, Schieber A. Determination of amino acid enantiomers in human urine and blood serum by gas chromatography-mass spectrometry. Biomed Chromatogr. 2001;15(3):166–72. doi: 10.1002/bmc.57 11391672

[10] Bender DA. Amino Acid Metabolism. 3 ed. Chichester, West Sussex, UK: Wiley-Blackwell; 2012. 480 p.

[11] Sintsova A, Frick-Cheng AE, Smith S, Pirani A, Subashchandrabose S, Snitkin ES, et al. Genetically diverse uropathogenic Escherichia coli adopt a common transcriptional program in patients with UTIs. Elife. 2019;8:e49748. doi: 10.7554/eLife.49748 31633483 PMC6802966

[12] Shea AE, Forsyth VS, Stocki JA, Mitchell TJ, Frick-Cheng AE, Smith SN, et al. Emerging roles for ABC transporters as virulence factors in uropathogenic Escherichia coli. Proc Natl Acad Sci U S A. 2024;121(16):e2310693121. doi: 10.1073/pnas.2310693121 38607934 PMC11032443

[13] Conway T, Cohen PS. Commensal and Pathogenic *Escherichia coli* Metabolism in the Gut. Microbiol Spectr. 2015;3(3). doi: 10.1128/microbiolspec.MBP-0006-2014 26185077 PMC4510460

[14] Carbohydrates in the urine. JAMA. 1933;100(23):1867. doi: 10.1001/jama.1933.02740230045014

[15] Stryeck S, Horvath A, Leber B, Stadlbauer V, Madl T. NMR spectroscopy enables simultaneous quantification of carbohydrates for diagnosis of intestinal and gastric permeability. Sci Rep. 2018;8(1):14650. doi: 10.1038/s41598-018-33104-8 30279548 PMC6168465

[16] Mack CI, Weinert CH, Egert B, Ferrario PG, Bub A, Hoffmann I, et al. The complex human urinary sugar profile: determinants revealed in the cross-sectional KarMeN study. Am J Clin Nutr. 2018;108(3):502–16. doi: 10.1093/ajcn/nqy131 30535088 PMC6134285

[17] Pearson MM, Yep A, Smith SN, Mobley HLT. Transcriptome of Proteus mirabilis in the murine urinary tract: virulence and nitrogen assimilation gene expression. Infect Immun. 2011;79(7):2619–31. doi: 10.1128/IAI.05152-11 21505083 PMC3191972

[18] Alteri CJ, Himpsl SD, Mobley HLT. Preferential use of central metabolism in vivo reveals a nutritional basis for polymicrobial infection. PLoS Pathog. 2015;11(1):e1004601. doi: 10.1371/journal.ppat.1004601 25568946 PMC4287612

[19] Armbruster CE, Forsyth-DeOrnellas V, Johnson AO, Smith SN, Zhao L, Wu W, et al. Genome-wide transposon mutagenesis of Proteus mirabilis: Essential genes, fitness factors for catheter-associated urinary tract infection, and the impact of polymicrobial infection on fitness requirements. PLoS Pathog. 2017;13(6):e1006434. doi: 10.1371/journal.ppat.1006434 28614382 PMC5484520

[20] Alteri CJ, Smith SN, Mobley HLT. Fitness of Escherichia coli during urinary tract infection requires gluconeogenesis and the TCA cycle. PLoS Pathog. 2009;5(5):e1000448. doi: 10.1371/journal.ppat.1000448 19478872 PMC2680622

[21] Snyder JA, Haugen BJ, Buckles EL, Lockatell CV, Johnson DE, Donnenberg MS, et al. Transcriptome of uropathogenic Escherichia coli during urinary tract infection. Infect Immun. 2004;72(11):6373–81. doi: 10.1128/IAI.72.11.6373-6381.2004 15501767 PMC523057

[22] Fu AZ, Iglay K, Qiu Y, Engel S, Shankar R, Brodovicz K. Risk characterization for urinary tract infections in subjects with newly diagnosed type 2 diabetes. J Diabetes Complications. 2014;28(6):805–10. doi: 10.1016/j.jdiacomp.2014.06.009 25161100

[23] Confederat L-G, Condurache M-I, Alexa R-E, Dragostin O-M. Particularities of Urinary Tract Infections in Diabetic Patients: A Concise Review. Medicina (Kaunas). 2023;59(10):1747. doi: 10.3390/medicina59101747 37893465 PMC10608443

[24] van der Aart-van der Beek AB, de Boer RA, Heerspink HJL. Kidney and heart failure outcomes associated with SGLT2 inhibitor use. Nat Rev Nephrol. 2022;18(5):294–306. doi: 10.1038/s41581-022-00535-6 35145275

[25] Saenkham P, Jennings-Gee J, Hanson B, Kock ND, Adams LG, Subashchandrabose S. Hyperglucosuria induced by dapagliflozin augments bacterial colonization in the murine urinary tract. Diabetes Obes Metab. 2020;22(9):1548–55. doi: 10.1111/dom.14064 32314507 PMC7571118

[26] Salamon K, Linn-Peirano S, Simoni A, de Dios Ruiz-Rosado J, Becknell B, John P, et al. Analysing the influence of dapagliflozin on urinary tract infection vulnerability and kidney injury in mice infected with uropathogenic *Escherichia coli*. Diabetes Obes Metab. 2025;27(1):40–53. doi: 10.1111/dom.15981 39344841 PMC11620950

[27] Deutscher J, Aké FMD, Derkaoui M, Zébré AC, Cao TN, Bouraoui H, et al. The bacterial phosphoenolpyruvate:carbohydrate phosphotransferase system: regulation by protein phosphorylation and phosphorylation-dependent protein-protein interactions. Microbiol Mol Biol Rev. 2014;78(2):231–56. doi: 10.1128/MMBR.00001-14 24847021 PMC4054256

[28] Deutscher J, Francke C, Postma PW. How phosphotransferase system-related protein phosphorylation regulates carbohydrate metabolism in bacteria. Microbiol Mol Biol Rev. 2006;70(4):939–1031. doi: 10.1128/MMBR.00024-06 17158705 PMC1698508

[29] Kok M, Bron G, Erni B, Mukhija S. Effect of enzyme I of the bacterial phosphoenolpyruvate : sugar phosphotransferase system (PTS) on virulence in a murine model. Microbiology (Reading). 2003;149(Pt 9):2645–52. doi: 10.1099/mic.0.26406-0 12949188

[30] Doucette CD, Schwab DJ, Wingreen NS, Rabinowitz JD. α-Ketoglutarate coordinates carbon and nitrogen utilization via enzyme I inhibition. Nat Chem Biol. 2011;7(12):894–901. doi: 10.1038/nchembio.685 22002719 PMC3218208

[31] Pflüger-Grau K, Görke B. Regulatory roles of the bacterial nitrogen-related phosphotransferase system. Trends Microbiol. 2010;18(5):205–14. doi: 10.1016/j.tim.2010.02.003 20202847

[32] Lux R, Jahreis K, Bettenbrock K, Parkinson JS, Lengeler JW. Coupling the phosphotransferase system and the methyl-accepting chemotaxis protein-dependent chemotaxis signaling pathways of *Escherichia coli*. Proc Natl Acad Sci U S A. 1995;92(25):11583–7. doi: 10.1073/pnas.92.25.11583 8524808 PMC40446

[33] Garrity LF, Schiel SL, Merrill R, Reizer J, Saier MH Jr, Ordal GW. Unique regulation of carbohydrate chemotaxis in *Bacillus subtilis* by the phosphoenolpyruvate-dependent phosphotransferase system and the methyl-accepting chemotaxis protein McpC. J Bacteriol. 1998;180(17):4475–80. doi: 10.1128/JB.180.17.4475-4480.1998 9721285 PMC107457

[34] Kanehisa M, Goto S. KEGG: kyoto encyclopedia of genes and genomes. Nucleic Acids Res. 2000;28(1):27–30. doi: 10.1093/nar/28.1.27 10592173 PMC102409

[35] Elbourne LDH, Tetu SG, Hassan KA, Paulsen IT. TransportDB 2.0: a database for exploring membrane transporters in sequenced genomes from all domains of life. Nucleic Acids Res. 2017;45(D1):D320–4. doi: 10.1093/nar/gkw1068 27899676 PMC5210551

[36] Altschul SF, Gish W, Miller W, Myers EW, Lipman DJ. Basic local alignment search tool. J Mol Biol. 1990;215(3):403–10. doi: 10.1016/S0022-2836(05)80360-2 2231712

[37] Wang J, Chitsaz F, Derbyshire MK, Gonzales NR, Gwadz M, Lu S, et al. The conserved domain database in 2023. Nucleic Acids Res. 2023;51(D1):D384–8. doi: 10.1093/nar/gkac1096 36477806 PMC9825596

[38] Kelley LA, Mezulis S, Yates CM, Wass MN, Sternberg MJE. The Phyre2 web portal for protein modeling, prediction and analysis. Nat Protoc. 2015;10(6):845–58. doi: 10.1038/nprot.2015.053 25950237 PMC5298202

[39] Olson RD, Assaf R, Brettin T, Conrad N, Cucinell C, Davis JJ, et al. Introducing the Bacterial and Viral Bioinformatics Resource Center (BV-BRC): a resource combining PATRIC, IRD and ViPR. Nucleic Acids Res. 2023;51(D1):D678–89. 10.1093/nar/gkac1003 PMC9825582 36350631

[40] Pearson MM, Sebaihia M, Churcher C, Quail MA, Seshasayee AS, Luscombe NM, et al. Complete genome sequence of uropathogenic Proteus mirabilis, a master of both adherence and motility. J Bacteriol. 2008;190(11):4027–37. doi: 10.1128/JB.01981-07 18375554 PMC2395036

[41] Karberg M, Guo H, Zhong J, Coon R, Perutka J, Lambowitz AM. Group II introns as controllable gene targeting vectors for genetic manipulation of bacteria. Nat Biotechnol. 2001;19(12):1162–7. doi: 10.1038/nbt1201-1162 11731786

[42] Pearson MM, Himpsl SD, Mobley HLT. Insertional Mutagenesis Protocol for Constructing Single or Sequential Mutations. Methods Mol Biol. 2019;2021:61–76. doi: 10.1007/978-1-4939-9601-8_7 31309496

[43] Erni B, Zanolari B. Glucose-permease of the bacterial phosphotransferase system. Gene cloning, overproduction, and amino acid sequence of enzyme IIGlc. J Biol Chem. 1986;261(35):16398–403. doi: 10.1016/s0021-9258(18)66579-2 3023349

[44] Saffen DW, Presper KA, Doering TL, Roseman S. Sugar transport by the bacterial phosphotransferase system. Molecular cloning and structural analysis of the *Escherichia coli ptsH*, *ptsI*, and *crr* genes. J Biol Chem. 1987;262(33):16241–53. doi: 10.1016/s0021-9258(18)47721-6 2960675

[45] Pearson MM, Shea AE, Pahil S, Smith SN, Forsyth VS, Mobley HLT. Organ agar serves as physiologically relevant alternative for *in vivo* bacterial colonization. Infect Immun. 2023;91(11):e0035523. doi: 10.1128/iai.00355-23 37850748 PMC10652904

[46] Seeger C, Poulsen C, Dandanell G. Identification and characterization of genes (*xapA*, *xapB*, and *xapR*) involved in xanthosine catabolism in *Escherichia coli*. J Bacteriol. 1995;177(19):5506–16. doi: 10.1128/jb.177.19.5506-5516.1995 7559336 PMC177358

[47] Nørholm MH, Dandanell G. Specificity and topology of the *Escherichia coli* xanthosine permease, a representative of the NHS subfamily of the major facilitator superfamily. J Bacteriol. 2001;183(16):4900–4. doi: 10.1128/JB.183.16.4900-4904.2001 11466294 PMC99545

[48] Shea AE, Marzoa J, Himpsl SD, Smith SN, Zhao L, Tran L, et al. *Escherichia coli* CFT073 Fitness Factors during Urinary Tract Infection: Identification Using an Ordered Transposon Library. Appl Environ Microbiol. 2020;86(13):e00691-20. doi: 10.1128/AEM.00691-20 32358013 PMC7301846

[49] Pearson MM, Pahil S, Forsyth VS, Shea AE, Mobley HLT. Construction of an Ordered Transposon Library for Uropathogenic *Proteus mirabilis* HI4320. Microbiol Spectr. 2022;10(6):e0314222. doi: 10.1128/spectrum.03142-22 36377916 PMC9769666

[50] Smith SN. Cochallenge Inoculation with *Proteus mirabilis* in a Murine Transurethral Urinary Tract Model of Ascending Infection. Methods Mol Biol. 2019;2021:173–86. doi: 10.1007/978-1-4939-9601-8_16 31309505

[51] Barabote RD, Saier MH Jr. Comparative genomic analyses of the bacterial phosphotransferase system. Microbiol Mol Biol Rev. 2005;69(4):608–34. doi: 10.1128/MMBR.69.4.608-634.2005 16339738 PMC1306802

[52] Makarem EH, Berk RS. Partial purification and characterization of chondroitinase from Proteus mirabilis. J Infect Dis. 1968;118(4):427–35. doi: 10.1093/infdis/118.4.427 5698696

[53] Sato N, Shimada M, Nakajima H, Oda H, Kimura S. Cloning and expression in Escherichia coli of the gene encoding the *Proteus vulgaris* chondroitin ABC lyase. Appl Microbiol Biotechnol. 1994;41(1):39–46. doi: 10.1007/BF00166079 7512814

[54] Dodgson KS, Lloyd AG. Studies on sulphatases. XVIII. Preparation of chondroitinase-free chondrosulphatase from extracts of *Proteus vulgaris*. Biochem J. 1957;66(3):532–8. 13459892 10.1042/bj0660532 PMC1200052

[55] Martinez RJ, Wolfe JB, Nakada HI. Degradation of chondroitin sulfate by *Proteus vulgaris*. J Bacteriol. 1959;78(2):217–24. doi: 10.1128/jb.78.2.217-224.1959 14421815 PMC290516

[56] Nguyen VH, Khan F, Shipman BM, Neugent ML, Hulyalkar NV, Cha NY, et al. A Semi-Quantitative Assay to Measure Glycosaminoglycan Degradation by the Urinary Microbiota. Front Cell Infect Microbiol. 2021;11:803409. doi: 10.3389/fcimb.2021.803409 35047421 PMC8762050

[57] Shipman BM, Zhou S, Hunt B, Brix V, Salaudeen I, Evers AN, et al. Strain level variation in Proteus mirabilis chondroitin sulfate degradation kinetics and regulation by urea. bioRxiv. 2026.

[58] Armbruster CE, Hodges SA, Mobley HLT. Initiation of swarming motility by *Proteus mirabilis* occurs in response to specific cues present in urine and requires excess L-glutamine. J Bacteriol. 2013;195(6):1305–19. doi: 10.1128/JB.02136-12 23316040 PMC3591990

[59] Armbruster CE, Hodges SA, Smith SN, Alteri CJ, Mobley HLT. Arginine promotes *Proteus mirabilis* motility and fitness by contributing to conservation of the proton gradient and proton motive force. Microbiologyopen. 2014;3(5):630–41. doi: 10.1002/mbo3.194 25100003 PMC4234256

[60] Schaffer JN, Pearson MM. *Proteus mirabilis* and Urinary Tract Infections. Microbiol Spectr. 2015;3(5). doi: 10.1128/microbiolspec.UTI-0017-2013 26542036 PMC4638163

[61] Gao T, Ding M, Yang C-H, Fan H, Chai Y, Li Y. The phosphotransferase system gene ptsH plays an important role in MnSOD production, biofilm formation, swarming motility, and root colonization in *Bacillus cereus* 905. Res Microbiol. 2019;170(2):86–96. doi: 10.1016/j.resmic.2018.10.002 30395927

[62] Schaffer JN, Norsworthy AN, Sun T-T, Pearson MM. *Proteus mirabilis* fimbriae- and urease-dependent clusters assemble in an extracellular niche to initiate bladder stone formation. Proc Natl Acad Sci U S A. 2016;113(16):4494–9. doi: 10.1073/pnas.1601720113 27044107 PMC4843424

[63] Yew WS, Gerlt JA. Utilization of L-ascorbate by *Escherichia coli* K-12: assignments of functions to products of the *yjf-sga* and *yia-sgb* operons. J Bacteriol. 2002;184(1):302–6. doi: 10.1128/JB.184.1.302-306.2002 11741871 PMC134747

[64] Meng W, Ellsworth BA, Nirschl AA, McCann PJ, Patel M, Girotra RN, et al. Discovery of dapagliflozin: a potent, selective renal sodium-dependent glucose cotransporter 2 (SGLT2) inhibitor for the treatment of type 2 diabetes. J Med Chem. 2008;51(5):1145–9. doi: 10.1021/jm701272q 18260618

[65] Walters MS, Lane MC, Vigil PD, Smith SN, Walk ST, Mobley HLT. Kinetics of uropathogenic *Escherichia coli* metapopulation movement during urinary tract infection. mBio. 2012;3(1):e00303-11. doi: 10.1128/mBio.00303-11 22318320 PMC3273315

[66] Hannan TJ, Totsika M, Mansfield KJ, Moore KH, Schembri MA, Hultgren SJ. Host-pathogen checkpoints and population bottlenecks in persistent and intracellular uropathogenic *Escherichia coli* bladder infection. FEMS Microbiol Rev. 2012;36(3):616–48. doi: 10.1111/j.1574-6976.2012.00339.x 22404313 PMC3675774

[67] Mueckler M, Thorens B. The SLC2 (GLUT) family of membrane transporters. Mol Aspects Med. 2013;34(2–3):121–38. doi: 10.1016/j.mam.2012.07.001 23506862 PMC4104978

[68] Rosen DA, Hung C-S, Kline KA, Hultgren SJ. Streptozocin-induced diabetic mouse model of urinary tract infection. Infect Immun. 2008;76(9):4290–8. doi: 10.1128/IAI.00255-08 18644886 PMC2519435

[69] Keyhani NO, Roseman S. Wild-type *Escherichia coli* grows on the chitin disaccharide, N,N’-diacetylchitobiose, by expressing the cel operon. Proc Natl Acad Sci U S A. 1997;94(26):14367–71. 9405618 10.1073/pnas.94.26.14367 PMC24980

[70] Yeh H-Y, Line JE, Hinton A Jr. Molecular Analysis, Biochemical Characterization, Antimicrobial Activity, and Immunological Analysis of *Proteus mirabilis* Isolated from Broilers. J Food Sci. 2018;83(3):770–9. doi: 10.1111/1750-3841.14056 29437227

[71] Lee C-R, Park Y-H, Kim M, Kim Y-R, Park S, Peterkofsky A, et al. Reciprocal regulation of the autophosphorylation of enzyme INtr by glutamine and α-ketoglutarate in *Escherichia coli*. Mol Microbiol. 2013;88(3):473–85. doi: 10.1111/mmi.12196 23517463 PMC3633653

[72] Ozer A, Altuntas CZ, Bicer F, Izgi K, Hultgren SJ, Liu G, et al. Impaired cytokine expression, neutrophil infiltration and bacterial clearance in response to urinary tract infection in diabetic mice. Pathog Dis. 2015;73(3):ftv002. doi: 10.1093/femspd/ftv002 25663347 PMC4443837

[73] Yang T, Zhou Y, Cui Y. Urinary tract infections and genital mycotic infections associated with SGLT‑2 inhibitors: an analysis of the FDA Adverse Event Reporting System. Expert Opin Drug Saf. 2024;23(8):1035–40. doi: 10.1080/14740338.2023.2288897 38009230

[74] Puckrin R, Saltiel M-P, Reynier P, Azoulay L, Yu OHY, Filion KB. SGLT-2 inhibitors and the risk of infections: a systematic review and meta-analysis of randomized controlled trials. Acta Diabetol. 2018;55(5):503–14. doi: 10.1007/s00592-018-1116-0 29484489

[75] Suzuki M, Hiramatsu M, Fukazawa M, Matsumoto M, Honda K, Suzuki Y, et al. Effect of SGLT2 inhibitors in a murine model of urinary tract infection with *Candida albicans*. Diabetes Obes Metab. 2014;16(7):622–7. doi: 10.1111/dom.12259 24400675

[76] Schwartz L, Simoni A, Yan P, Salamon K, Turkoglu A, Vasquez Martinez G, et al. Insulin receptor orchestrates kidney antibacterial defenses. Proc Natl Acad Sci U S A. 2024;121(29):e2400666121. doi: 10.1073/pnas.2400666121 38976738 PMC11260129

[77] Murtha MJ, Eichler T, Bender K, Metheny J, Li B, Schwaderer AL, et al. Insulin receptor signaling regulates renal collecting duct and intercalated cell antibacterial defenses. J Clin Invest. 2018;128(12):5634–46. doi: 10.1172/JCI98595 30418175 PMC6264632

[78] Tsai Y-L, Chien H-F, Huang K-T, Lin W-Y, Liaw S-J. cAMP receptor protein regulates mouse colonization, motility, fimbria-mediated adhesion, and stress tolerance in uropathogenic *Proteus mirabilis*. Sci Rep. 2017;7(1):7282. doi: 10.1038/s41598-017-07304-7 28779108 PMC5544767

[79] Mobley HL, Chippendale GR. Hemagglutinin, urease, and hemolysin production by Proteus mirabilis from clinical sources. J Infect Dis. 1990;161(3):525–30. doi: 10.1093/infdis/161.3.525 2179424

[80] Belas R, Erskine D, Flaherty D. Transposon mutagenesis in *Proteus mirabilis*. J Bacteriol. 1991;173(19):6289–93. doi: 10.1128/jb.173.19.6289-6293.1991 1655704 PMC208382

[81] Pearson MM, Mobley HLT. The type III secretion system of Proteus mirabilis HI4320 does not contribute to virulence in the mouse model of ascending urinary tract infection. J Med Microbiol. 2007;56(Pt 10):1277–83. doi: 10.1099/jmm.0.47314-0 17893161

[82] Heap JT, Kuehne SA, Ehsaan M, Cartman ST, Cooksley CM, Scott JC, et al. The ClosTron: Mutagenesis in *Clostridium* refined and streamlined. J Microbiol Methods. 2010;80(1):49–55. doi: 10.1016/j.mimet.2009.10.018 19891996

[83] Davanloo P, Rosenberg AH, Dunn JJ, Studier FW. Cloning and expression of the gene for bacteriophage T7 RNA polymerase. Proc Natl Acad Sci U S A. 1984;81(7):2035–9. doi: 10.1073/pnas.81.7.2035 6371808 PMC345431

[84] Pearson MM, Rasko DA, Smith SN, Mobley HLT. Transcriptome of swarming *Proteus mirabilis*. Infect Immun. 2010;78(6):2834–45. doi: 10.1128/IAI.01222-09 20368347 PMC2876570

[85] Liu Q, Li MZ, Leibham D, Cortez D, Elledge SJ. The univector plasmid-fusion system, a method for rapid construction of recombinant DNA without restriction enzymes. Curr Biol. 1998;8(24):1300–9. doi: 10.1016/s0960-9822(07)00560-x 9843682

[86] Hagberg L, Engberg I, Freter R, Lam J, Olling S, Svanborg Edén C. Ascending, unobstructed urinary tract infection in mice caused by pyelonephritogenic *Escherichia coli* of human origin. Infect Immun. 1983;40(1):273–83. doi: 10.1128/iai.40.1.273-283.1983 6339403 PMC264845

[87] Johnson DE, Lockatell CV, Hall-Craigs M, Mobley HL, Warren JW. Uropathogenicity in rats and mice of *Providencia stuartii* from long-term catheterized patients. J Urol. 1987;138(3):632–5. doi: 10.1016/s0022-5347(17)43287-3 3625871

[88] Garcia EC, Brumbaugh AR, Mobley HLT. Redundancy and specificity of Escherichia coli iron acquisition systems during urinary tract infection. Infect Immun. 2011;79(3):1225–35. doi: 10.1128/IAI.01222-10 21220482 PMC3067483

[89] Forsyth VS, Mobley HLT, Armbruster CE. Transposon Insertion Site Sequencing in a Urinary Tract Model. Methods Mol Biol. 2019;2021:297–337. doi: 10.1007/978-1-4939-9601-8_25 31309514

[90] English MA, Alcantar MA, Collins JJ. A self-propagating, barcoded transposon system for the dynamic rewiring of genomic networks. Mol Syst Biol. 2023;19(6):e11398. doi: 10.15252/msb.202211398 36970845 PMC10258560

[91] Goodman AL, Wu M, Gordon JI. Identifying microbial fitness determinants by insertion sequencing using genome-wide transposon mutant libraries. Nat Protoc. 2011;6(12):1969–80. doi: 10.1038/nprot.2011.417 22094732 PMC3310428

[92] Robinson MD, McCarthy DJ, Smyth GK. edgeR: a Bioconductor package for differential expression analysis of digital gene expression data. Bioinformatics. 2010;26(1):139–40. doi: 10.1093/bioinformatics/btp616 19910308 PMC2796818

[93] Lane MC, Alteri CJ, Smith SN, Mobley HLT. Expression of flagella is coincident with uropathogenic *Escherichia coli* ascension to the upper urinary tract. Proc Natl Acad Sci U S A. 2007;104(42):16669–74. doi: 10.1073/pnas.0607898104 17925449 PMC2034267

[94] Solovyev V, Salamov A. Automatic annotation of microbial genomes and metagenomic sequences. In: Li RW, editor. Metagenomics and its Applications in Agriculture, Biomedicine and Environmental Studies. Hauppauge (NY): Nova Science Publishers; 2011. p. 61–78.

[95] Nishitani S, Fukuhara A, Shin J, Okuno Y, Otsuki M, Shimomura I. Metabolomic and microarray analyses of adipose tissue of dapagliflozin-treated mice, and effects of 3-hydroxybutyrate on induction of adiponectin in adipocytes. Sci Rep. 2018;8(1):8805. doi: 10.1038/s41598-018-27181-y 29891844 PMC5995811

[96] Midani FS, Collins J, Britton RA. AMiGA: Software for Automated Analysis of Microbial Growth Assays. mSystems. 2021;6(4):e0050821. doi: 10.1128/mSystems.00508-21 34254821 PMC8409736

[97] Pearson MM. Methods for Studying Swarming and Swimming Motility. Methods Mol Biol. 2019;2021:15–25. doi: 10.1007/978-1-4939-9601-8_3 31309492

[98] Pearson MM. Phase Variation of the *mrp* Fimbrial Promoter. Methods Mol Biol. 2019;2021:121–7. doi: 10.1007/978-1-4939-9601-8_12 31309501

[99] Christensen WB. Urea Decomposition as a Means of Differentiating Proteus and Paracolon Cultures from Each Other and from *Salmonella* and *Shigella* Types. J Bacteriol. 1946;52(4):461–6. doi: 10.1128/jb.52.4.461-466.1946 16561200 PMC518212

[100] Robinson MD, Smyth GK. Small-sample estimation of negative binomial dispersion, with applications to SAGE data. Biostatistics. 2008;9(2):321–32. doi: 10.1093/biostatistics/kxm030 17728317

