# Supplementary figures and images for: Selective sugar transport supports Proteus mirabilis fitness in the urinary tract
Source: PLoS Pathog. 2026 Jun 23;22(6):e1014324. doi: 10.1371/journal.ppat.1014324 (PMC13289894; doi:10.1371/journal.ppat.1014324)

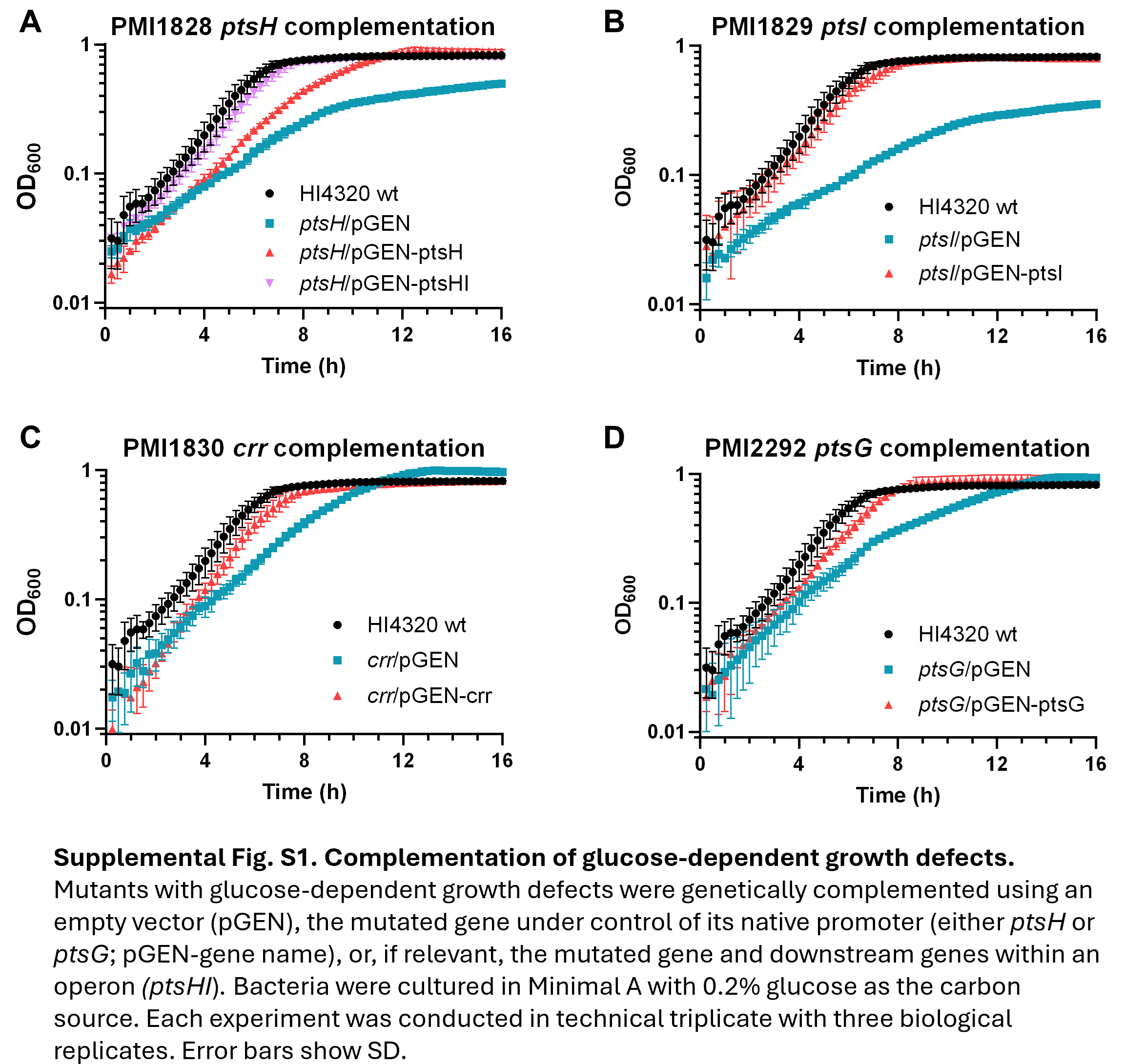

Supplement: S1 Fig — Mutants with glucose-dependent growth defects were genetically complemented using an empty vector (pGEN), the mutated gene under control of its native promoter (either ptsH or ptsG promoters; pGEN-gene name), or, if relevant, the mutated gene and downstream genes within an operon (ptsHI). Bacteria were cultured in Minimal A with 0.2% glucose as the carbon source. Each experiment was conducted in technical triplicate with three biological replicates. Error bars show SD. (TIF) [file ppat.1014324.s007.tif]

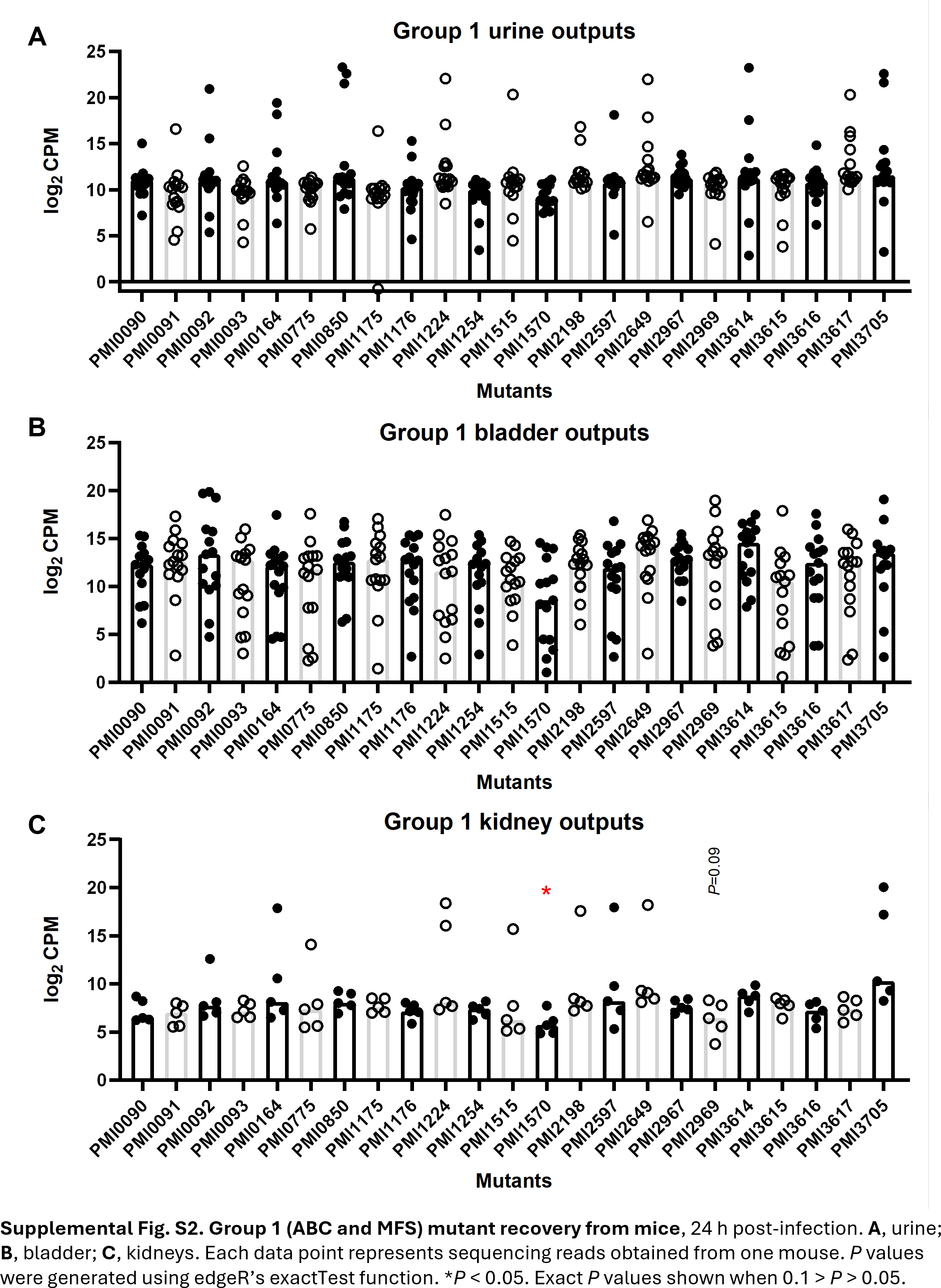

Supplement: S2 Fig — (A) urine; (B) bladder; (C) kidneys. Each data point represents sequencing reads obtained from one mouse. P values were generated using edgeR’s exactTest function. *P < 0.05. Exact P values shown when 0.1 > P > 0.05. (TIF) [file ppat.1014324.s008.tif]

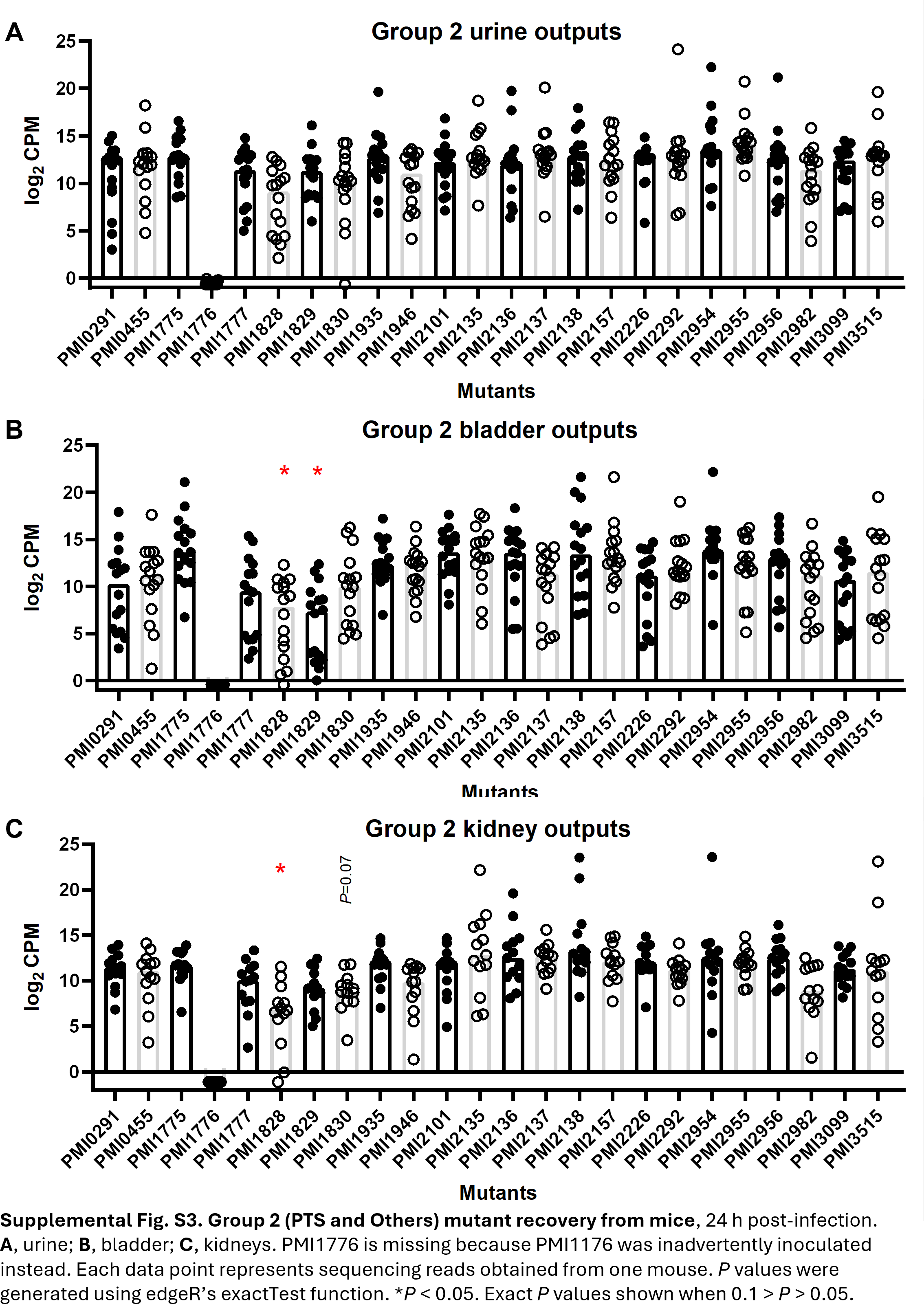

Supplement: S3 Fig — (A) urine; (B) bladder; (C) kidneys. PMI1776 is missing because PMI1176 was inadvertently inoculated instead. Each data point represents sequencing reads obtained from one mouse. P values were generated using edgeR’s exactTest function. *P < 0.05. Exact P values shown when 0.1 > P > 0.05. (TIF) [file ppat.1014324.s009.tif]

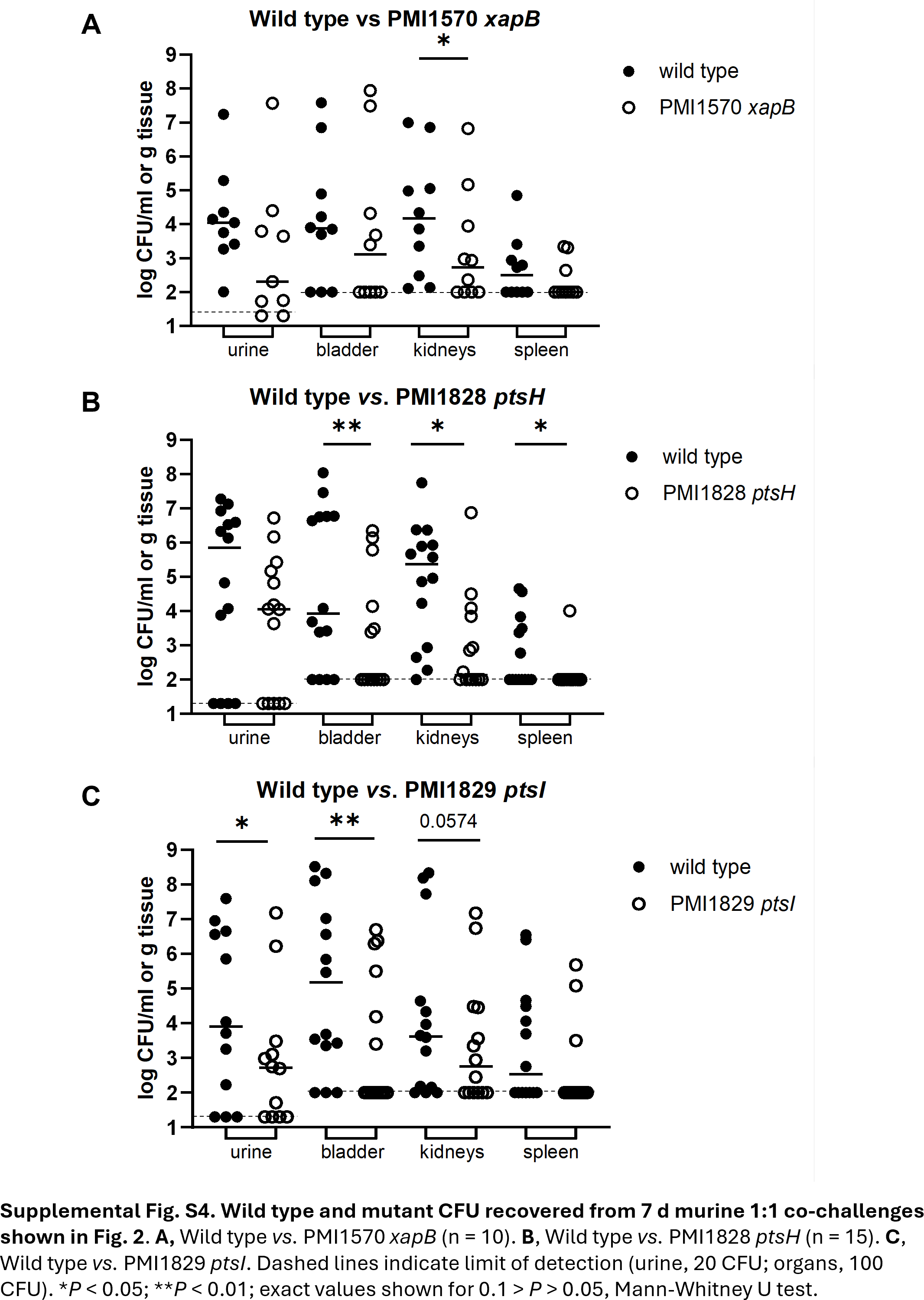

Supplement: S4 Fig — (A) Wild type vs. PMI1570 xapB (n = 10). (B) Wild type vs. PMI1828 ptsH (n = 15). C, Wild type vs. PMI1829 ptsI. Dashed lines indicate limit of detection (urine, 20 CFU; organs, 100 CFU). *P < 0.05; **P < 0.01; exact values shown for 0.1 > P > 0.05, Mann-Whitney U test. Horizontal lines denote medians. (TIF) [file ppat.1014324.s010.tif]

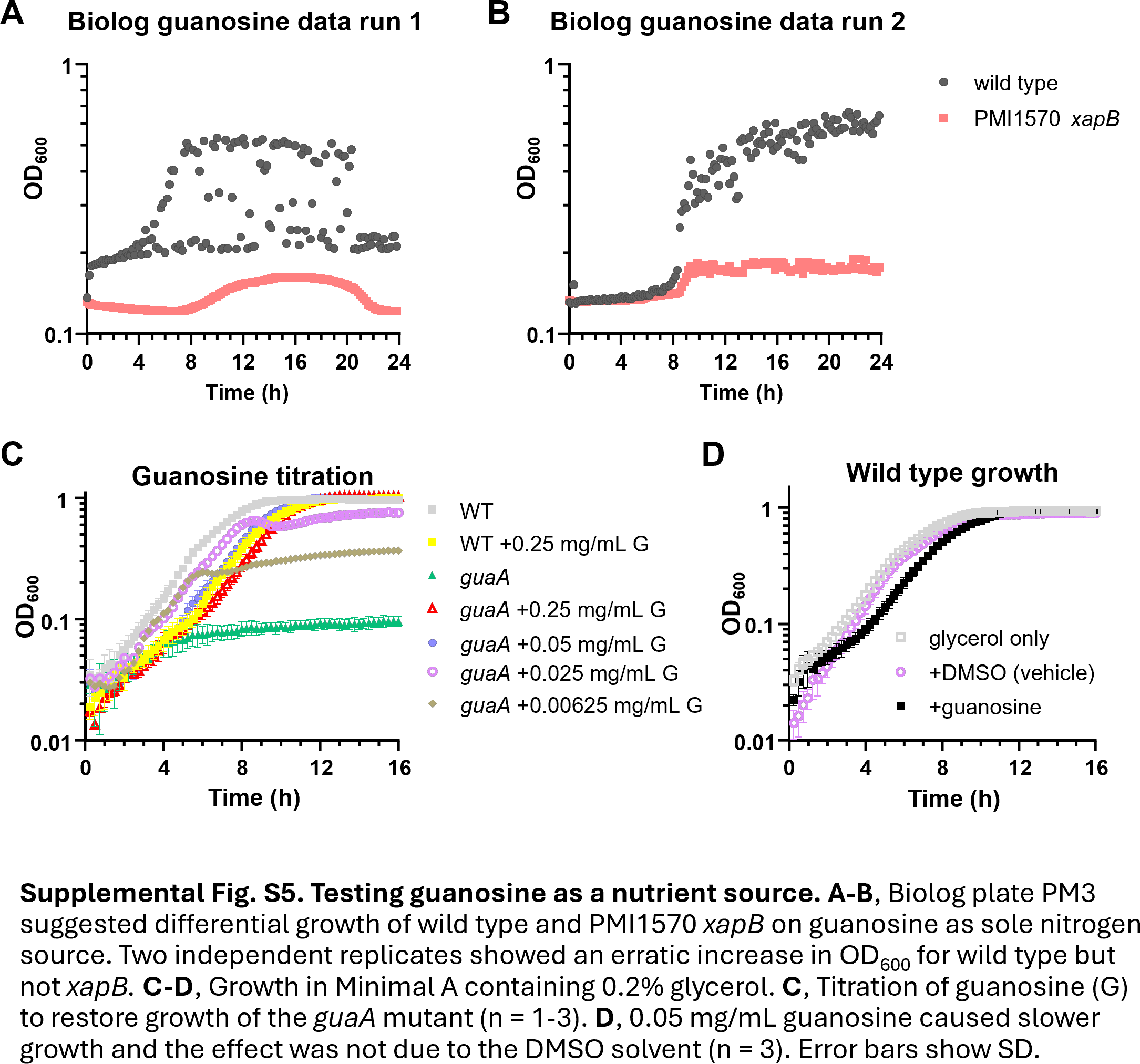

Supplement: S5 Fig — A-B, Biolog plate PM3 suggested differential growth of wild type and PMI1570 xapB on guanosine as sole nitrogen source. Two independent replicates are separately shown to highlight the extreme variability of erratic increase in OD600 for wild type but not xapB. C-D, Growth in Minimal A containing 0.2% glycerol. (C) Titration of guanosine (G) to restore growth of the guaA mutant (n = 1–3). (D) 0.05 mg/mL guanosine caused slower growth and the effect was not due to the DMSO solvent (n = 3). Error bars show SD. (TIF) [file ppat.1014324.s011.tif]

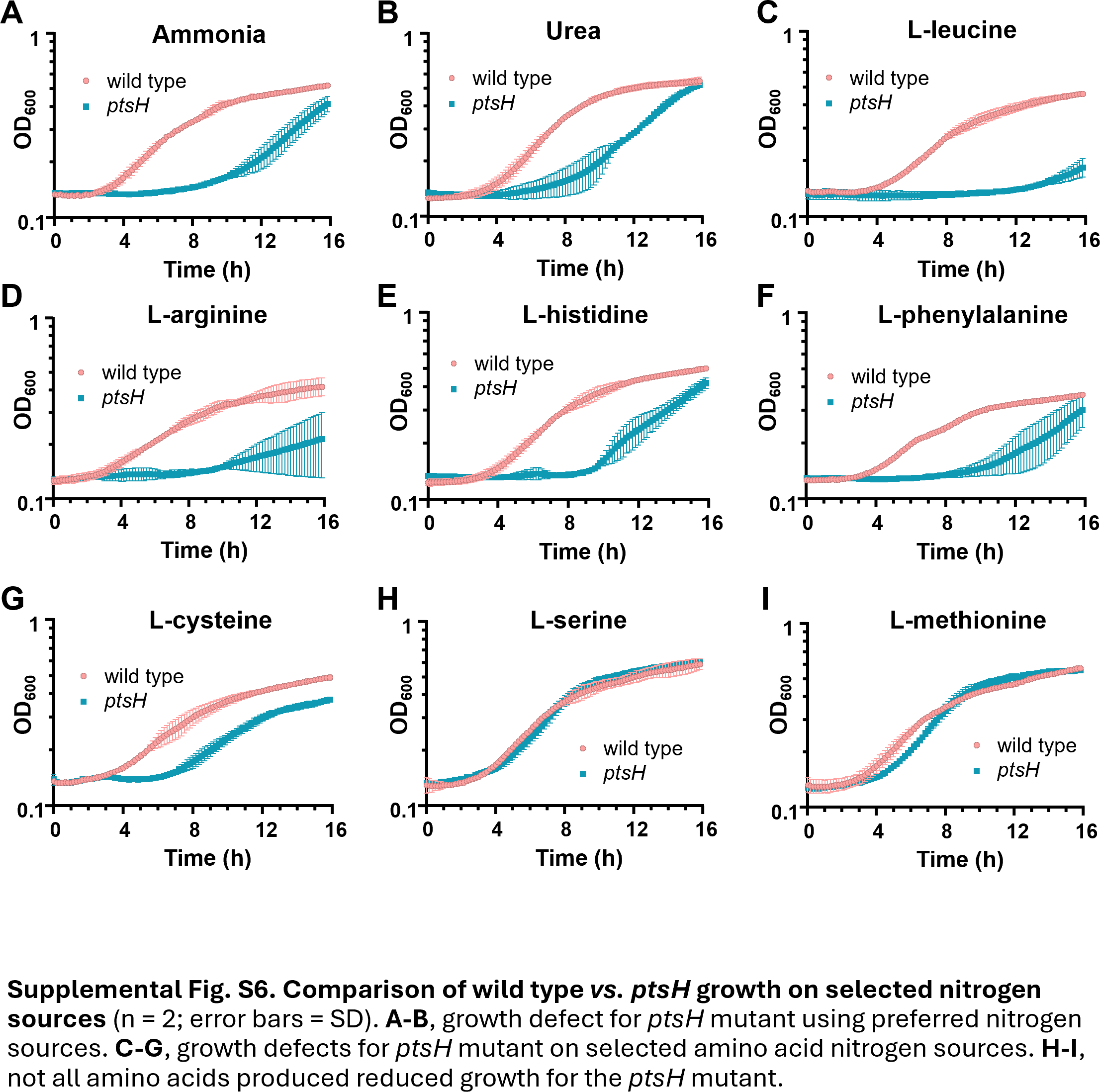

Supplement: S6 Fig — A-B, growth defect for ptsH mutant using preferred nitrogen sources. C-G, growth defects for ptsH mutant on selected amino acid nitrogen sources. H-I, not all amino acids produced reduced growth for the ptsH mutant. (TIF) [file ppat.1014324.s012.tif]

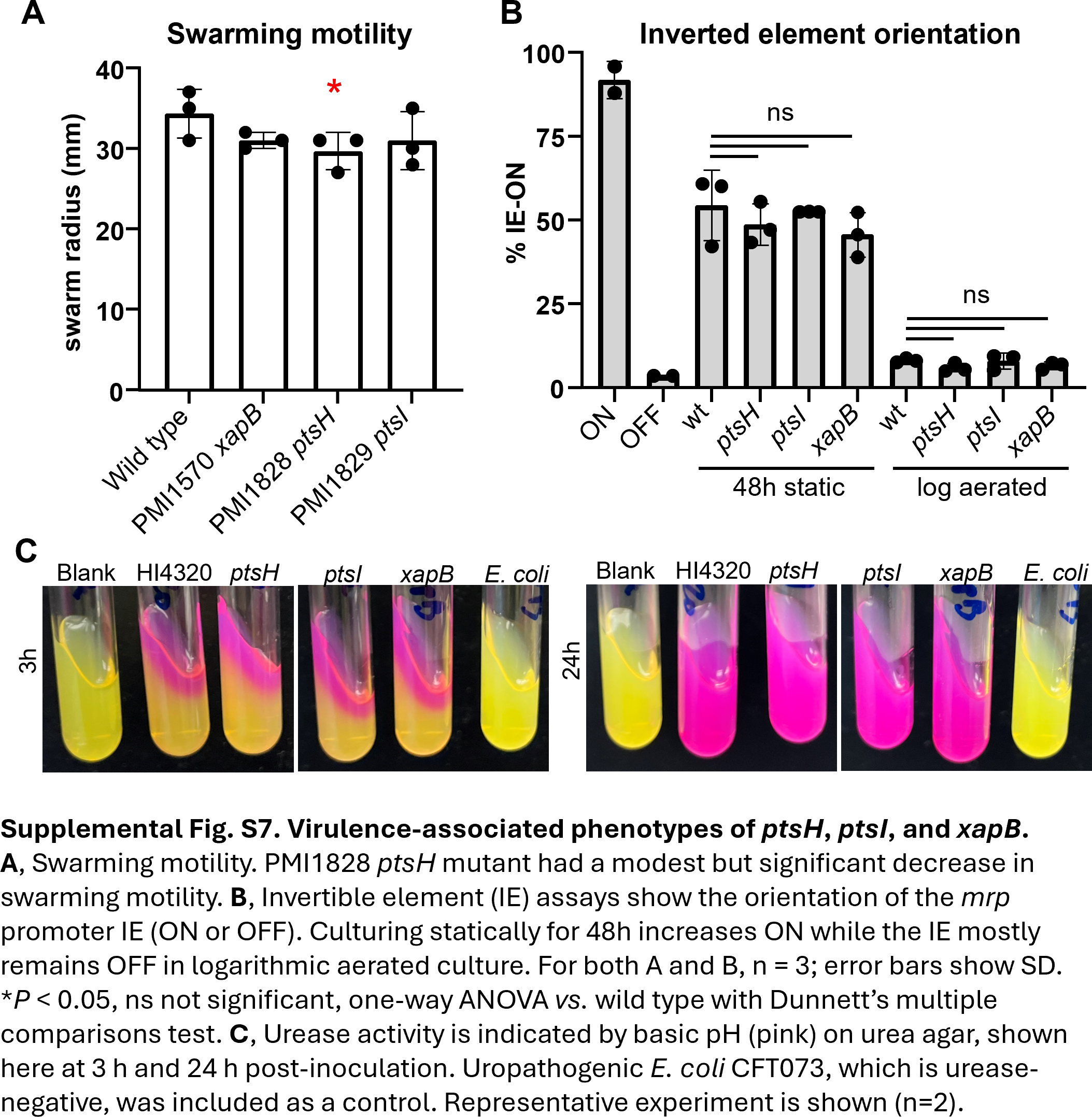

Supplement: S7 Fig — (A) Swarming motility. PMI1828 ptsH mutant had a modest but significant decrease in swarming motility. (B) Invertible element (IE) assays show the orientation of the mrp promoter IE (ON or OFF). Culturing statically for 48h increases ON while the IE mostly remains OFF in logarithmic aerated culture. For both A and B, n = 3; error bars show SD. *P < 0.05, ns not significant, one-way ANOVA vs. wild type with Dunnett’s multiple comparisons test. (C) Urease activity is indicated by basic pH (pink) on urea agar, shown here at 3 h and 24 h post-inoculation. Uropathogenic E. coli CFT073, which is urease-negative, was included as a control. Representative experiment is shown (n = 2). (TIF) [file ppat.1014324.s013.tif]

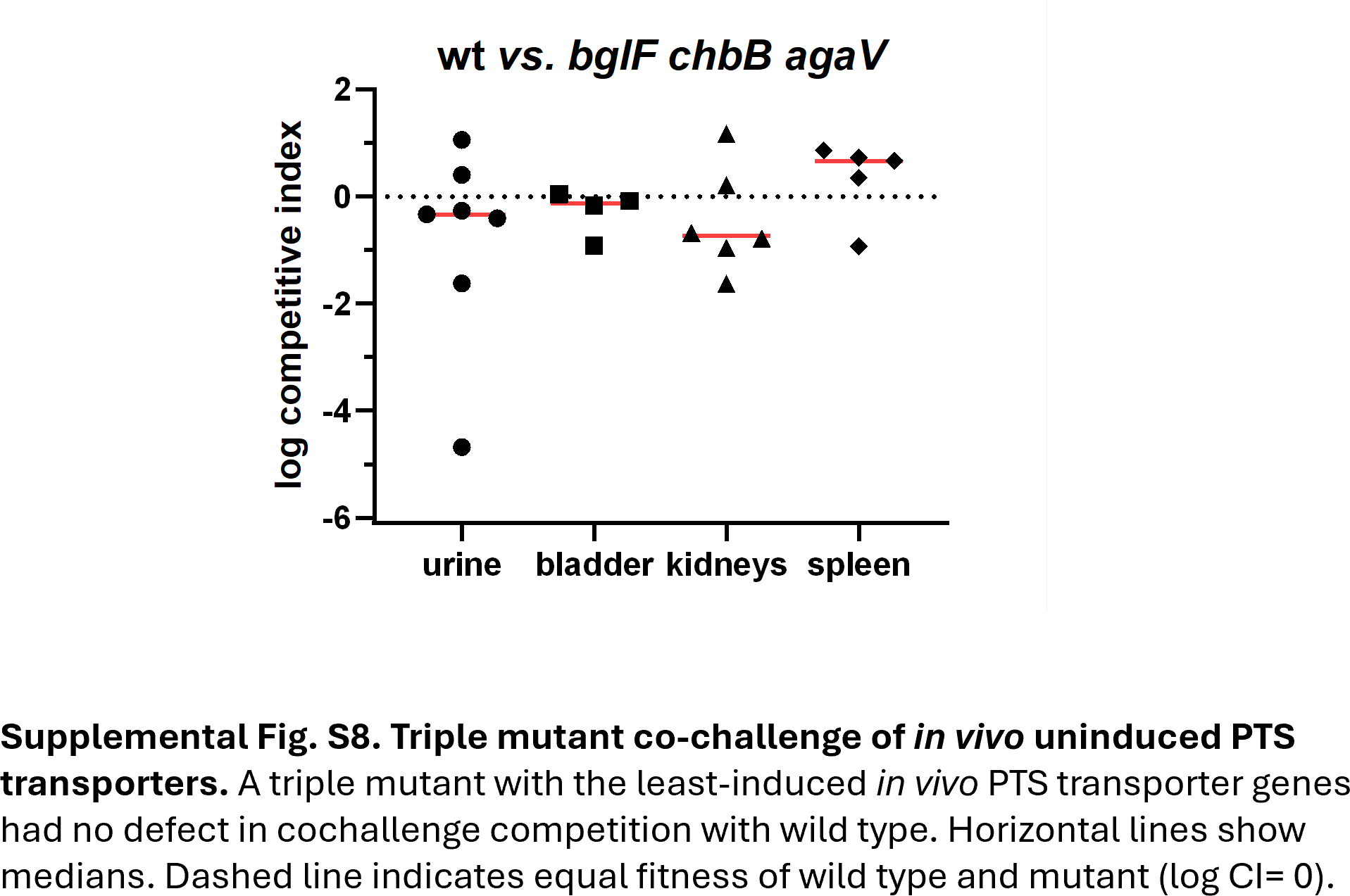

Supplement: S8 Fig — A triple mutant with the least-induced in vivo PTS transporter genes had no defect in co-challenge competition with wild type. Horizontal lines show medians. Dashed line indicates equal fitness of wild type and mutant (log CI = 0). (TIF) [file ppat.1014324.s014.tif]

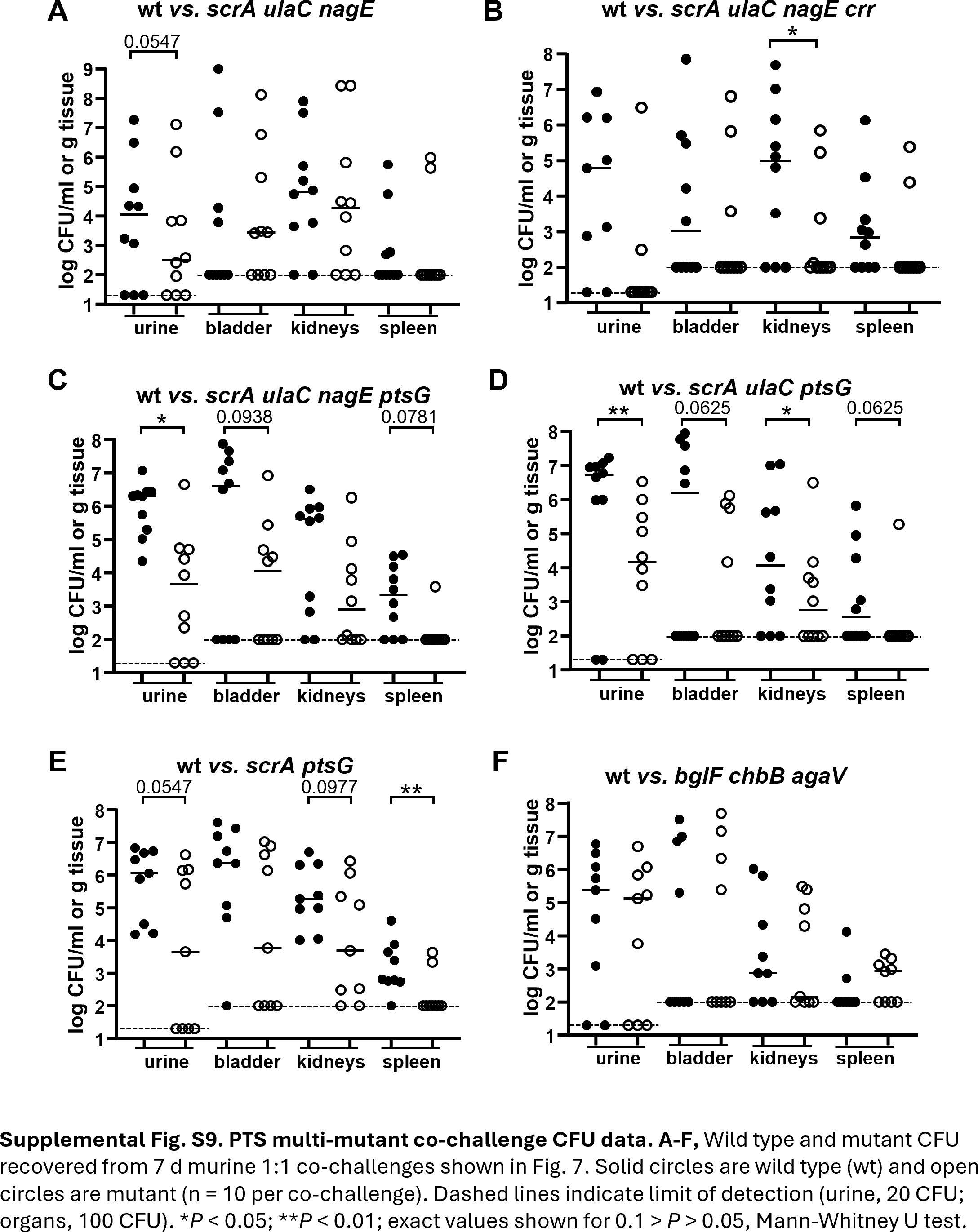

Supplement: S9 Fig — A-F, Wild type and mutant CFU recovered from 7 d murine 1:1 co-challenges shown in Fig. 7. Solid circles are wild type (wt) and open circles are mutant (n = 10 per co-challenge). Dashed lines indicate limit of detection (urine, 20 CFU; organs, 100 CFU). *P < 0.05; **P < 0.01; exact values shown for 0.1 > P > 0.05, Mann-Whitney U test. Horizontal lines denote medians. (TIF) [file ppat.1014324.s015.tif]

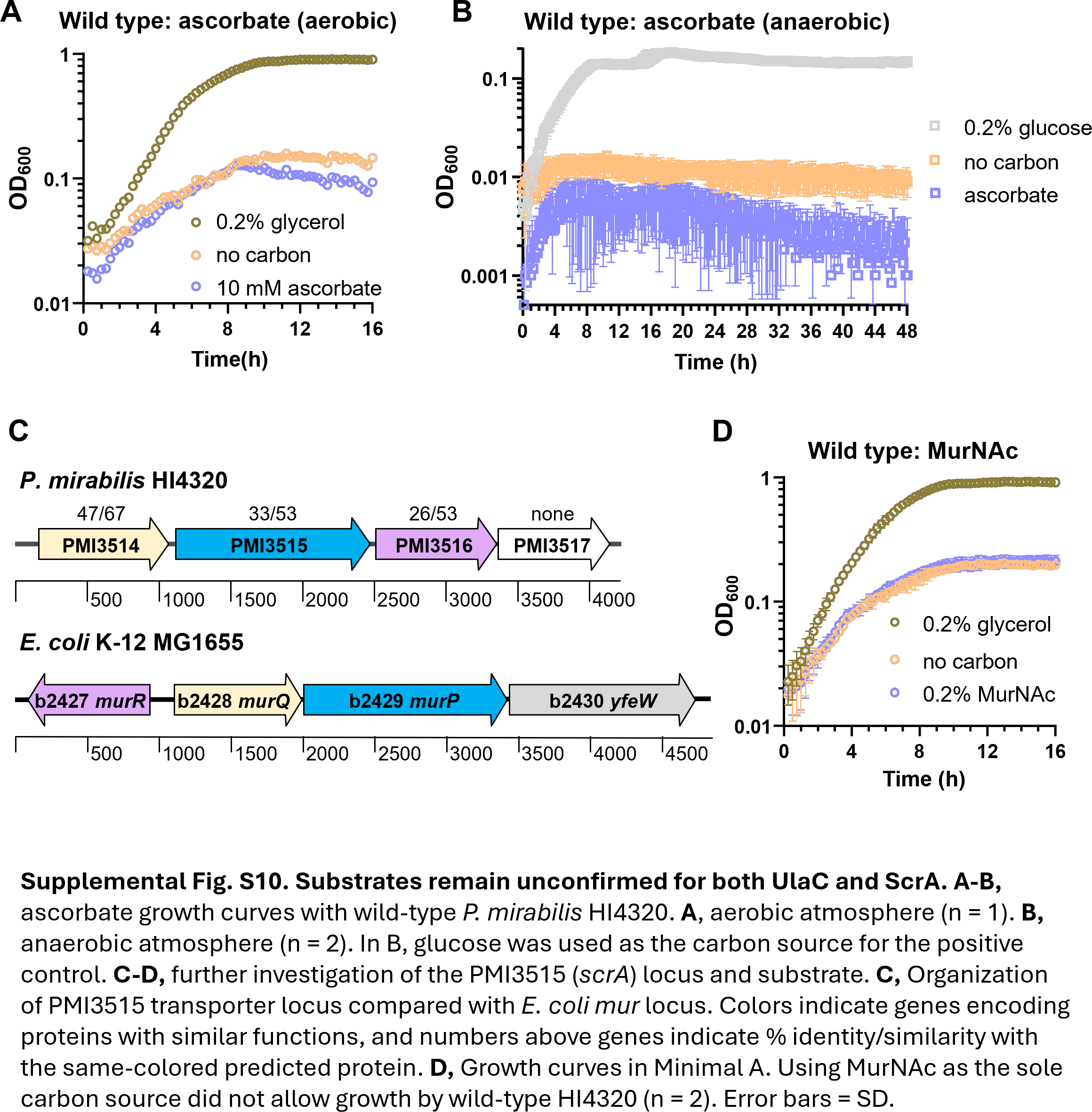

Supplement: S10 Fig — A-B, ascorbate growth curves with wild-type P. mirabilis HI4320. (A) aerobic atmosphere (n = 1). (B) anaerobic atmosphere (n = 2). In B, glucose was used as the carbon source for the positive control. C-D, further investigation of the PMI3515 (scrA) locus and substrate. (C) Organization of PMI3515 transporter locus compared with E. coli mur locus. Colors indicate genes encoding proteins with similar functions, and numbers above genes indicate % identity/similarity with the same-colored predicted protein. (D) Growth curves in Minimal A. Using MurNAc as the sole carbon source did not allow growth by wild-type HI4320 (n = 2). Error bars = SD. (TIF) [file ppat.1014324.s016.tif]

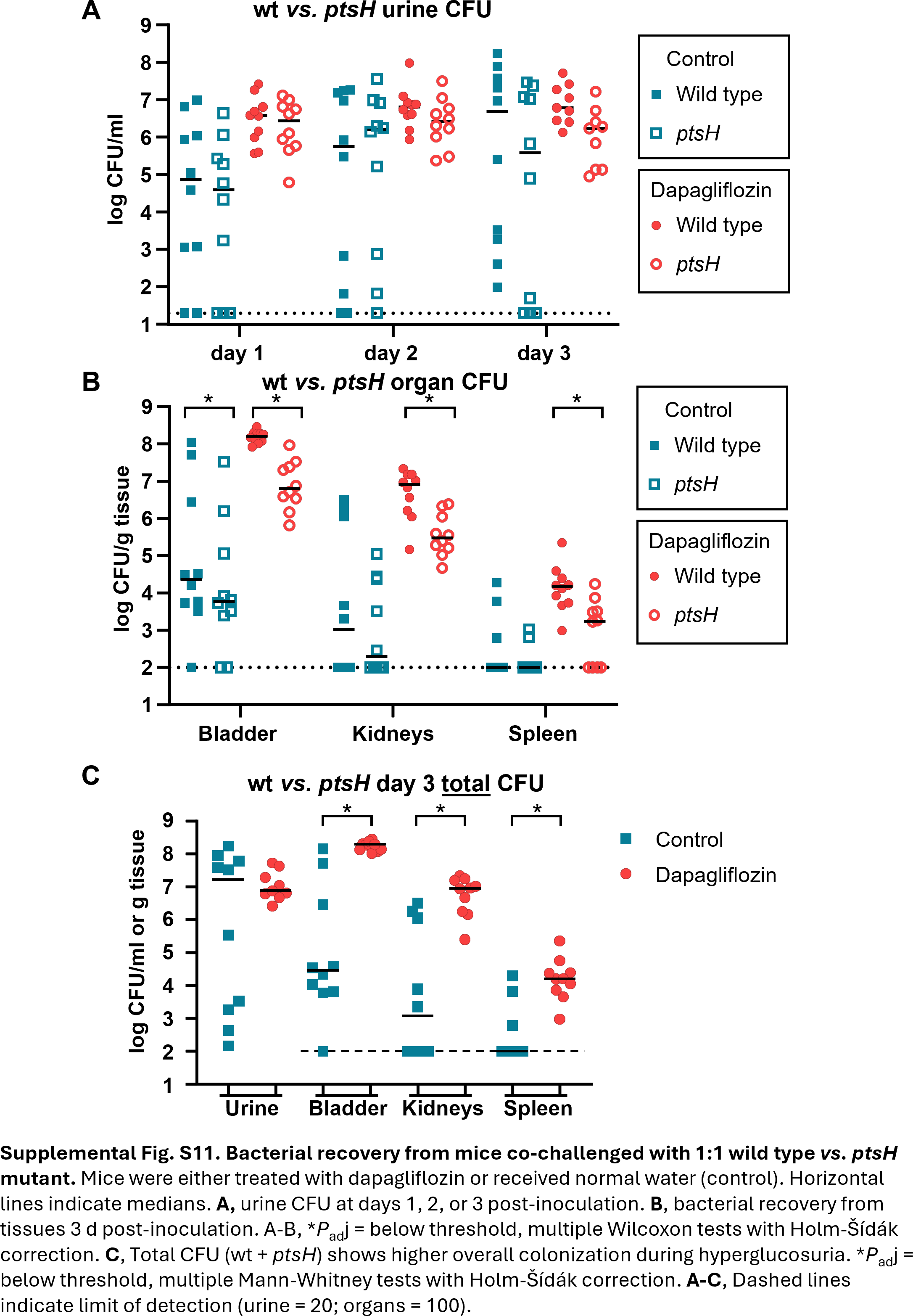

Supplement: S11 Fig — Mice were either treated with dapagliflozin or received normal water (control). Horizontal lines indicate medians. (A) urine CFU at days 1, 2, or 3 post-inoculation. (B) bacterial recovery from tissues 3 d post-inoculation. A-B, *Padj = below threshold, multiple Wilcoxon tests with Holm-Šídák correction. (C) Total CFU (wt + ptsH) shows higher overall colonization during hyperglucosuria. *Padj = below threshold, multiple Mann-Whitney tests with Holm-Šídák correction. A-C, Dashed lines indicate limit of detection (urine = 20; organs = 100). (TIF) [file ppat.1014324.s017.tif]
