# Supplementary material for: Selective sugar transport supports Proteus mirabilis fitness in the urinary tract
Source: PLoS Pathog. 2026 Jun 23;22(6):e1014324. doi: 10.1371/journal.ppat.1014324 (PMC13289894; doi:10.1371/journal.ppat.1014324)
Supplement: Attachment — Submitted filename: response to reviews.docx [file ppat.1014324.s022.docx]

We thank the editor and reviewers for their careful evaluation of our manuscript and for their constructive feedback. The reviewers recognized the rigor of the experimental design, the robustness of the mutant screen, and the significance of identifying sugar transport systems that contribute to *P. mirabilis* fitness during UTI. They also agreed with our emphasis on the need for experimental validation and less reliance on pre-existing gene annotations. The novelty of demonstrating that induced glucosuria exacerbates *P. mirabilis* colonization was also noted. The reviewers recognized that this work will be of interest to researchers in the fields of host-pathogen interaction and diabetic complications.
 In response to the comments, we have revised the manuscript to strengthen data presentation and interpretation, improve clarity, and address key concerns. These revisions include the addition of *in vitro* complementation data for central PTS mutants and assessment of canonical virulence traits such as urease activity and MR/P fimbrial promoter orientation. Most revisions were editorial in nature. These included expanding supporting datasets, adjusting citations, and providing additional detail in figure legends. We have also refined our conclusions and incorporated additional discussion of limitations and alternative interpretations where appropriate. Collectively, these changes strengthen the manuscript and more clearly support our conclusions regarding the role of sugar transport systems in *P. mirabilis* pathogenesis. Our point-by-point responses follow:

**Editor’s note: Reviewers 1 and 3, in particular, raise a number of points that should be addressed, including the genetic complementation of key mutants in at least some assays.**

>We have added genetic complementation for the observed *in vitro* growth phenotypes, now shown in new Fig. S1. All other points are addressed below, including addition of new Dataset S3 providing complete raw data for all Biolog Phenotype MicroArray growth curves.

**Reviewer #1: Have the Authors investigated the growth of sugar transport mutants in human urine, and urine supplemented with glucose to resemble diabetic urine? This seems like a straightforward experiment that could yield critical insights to strengthen this manuscript.**

| ***P. mirabilis* urine dunk** | | |
| --- | --- | --- |
| **Condition** | ***rpoA* C_t_** | **pH** |
| LB 30 min | 11.94 | 6.8 |
| urine 30 min | 15.9 | 8.5 |
| urine 1 hr | 18.43 | 9 |
| urine 2 hr | 26.15 | >9 |

>While growth in human urine is a common and straightforward experiment used for uropathogenic *Escherichia coli*, these experiments are difficult to conduct and interpret for *P. mirabilis* due to its robust urease activity. In our experience, culturing urease-positive *P. mirabilis* in urine leads to rapid alkalinization, with pH >9 within 3 hours, and allowing for less than 1 log of growth before stationary phase. This level of alkalinity causes mineral precipitation that interferes with optical density and growth measurements and induces rapid transcriptional shutdown (<30 minutes). We are including here unpublished data from an old “urine dunk” experiment that demonstrates this shutdown via expression of housekeeping gene *rpoA* (RNA polymerase A). In this experiment, an LB log-phase culture was pelleted and resuspended in either fresh LB or pooled human urine. The shutdown of *rpoA* is apparent. This is in contrast to the urinary tract, which is continuously replenished with fresh urine and supplemented by host tissues. For these reasons, we focused on *in vivo* models and controlled *in vitro* media conditions.

**Do the mutants with severe in vivo fitness defect exhibit changes in known virulence factors such as urease? Adding this information is needed to clarify decreased fitness due to poor in vivo growth as opposed to decreased pathogenicity.**

>We agree that distinguishing generalized growth defects from alterations in pathogenicity is an important consideration; however, we anticipate that disruption of sugar transporters primarily impacts metabolic fitness rather than canonical virulence traits. Nonetheless, because of prior reports of a PTS-swarming connection in *Bacillus*, we did include swarming data in our original manuscript submission. Figure S6 demonstrated that only *ptsH* had a slight swarming defect, despite a strong defect *in vivo*. We have now added additional data showing no apparent difference in *mrp* fimbrial promoter invertible element orientation or urease activity for any of the mutants with *in vivo* fitness defects (new Fig. S7B-C and lines 362-5).

**There seems to be bacterial growth in Fig. 3A without a C source? This should be explained.**

>The limited growth observed in Figure 3A reflects residual nutrients carried over from the LB-cultured inoculum. Using direct transfer from LB into Minimal A without washing allows for consistent and reproducible data. We found that washing the bacteria or direct inoculation into minimal medium results in variable or no growth, even for wild type HI4320. Despite this small amount of growth from medium carryover (which appears as a nearly flat line when displayed at linear scale instead of log), we observe clear differences in growth phenotypes. We have now clarified this approach in the methods section (lines 794-6).

**Fig 8 A&B: Baseline glucose (day -1) is almost double in panel A, compared to B, and also post-treatment differences between these panels. Potential reasons for this change should be included.**

>We thank the reviewer for noticing these differences. The baseline (day -1) in Fig. 8A & B were obtained from two independent mouse cohorts and experiments, representing true biological replicates. Only a few studies have measured baseline glucose in the CBA/J mouse strain, so the biological variation is not yet well established. In PMID 32314507, CBA/J mice treated with an SGLT2i via oral gavage displayed baseline urinary glucose levels ~50 mg/dL in Fig. 1A. In contrast, Fig. 1D reports ~5 mg/dL representing approximately a 90% reduction. These were performed using the same age, sex and strain of mice as in our study. We similarly observed a ~67% decrease from our Fig. 8A & B. These differences likely reflect biological and experimental variability, including factors such as hydration status, feeding, and timing of sample collection.
 Importantly, induction of hyperglucosuria resulted in a clear and reproducible increase in urinary glucose relative to baseline in both experiments, independent of inherent biological variation. The differences observed post-treatment reflect the presence of infection with HI4320 in Fig. 8B compared to the uninfected condition in Fig. 8A. We hypothesize that bacterial consumption contributes to the reduction of urinary glucose post-infection (line 469). Consistent with this interpretation, hyperglucosuria coincides with increased *P. mirabilis* burden, which has been observed for different uropathogens by other groups (PMID: 32314507, 18644886, 39344841).

**Fig. 7A: Error bars should be included to inform the readers of the range of data points**

>We thank the reviewer for this comment and apologize for the lack of clarity in the original figure. We have revised Fig. 7A to display all individual points (n=9) mined from the original published microarray study and noted which genes were found to be significantly differentially regulated during experimental UTI in that study (PMID: 21505083). We feel this is more appropriate than error bars, given that statistical analysis was applied to the entire published microarray dataset. In addition to the reference in the main text, the figure legend now explicitly states that these data were not generated *de novo* as part of this study, but depict a subset of a previously published dataset (lines 376-9). This revision improves transparency and more clearly represents the data distribution. Thanks to this helpful comment, we were prompted to revisit the original data which led to the strengthening of rationale for downstream mutant selection (lines 369-70).

**Fig. 1A: Please add gene names at least for the ones that have growth defects.**

>Gene names for the four significant growth defect strains have been added to Fig. 1A. These genes are also listed alongside the gene locus numbers in Fig. 1B-C, and the full list of predictions and annotations are in Table 1. We added a new reference to Table 1 in the Fig. 1 legend (line 175).

**Reviewer #2: Overall, the data presented are sound, the manuscript is very well written, and the work will be of broad interest to researchers studying urinary tract infections, bacterial metabolism, and host-pathogen interactions.

I have no substantive concerns regarding the data or major conclusions. I cannot fault the work and congratulate the authors on an excellent study.**

>We thank Reviewer 2 for their supportive remarks and positive evaluation of our study!

**Reviewer #3: 1. There is no complementation of any of the mutants evaluated in this study. For mutants for which major study conclusions are based, complementation should be demonstrated at least for in vitro (growth) phenotypes if not for in vivo UTI phenotypes. This is imperative to ensure the most important phenotypes are not due off target or polar effects.**

> We thank the reviewer for this important point. We now include *in vitro* complementation data, including empty vector controls, for key PTS mutants displaying measurable phenotypes (new Fig. S1 and lines 166-9). The *ptsH* growth defect in MinA was partially rescued by *ptsH in trans*, but fully restored with *ptsHI*, consistent with their expression as an operon. Critically, growth was restored in both glycerol and glucose carbon sources, and we discuss this in the context of the nitrogen-dependent growth phenotypes we reported in Fig. S5 (now Fig. S6, lines 351-4).
 For *xapB* and other mutants lacking measurable *in vitro* phenotypes, complementation is not feasible in this context. Some mutants (*e.g.*, *xapB*) appear monocistronic and are unlikely to exert polar effects; others (*e.g*., *ulaC*) were intentionally designed to disrupt entire operons to render the entire transport system non-functional; however, these mutations did not result in a detectable fitness defect *in vivo* during our In-seq experiment. Although we would ultimately like to understand the mechanisms behind all the *in vivo* defects we identified, this effort will require identification of the relevant substrates beyond glucose. Considering the technical and financial constraints of *in vivo* complementation studies, we have focused on validating the relevant *in vitro* defects that support our primary conclusions.

**2. There are several instances where data to support a conclusion is not provided. For example, the data supporting that there was no variation in input CFUs (line 180) is not provided/referenced in the manuscript. The authors should carefully revise the manuscript making sure to include data for all conclusion and that it is referenced appropriately in the manuscript (e.g. Biolog data is mentioned to in line 207 but there is no dataset referenced here.)**

>We note that the supporting data for line 180 (Datasets S1 and S2) are cited in the previous sentence; however, we have now explicitly mentioned the Input (I) data are in these datasets (now line 198). The full In-seq dataset is posted to GEO #GSE244606 and is referenced in line 849; we have now also added this accession number to the Results (line 195). The total input CFU values used for each pool are stated in the Methods (line 733: Group 1 = 2.51 x10^8^ CFU/ml; Group 2 = 2.68 x 10^8^ CFU/ml).
 For the Biolog experiments, many substrates did not allow for wild-type *P. mirabilis* growth. Other substrates produced artifacts due to chemical precipitation, which might be mistaken for bacterial growth (lines 261-2). For these reasons, we initially only included interpretable and relevant conditions used to support our conclusions into the presented figures. However, to address the reviewer’s valid concern, we have compiled all 864 growth curves (3 Biolog plates, 96-wells each, across 3 bacterial strains) into new Supplemental Dataset S3. This is now referenced in the corresponding Results sections (lines 238 and 309).

**3. Without comparing to double deletion strains, I am not sure if the authors can conclude that both ulaC and scrA along with ptsG are responsible for the ptsI/ptsH in vivo mutants. At the minimum comparisons to double ulaC/ptsG and scrA/ptsG double deletions would be needed to state this and ideally some sort of epistasis analysis would be performed to confirm that these genes are indeed acting in the same pathway as ptsH or ptsI. The phenotypes may be similar, but that does not necessarily mean they are acting through the same pathway.**

>Regarding the double mutant analysis, we concede that it is possible other PTS transporters have subtle phenotypes during UTI, which would require extensive additional experimentation to investigate. Because the triple mutant already demonstrated a clear, significant fitness defect *in vivo* and because additional mutant combinations would require substantial animal use, we chose not to expand this analysis in the current study. However, we lay out several lines of evidence that PtsG is specifically important (Fig. 7C & D) but not sufficient on its own to recapitulate the *ptsH* mutant phenotype (Figs. S3 and 7F). Because the triple *ulaC scrA ptsG* mutant demonstrated a clear, significant fitness defect *in vivo* that phenocopied the *ptsH* phenotype (Fig. 7E), and none of these three genes were identified in the In-seq screen (Fig. S3), we conclude that all three genes together contribute to *P. mirabilis* fitness during UTI (compared with attenuated fitness defect of the *scrA ptsG* double mutant in Fig. 7F). The overall features of PTS are well-characterized and conserved in diverse bacteria: PtsH and PtsI are key phosphorylating enzymes that act directly upon substrate-specific PTS enzyme II components. These concepts are now clarified in lines 413-6. Although *in silico* analysis is often not accurate at guessing specific carbohydrate substrates, the signatures for identity as PTS enzyme II components are clear. Teasing apart the combinatorial contributions of UlaC, ScrA, and PtsG to UTI fitness will be greatly aided by identifying the substrates for the first two transporters, a subject that we hope to revisit in future work (Discussion, lines 619-21).

**Part III – Minor Issues: Editorial and Data Presentation Modifications**

Reviewer #1: (No Response)

**Reviewer #2: Fig 8, S3, S8. I assume the horizontal lines indicates the median, but please check and add to the figure legend as appropriate.**

>Yes, thank you! The horizontal lines indeed represent the median values, and the omission has been corrected.

**Reviewer #3: 1. Please use exact p-values instead of asterisks throughout the manuscript. It is more transparent. Also please avoid using the language "trending towards significance". It is more useful to discuss why the current hypothesis test may have failed (low n-value due to dying mice at that experimental for example) than to suggest possible future signficance.**

>Because many of our figures are not conducive to displaying *P*-values without compromising readability, and the asterisk notation is widely understood in the field (including in a perusal of recent publications from *PLoS Pathogens*, such as this sampling from the April 2026 issue: PMID 41941531, 41941484, 41945616), we have retained asterisks in the figures, but have now ensured exact *P-*values for significant comparisons are reported in the Results. We removed the phrase “trending toward significance.” In the mentioned instance where differences did not reach statistical significance, we now report the observed effect sizes and provide additional context, such as reduced sample size due to increased morbidity at later timepoints may have limited statistical power (lines 475-8).

**2. Why was there a change in the duration of the UTI models between the pooled infection/InSeq experiment and the mutant versus wild-type competition experiments. There is no rationale provided for switching from a 24 hour to a 7 day long infection timescale.**

>The rationale for the differing infection durations is described in lines 216-8, but we have revised the text to make this more explicit (lines 213-5). Briefly, the pooled IN-seq experiment was limited to 24 hours to reduce bottleneck effects and stochastic loss of mutants, which are well-established limitations in pooled infection models of UTI [PMID: 32358013, 36377916]. In contrast, follow-up co-challenge experiments were performed using the standard 7-day infection model to assess colonization, persistence, and dissemination under conditions routinely used in this field.
 Importantly, the fitness defects identified in the pooled In-seq screen were recapitulated in the longer, traditional infection model. This concordance across experimental approaches strengthens the conclusions and supports the robustness of the identified fitness determinants, particularly given that validation rates in pooled mutagenesis studies are often limited by bottleneck effects. This is emphasized in the Discussion text lines 555-565.

**3. Line 72 needs a reference**

>References have been added (new refs 14-16).

**4. Line 97 - please clarify that the findings suggest elevated glucose may impact UTI outcomes in mice as there is not strong data supporting this yet in human cohorts**

> We have revised this statement in line 96 to clarify that these findings are based on preclinical data. We also have a statement in line 635-637 of the Discussion to highlight the inconclusive nature of SGLT2 inhibitor use in humans and corresponding relationships with UTI outcomes.

**5. For Figure 1A, either the functional gene names used in the text needed to be added to the figure or the locus tags used in the figure need to be referenced in the text. It is impossible to determine which ptsG or crr or ptsI, etc from how it is currently presented.**

> Gene names for the four significant growth defect strains have been added to Fig. 1A. All locus tags (PMI numbers) corresponding to functional gene names are provided in Table 1 (first mentioned in line 142), and relevant genes are now introduced in the text with both nomenclature systems at first mention. We have also added a reference to Table 1 in the Fig. 1A legend (line 175). We have retained PMI locus tags in the figure to ensure unambiguous gene identification and to avoid reinforcing potentially inconsistent functional annotations. Additionally, gene names of *ptsH*, *ptsI*, *crr*, and *ptsG* are located next to their corresponding PMI numbers in panels B and C of the same figure.

**6. The current figure 5 may be more well suited as a supplemental figure that can also be referenced earlier in the manuscript when PtsH, PtsI and Crr are first mentioned in line 161.**

>We appreciate this suggestion. We have retained Fig. 5 in the main text because it provides essential context for interpreting the relationship between PtsH/PtsI and substrate-specific PTS transporters as well as understanding our multi-mutant construction rationale. Placing this figure in the main text ensures that the functional hierarchy of the PTS system is clearly established for subsequent results.

**7. For current figures S1 and S2 please indicate which statistical test was used in the figure legends.**

>We have updated the figure legends for Figs. S1 and S2 (now S2 and S3) to specify the statistical test used. Differential abundance and associated *P-*values were calculated using the exactTest function implemented in edgeR, which is now cited in the statistical analysis section in the Methods (lines 838-9).

**8. Figure S3 data seems very important for interpreting CI data in figure 2 and should be included in the main text as part of figure 2. There should be plenty of room in the current figure 2 to add these data.**

>We have retained Fig. S3 (now S4) as a supplemental figure, as competitive index is the primary outcome measure for co-challenge experiments and is standard for presentation in the field. Raw CFU data are included to support interpretation and are referenced in the main text.

**9. Throughout the manuscript it is unclear if Biolog results represent growth or metabolic activity. The primary output of Biolog phenotype arrays is usually metabolic activity via reduction of a tetrazolium-based redox dye. Is this what the authors are reporting or is it the OD600 (growth) in the absence of the dye?**

> In our experiments, we measured bacterial growth using OD_600_ and did not include the tetrazolium redox dye. We have now clarified the Methods to specifically state that the omitted respiration substrate referred to the tetrazolium-based dye (line 813).

**10. Please include the recipe for Minimal A in the methods section instead of just a reference.**

>We have updated the Methods section to include the full recipe for Minimal A medium (lines 692-4) rather than solely citing the original reference.

**11. Figure S4 A and B look like two individual runs of the same experiment. These should be combined into a single graph that should be included in the main text in figure 4. Also there is no data presented to support the statement that no CFUs were recovered at the endpoint in line 223.**

>We have retained Fig. S4A and S4B (now Fig. S5A & B) as separate panels, as they represent independent biological replicates with substantial variability. Presenting them individually allows for transparent visualization of experimental reproducibility without obscuring the data. We have clarified this in the figure legend. The data in Fig. 4 reflect controlled experiments that confirm our prior published prediction that *P. mirabilis* is unable to use guanine or guanosine as a sole carbon source [PMID: 37293055]. We have left the Biolog data as a supplement because the precipitate-driven turbidity was not reflective of growth and was therefore misleading. Regarding line 223 (now 263), given the precipitate-driven turbidity in the guanosine wells of the Biolog plates, we specifically plated the contents of these wells on LB agar and obtained no outgrowth whatsoever. We now specify that these specific well contents were plated (lines 262-4).

**12. The red/yellow arrows in the current figure 5 are confusing and it would be helpful if they could be explained in the legend or in the text.**

> We have clarified the meaning of the red and yellow arrows in Fig. 5. These arrows denote PTS transporter systems grouped based on prior *in vivo* transcriptomic data shown in Fig 7A, with red indicating high induction and yellow indicating low expression/no induction during experimental UTI. This explanation has been further clarified in the figure legend and added to the main text (lines 369-70).

**13. The references in line 266 need to be corrected. Reference 48 is in Proteus vulgaris and not Proteus mirabilis so it should be removed. Instead the authors should reference https://doi.org/10.3389/fcimb.2021.803409 which demonstrated degradation and utilization of chondroitin sulfate for growth by multiple strains of Proteus mirabilis.**

>The text has been revised to more accurately reflect the literature (lines 325-30). Rather than removing the *Proteus vulgaris* reference, we now frame these studies as foundational work demonstrating chondroitinase activity across the *Proteus* genus, including early reports in both *P. vulgaris* and *P. mirabilis* (refs 52-55). We have also incorporated more recent findings as requested showing that clinical *P. mirabilis* isolates can degrade chondroitin sulfate under multiple conditions (refs 56-57).

**14. The data in Figure S5 seem like they should be included in the main text. It would also be important to give a more detailed explanation for the conclusion on line 280 - for example, what is the nitrogen source in Minimal a and why exactly does this explain the glucose defects in that medium?**

> We have retained Fig. S5 (now S6) as a supplemental figure because these data represent a secondary finding related to nitrogen source utilization. The nitrogen source in Minimal A is ammonium sulfate, which is now explicitly included in the Methods in response to a previous comment. We have also revised the text (lines 351-4) to more clearly explain how the Fig. S6 data relate to the Minimal A growth phenotypes and we highlight the new complementation data (new Fig. S1) where *ptsH* and *ptsI* growth in glycerol was restored. Specifically, we now clarify that the more severe defects observed for *ptsH* and *ptsI* (*vs. ptsG* and *crr*) in glucose likely reflect a cumulative effect of impaired glucose import and nitrogen assimilation. Additional interpretation of the nitrogen-related phenotype is included in the Discussion (lines 622-30).

**15. Overall, there is a difference between a given sugar not being a PTS substrate and it not being able to serve as a sole carbon source. Just because it cannot act as a sole carbon source does not necessarily mean it is not a substrate for a given PTS system. Perhaps in a different condition, it would stimulate growth and this growth would be reduced by deletion of that PTS component. I think maybe some of the conclusions could be updated with this point in mind.**

> We agree with this point and note that this distinction was acknowledged for xanthosine where we stated that failure of xanthosine to serve as a sole carbon or nitrogen source does not exclude the possibility of import (lines 242-3). To further address this concern, we have now revised the Results (lines 440-2) and Discussion (lines 612-7) to distinguish between substrate utilization and transport for PTS importers. This clarification does not alter our conclusions that these transporters are required for fitness in mice, regardless of their precise substrates.

PLOS authors have the option to publish the peer review history of their article (what does this mean?). If published, this will include your full peer review and any attached files.

If you choose “no”, your identity will remain anonymous but your review may still be made public.

**Do you want your identity to be public for this peer review?** For information about this choice, including consent withdrawal, please see our Privacy Policy.

Reviewer #1: No

Reviewer #2: **Yes:** Mark Schembri

Reviewer #3: **Yes:** Nicole Janell De Nisco
