# Supplementary material for: Selective sugar transport supports Proteus mirabilis fitness in the urinary tract
Source: PLoS Pathog. 2026 Jun 23;22(6):e1014324. doi: 10.1371/journal.ppat.1014324 (PMC13289894; doi:10.1371/journal.ppat.1014324)
Supplement: Peer review history

# Peer review: Decision Letter 0

**DOI:** 10.1371/journal.ppat.1014324.r001

**Authors:** D. Scott Samuels, Matthew Mulvey

6 Apr 2026

-->PPATHOGENS-D-26-00451

Selective sugar transport supports Proteus mirabilis fitness in the urinary tract

PLOS Pathogens

Dear Dr. Pearson,

Thank you for submitting your manuscript to PLOS Pathogens. Your manuscript was evaluated by members of the editorial board and three external reviewers. All agree that it has potential to be an important contribution, but there are concerns that need to be addressed. Therefore, we invite you to submit a substantially revised version of the manuscript that addresses all of the points raised by all three reviewers. Reviewers 1 and 3, in particular, raise a number of points that should be addressed, including the genetic complementation of key mutants in at least some assays.

Please submit your revised manuscript by Jun 05 2026 11:59PM. If you will need more time than this to complete your revisions, please reply to this message or contact the journal office at plospathogens@plos.org. When you're ready to submit your revision, log on to https://www.editorialmanager.com/ppathogens/ and select the 'Submissions Needing Revision' folder to locate your manuscript file.

Please include the following items when submitting your revised manuscript:

* A letter that responds to each point raised by the editor and reviewer(s). You should upload this letter as a separate file labeled 'Response to Reviewers'. This file does not need to include responses to any formatting updates and technical items listed in the 'Journal Requirements' section below.

* A marked-up copy of your manuscript that highlights changes made to the original version. You should upload this as a separate file labeled 'Revised Manuscript with Track Changes'.

* An unmarked version of your revised paper without tracked changes. You should upload this as a separate file labeled 'Manuscript'.

If you would like to make changes to your financial disclosure, competing interests statement, or data availability statement, please make these updates within the submission form at the time of resubmission. Guidelines for resubmitting your figure files are available below the reviewer comments at the end of this letter.

We look forward to receiving your revised manuscript.

Kind regards,

Matthew A Mulvey, PhD

Academic Editor

PLOS Pathogens

D. Scott Samuels

Section Editor

PLOS Pathogens

Sumita Bhaduri-McIntosh

Editor-in-Chief

PLOS Pathogens

orcid.org/0000-0003-2946-9497

Michael Malim

Editor-in-Chief

PLOS Pathogens

orcid.org/0000-0002-7699-2064

**Journal Requirements:**

1) Please upload all main figures as separate Figure files in .tif or .eps format. For more information about how to convert and format your figure files please see our guidelines:

https://journals.plos.org/plospathogens/s/figures

2) We notice that your supplementary figures are uploaded with the file type 'Figure'. Please amend the file type to 'Supporting Information'. Please ensure that each Supporting Information file has a legend listed in the manuscript after the references list.

3) Please update your Data Availability Statement in the online submission form to include the reviewer token as stated in the manuscript.

4) In the online submission form, you indicated that "All strains and plasmids are available upon reasonable request. " All PLOS journals now require all data underlying the findings described in their manuscript to be freely available to other researchers, either

1. In a public repository

2. Within the manuscript itself

3. Uploaded as supplementary information.

This policy applies to all data except where public deposition would breach compliance with the protocol approved by your research ethics board. If your data cannot be made publicly available for ethical or legal reasons (e.g., public availability would compromise patient privacy), please explain your reasons by return email and your exemption request will be escalated to the editor for approval. Your exemption request will be handled independently and will not hold up the peer review process, but will need to be resolved should your manuscript be accepted for publication. One of the Editorial team will then be in touch if there are any issues.

5) Some material included in your submission may be copyrighted. According to PLOS’s copyright policy, authors who use figures or other material (e.g., graphics, clipart, maps) from another author or copyright holder must demonstrate or obtain permission to publish this material under the Creative Commons Attribution 4.0 International (CC BY 4.0) License used by PLOS journals. Please closely review the details of PLOS’s copyright requirements here: PLOS Licenses and Copyright. If you need to request permissions from a copyright holder, you may use PLOS's Copyright Content Permission form.

Please respond directly to this email and provide any known details concerning your material's license terms and permissions required for reuse, even if you have not yet obtained copyright permissions or are unsure of your material's copyright compatibility. Once you have responded and addressed all other outstanding technical requirements, you may resubmit your manuscript within Editorial Manager.

Potential Copyright Issues:

Figure 2A. Please confirm whether you drew the images / clip-art within the figure panels by hand. If you did not draw the images, please provide (a) a link to the source of the images or icons and their license / terms of use; or (b) written permission from the copyright holder to publish the images or icons under our CC BY 4.0 license. Alternatively, you may replace the images with open source alternatives. See these open source resources you may use to replace images / clip-art:

- https://commons.wikimedia.org

- https://openclipart.org/.

Note: If the reviewer comments include a recommendation to cite specific previously published works, please review and evaluate these publications to determine whether they are relevant and should be cited. There is no requirement to cite these works unless the editor has indicated otherwise.

**Reviewers' Comments:**

Reviewer's Responses to Questions

**Part I - Summary**

Please use this section to discuss strengths/weaknesses of study, novelty/significance, general execution and scholarship.

Reviewer #1: This manuscript from Shea et al. addresses the role of sugar import systems in the fitness of P. mirabilis, a clinically significant uropathogen. Specifically, they generated 47 mutants lacking various sugar transport systems and tested the role of these genes in fitness under various in vitro conditions and in the mouse model of urinary tract infection. This work unravels the role of several new fitness factors that are involved in the pathogenesis of UTI caused by P. mirabilis. They demonstrate that increased urine glucose, simulating diabetic urinary tract, enhances pathogen colonization in a complex mechanism since there is inverse correlation between urinary glucose levels and bacterial colonization. This Reviewer finds the use of InSeq for in vivo fitness assessment as a robust quantitative approach that overcomes the limitations of bottleneck in the infection model. Their results also raise an important point that genome annotations must be experimentally validated and might not be accurate as presented in databases. The experimental design is rigorous. Methods are described in sufficient detail. The manuscript is well written and organized logically. This work will be of interest to researchers in the fields of host-pathogen interaction and diabetic complications.

Reviewer #2: This manuscript presents a comprehensive investigation into carbohydrate import systems relevant to Proteus mirabilis infection of the urinary tract. Some highlights include the generation of 47 targeted mutants in predicted sugar transporters, screening of these mutants in vitro to evaluate gene function, the use of pooled In seq and co challenge experiments to determine in vivo function, and evaluation of the impact of glucosuria using an SGLT2 inhibitor model. This impressive amount of work led to multiple findings, including the identification of ptsH, ptsI, and xapB as critical fitness genes, the discovery that many transporter genes have been misannotated in P. mirabilis, and the demonstration that induced glucosuria exacerbates P. mirabilis colonization.

Reviewer #3: Here the authors evaluate the contribution of genes predicted to be involved in sugar transport to Proteus mirabilis HI4320 urinary tract infection in a mouse model. Using a pooled infection/InSeq approach, the authors identify genes whose loss results in various colonization defects in the mouse UTI model. The authors attempt to identify substrates for various predicted transport systems but are largely unsuccessful as many of the predicted substrates did not support growth when provided as a sole carbon source leading to mostly inconclusive results. However the authors did identify growth defects for the ptsH mutant on several carbon and nitrogen sources. To try to understand the pathway through which ptsH was acting the authors tested the fitness of triple mutants in PTS systems and identified colonization defects for the ulaA, ptsG and scrA triple mutant. Finally the authors investigated the impact of glucosuria on P. mirabilis colonization in their mouse UTI model and fitness of the ptsH mutant using dapagliflozin. Mice treated with dapagliflozin experienced more severe P. mirabilis UTI and that the ptsH mutant was less competitive in colonization at some experimental endpoints. Overall, this work identifies sugar transport systems contributing to P. mirabilis UTI and examines the effects of SGLT-2 induced glucosuria on P. mirabilis pathogenesis.

**********

**Part II – Major Issues: Key Experiments Required for Acceptance**

Please use this section to detail the key new experiments or modifications of existing experiments that should be absolutely required to validate study conclusions.

Generally, there should be no more than 3 such required experiments or major modifications for a "Major Revision" recommendation. If more than 3 experiments are necessary to validate the study conclusions, then you are encouraged to recommend "Reject".

Reviewer #1: Have the Authors investigated the growth of sugar transport mutants in human urine, and urine supplemented with glucose to resemble diabetic urine? This seems like a straightforward experiment that could yield critical insights to strengthen this manuscript.

Do the mutants with severe in vivo fitness defect exhibit changes in known virulence factors such as urease? Adding this information is needed to clarify decreased fitness due to poor in vivo growth as opposed to decreased pathogenicity.

There seems to be bacterial growth in Fig. 3A without a C source? This should be explained.

Fig 8 A&B: Baseline glucose (day -1) is almost double in panel A, compared to B, and also post-treatment differences between these panels. Potential reasons for this change should be included.

Fig. 7A: Error bars should be included to inform the readers of the range of data points

Fig. 1A: Please add gene names at least for the ones that have growth defects.

Reviewer #2: Overall, the data presented are sound, the manuscript is very well written, and the work will be of broad interest to researchers studying urinary tract infections, bacterial metabolism, and host-pathogen interactions.

I have no substantive concerns regarding the data or major conclusions. I cannot fault the work and congratulate the authors on an excellent study.

Reviewer #3: 1. There is no complementation of any of the mutants evaluated in this study. For mutants for which major study conclusions are based, complementation should be demonstrated at least for in vitro (growth) phenotypes if not for in vivo UTI phenotypes. This is imperative to ensure the most important phenotypes are not due off target or polar effects.

2. There are several instances where data to support a conclusion is not provided. For example, the data supporting that there was no variation in input CFUs (line 180) is not provided/referenced in the manuscript. The authors should carefully revise the manuscript making sure to include data for all conclusion and that it is referenced appropriately in the manuscript (e.g. Biolog data is mentioned to in line 207 but there is no dataset referenced here.)

3. Without comparing to double deletion strains, I am not sure if the authors can conclude that both ulaC and scrA along with ptsG are responsible for the ptsI/ptsH in vivo mutants. At the minimum comparisons to double ulaC/ptsG and scrA/ptsG double deletions would be needed to state this and ideally some sort of epistasis analysis would be performed to confirm that these genes are indeed acting in the same pathway as ptsH or ptsI. The phenotypes may be similar, but that does not necessarily mean they are acting through the same pathway.

**********

**Part III – Minor Issues: Editorial and Data Presentation Modifications**

Please use this section for editorial suggestions as well as relatively minor modifications of existing data that would enhance clarity.

Reviewer #1: (No Response)

Reviewer #2: Fig 8, S3, S8. I assume the horizontal lines indicatesthe median, but please check and add to the figure legend as appropriate.

Reviewer #3: 1. Please use exact p-values instead of asterisks throughout the manuscript. It is more transparent. Also please avoid using the language "trending towards significance". It is more useful to discuss why the current hypothesis test may have failed (low n-value due to dying mice at that experimental for example) than to suggest possible future signficance.

2. Why was there a change in the duration of the UTI models between the pooled infection/InSeq experiment and the mutant versus wild-type competition experiments. There is no rationale provided for switching from a 24 hour to a 7 day long infection timescale.

3. Line 72 needs a reference

4. Line 97 - please clarify that the findings suggest elevated glucose may impact UTI outcomes in mice as there is not strong data supporting this yet in human cohorts

5. For Figure 1A, either the functional gene names used in the text needed to be added to the figure or the locus tags used in the figure need to be referenced in the text. It is impossible to determine which ptsG or crr or ptsI, etc from how it is currently presented.

6. The current figure 5 may be more well suited as a supplemental figure that can also be referenced earlier in the manuscript when PtsH, PtsI and Crr are first mentioned in line 161.

7. For current figures S1 and S2 please indicate which statistical test was used in the figure legends.

8. Figure S3 data seems very important for interpreting CI data in figure 2 and should be included in the main text as part of figure 2. There should be plenty of room in the current figure 2 to add these data.

9. Throughout the manuscript it is unclear if Biolog results represent growth or metabolic activity. The primary output of Biolog phenotype arrays is usually metabolic activity via reduction of a tetrazolium-based redox dye. Is this what the authors are reporting or is it the OD600 (growth) in the absence of the dye?

10. Please include the recipe for Minimal A in the methods section instead of just a reference.

11. Figure S4 A and B look like two individual runs of the same experiment. These should be combined into a single graph that should be included in the main text in figure 4. Also there is no data presented to support the statement that no CFUs were recovered at the endpoint in line 223.

12. The red/yellow arrows in the current figure 5 are confusing and it would be helpful if they could be explained in the legend or in the text.

13. The references in line 266 need to be corrected. Reference 48 is in Proteus vulgaris and not Proteus mirabilis so it should be removed. Instead the authors should reference https://doi.org/10.3389/fcimb.2021.803409 which demonstrated degradation and utilization of chondroitin sulfate for growth by multiple strains of Proteus mirabilis.

14. The data in Figure S5 seem like they should be included in the main text. It would also be important to give a more detailed explanation for the conclusion on line 280 - for example, what is the nitrogen source in Minimal a and why exactly does this explain the glucose defects in that medium?

15. Overall, there is a difference between a given sugar not being a PTS substrate and it not being able to serve as a sole carbon source. Just because it cannot act as a sole carbon source does not necessarily mean it is not a substrate for a given PTS system. Perhaps in a different condition, it would stimulate growth and this growth would be reduced by deletion of that PTS component. I think maybe some of the conclusions could be updated with this point in mind.

**********

PLOS authors have the option to publish the peer review history of their article (what does this mean?). If published, this will include your full peer review and any attached files.

If you choose “no”, your identity will remain anonymous but your review may still be made public.

**Do you want your identity to be public for this peer review?**  For information about this choice, including consent withdrawal, please see our Privacy Policy.

Reviewer #1: No

Reviewer #2: **Yes:**  Mark Schembri

Reviewer #3: **Yes:**  Nicole Janell De Nisco

[NOTE: If reviewer comments were submitted as an attachment file, they will be attached to this email and accessible via the submission site. Please log into your account, locate the manuscript record, and check for the action link "View Attachments". If this link does not appear, there are no attachment files.]

**Figure resubmission:**

-->While revising your submission, we strongly recommend that you use PLOS’s NAAS tool (https://ngplosjournals.pagemajik.ai/artanalysis) to test your figure files. NAAS can convert your figure files to the TIFF file type and meet basic requirements (such as print size, resolution), or provide you with a report on issues that do not meet our requirements and that NAAS cannot fix.

After uploading your figures to PLOS’s NAAS tool - https://ngplosjournals.pagemajik.ai/artanalysis, NAAS will process the files provided and display the results in the "Uploaded Files" section of the page as the processing is complete. If the uploaded figures meet our requirements (or NAAS is able to fix the files to meet our requirements), the figure will be marked as "fixed" above. If NAAS is unable to fix the files, a red "failed" label will appear above. When NAAS has confirmed that the figure files meet our requirements, please download the file via the download option, and include these NAAS processed figure files when submitting your revised manuscript.

**Reproducibility:**

To enhance the reproducibility of your results, we recommend that authors of applicable studies deposit laboratory protocols in protocols.io, where a protocol can be assigned its own identifier (DOI) such that it can be cited independently in the future. Additionally, PLOS ONE offers an option to publish peer-reviewed clinical study protocols. Read more information on sharing protocols at https://plos.org/protocols?utm_medium=editorial-email&utm_source=authorletters&utm_campaign=protocols

# Author comment: Author response to Decision Letter 1

**DOI:** 10.1371/journal.ppat.1014324.r002

20 May 2026

Attachment Submitted filename: response to reviews.docx

# Editor report: Decision Letter 1

**DOI:** 10.1371/journal.ppat.1014324.r003

**Authors:** D. Scott Samuels, Matthew Mulvey, D. Scott Samuels, Matthew Mulvey

29 May 2026

Dear Dr. Pearson,

We are pleased to inform you that your manuscript 'Selective sugar transport supports Proteus mirabilis fitness in the urinary tract' has been provisionally accepted for publication in PLOS Pathogens.

Before your manuscript can be formally accepted you will need to complete some formatting changes, which you will receive in a follow up email. A member of our team will be in touch with a set of requests.

Please note that your manuscript will not be scheduled for publication until you have made the required changes, so a swift response is appreciated.

IMPORTANT: The editorial review process is now complete. PLOS will only permit corrections to spelling, formatting or significant scientific errors from this point onwards. Requests for major changes, or any which affect the scientific understanding of your work, will cause delays to the publication date of your manuscript.

Should you, your institution's press office or the journal office choose to press release your paper, you will automatically be opted out of early publication. We ask that you notify us now if you or your institution is planning to press release the article. All press must be co-ordinated with PLOS.

Thank you again for supporting Open Access publishing; we are looking forward to publishing your work in PLOS Pathogens.

Best regards,

Matthew A Mulvey, PhD

Academic Editor

PLOS Pathogens

D. Scott Samuels

Section Editor

PLOS Pathogens

Sumita Bhaduri-McIntosh

Editor-in-Chief

PLOS Pathogens

orcid.org/0000-0003-2946-9497

Michael Malim

Editor-in-Chief

PLOS Pathogens

orcid.org/0000-0002-7699-2064

***********************************************************

Reviewer Comments (if any, and for reference):

# Editor report: Acceptance letter

**DOI:** 10.1371/journal.ppat.1014324.r004

**Authors:** D. Scott Samuels, Matthew Mulvey, D. Scott Samuels, Matthew Mulvey

Dear Dr. Pearson,

We are delighted to inform you that your manuscript, "Selective sugar transport supports Proteus mirabilis fitness in the urinary tract," has been formally accepted for publication in PLOS Pathogens.

We have now passed your article onto the PLOS Production Department who will complete the rest of the pre-publication process. All authors will receive a confirmation email upon publication.

The corresponding author will soon be receiving a typeset proof for review, to ensure errors have not been introduced during production. Please review the PDF proof of your manuscript carefully, as this is the last chance to correct any scientific or type-setting errors. Please note that major changes, or those which affect the scientific understanding of the work, will likely cause delays to the publication date of your manuscript. Note: Proofs for Front Matter articles (Pearls, Reviews, Opinions, etc...) are generated on a different schedule and may not be made available as quickly.

Soon after your final files are uploaded, the early version of your manuscript, if you opted to have an early version of your article, will be published online. The date of the early version will be your article's publication date. The final article will be published to the same URL, and all versions of the paper will be accessible to readers.

For Research Articles, you will receive an invoice from PLOS for your publication fee after your manuscript has reached the completed accept phase. If you receive an email requesting payment before acceptance or for any other service, this may be a phishing scheme. Learn how to identify phishing emails and protect your accounts at https://explore.plos.org/phishing.

Thank you again for supporting open-access publishing; we are looking forward to publishing your work in PLOS Pathogens.

Best regards,

Sumita Bhaduri-McIntosh

Editor-in-Chief

PLOS Pathogens

orcid.org/0000-0003-2946-9497

Michael Malim

Editor-in-Chief

PLOS Pathogens

orcid.org/0000-0002-7699-2064
